# Osteoarthritis and sarcopenia-related traits: the cross-sectional study from NHANES 2011–2014 and Mendelian randomization study

**DOI:** 10.1186/s13018-023-03960-w

**Published:** 2023-07-15

**Authors:** Shuai Chen, Huawei Han, Jie Jin, Guowei Zhou, Zhiwei Li

**Affiliations:** 1 Department of Orthopaedics, The Second Affiliated Hospital of Nanjing University of Chinese Medicine, No. 23, Nanhu Road, Jianye District, Nanjing, 210017 Jiangsu Province People’s Republic of China; 2 Department of General Surgery, Jiangsu Province Hospital of Chinese Medicine, Affiliated Hospital of Nanjing University of Chinese Medicine, Nanjing, People’s Republic of China

**Keywords:** Osteoarthritis, Sarcopenia, Grip strength, Mendelian randomization, NHANES

## Abstract

**Background:**

Osteoarthritis (OA) and sarcopenia are common musculoskeletal disorders in the aged population, and a growing body of evidence indicated that they mutually influence one another. Nevertheless, there was still substantial controversy and uncertainty about the causal relationship between sarcopenia and OA. We explored the complex association between sarcopenia-related traits and OA using cross-sectional analysis and Mendelian randomization (MR).

**Methods:**

The cross-sectional study used the data from the National Health and Nutrition Examination Survey (NHANES) 2011–2014. Weighted multivariable-adjusted logistic regression and subgroup analyses were used to evaluate the correlation between sarcopenia, grip, appendicular lean mass (ALM) and the risk of OA. Then, we further performed MR analysis to examine the causal effect of sarcopenia-related traits (grip strength, ALM) on OA. Instrumental variables for grip strength and ALM were from the UK Biobank, and the summary-level data for OA was derived from the Genetics of Osteoarthritis (GO) Consortium GWAS (*n* = 826,690).

**Results:**

In this cross-sectional analysis, we observed that sarcopenia, grip were significantly linked with the risk of OA (OR 1.607, 95% CI 1.233–2.094, *P* < 0.001), (OR 0.972, 95% CI 0.964–0.979, *P* < 0.001). According to subgroup analyses stratified by gender, body mass index (BMI), and age, the significant positive relationship between sarcopenia and OA remained in males, females, the age (46–59 years) group, and the BMI (18.5–24.9 kg/m^2^) group (*P* < 0.05). Furthermore, MR analysis and sensitivity analyses showed causal associations between right grip, left grip and KOA (OR 0.668; 95% CI 0.509 to 0.877; *P* = 0.004), (OR 0.786; 95% CI 0.608 to 0.915; *P* = 0.042). Consistent directional effects for all analyses were observed in both the MR-Egger and weighted median methods. Subsequently, sensitivity analyses revealed no heterogeneity, directional pleiotropy or outliers for the causal effect of grip strength on KOA (*P* > 0.05).

**Conclusions:**

Our research provided evidence that sarcopenia is correlated with an increased risk of OA, and there was a protective impact of genetically predicted grip strength on OA. These findings needed to be verified in further prospective cohort studies with a large sample size.

**Supplementary Information:**

The online version contains supplementary material available at 10.1186/s13018-023-03960-w.

## Introduction

Osteoarthritis (OA) is a whole joint disease impacting all joint tissues and is characterized by pain, joint stiffness, deformity and dysfunction and is one of the leading causes of global disability [1, 2]. According to the World Health Organization, approximately 300 million individuals worldwide are affected by OA, and about 10% of men and 18% of women suffer from symptomatic OA [3]. Apart from that, as the population ages and the proportion of obese people increases, the high incidence and high disability of osteoarthritis also bring a huge economic burden to society [4].

Sarcopenia is also an age-related senile syndrome of decreased muscle strength and limited physical function [5, 6]. The atrophy or weakness of the muscles themselves may be caused by the biomechanical impact of changed bone and periarticular muscle cross-talk, which can result in the development and progression of OA [7–9]. Currently, existing epidemiological studies have investigated the relationship between sarcopenia and OA, and some reports suggested that sarcopenia and OA may coexist in the elderly. According to a longitudinal cohort study, lower limb muscle strength and muscle mass were related to the incidence of knee OA (KOA), and patients with sarcopenia were more likely to have symptomatic KOA than those without sarcopenia [10]. In addition, several studies have shown that the decrease in quadriceps muscle strength can exacerbate knee pain and articular cartilage damage in patients with KOA, suggesting that decreased lower limb skeletal muscle mass is an independent risk factor for the prevalence of KOA [11, 12]. Nevertheless, these studies are mainly based on observational cross-sectional analyses, and it is still not clear whether there is a causal link between sarcopenia-related traits and OA.

Therefore, to investigate the correlation between sarcopenia and OA, we first conducted an observational study with data based on the US population from the National Health and Nutrition Examination Survey (NHANES) database. Furthermore, we performed a two-sample Mendelian randomization (MR) analysis to reveal the causal effect of sarcopenia-related traits on the risk of osteoarthritis from the level of genetic variation. MR analysis is an epidemiological data analysis approach that utilizes genetic variation as an instrumental variable of exposure to assess the causal relationship between exposure factors and outcome events [13, 14], and with the discovery of large numbers of genetic variants strongly associated with specific traits, and with many large sample genome-wide association studies (GWAS) publicly releasing hundreds of thousands of aggregated data on exposures and disease associations with genetic variants. In recent years, MR analysis has gained widespread popularity for determining unbiased causal relationships between exposures and various diseases [15].

## Materials and methods

### The cross-sectional analysis

#### Study population

All data in this study were obtained from NHANES, a cross-sectional survey that uses a complex, multistage, and stratified probability sampling method to obtain nationally representative health and nutrition data for the noninstitutionalized US population. In addition, all NHANES protocols were approved by the National Center for Health Statistics Research Ethics Review Board, and informed consent was obtained from all participants [16].

**Fig. 1:**
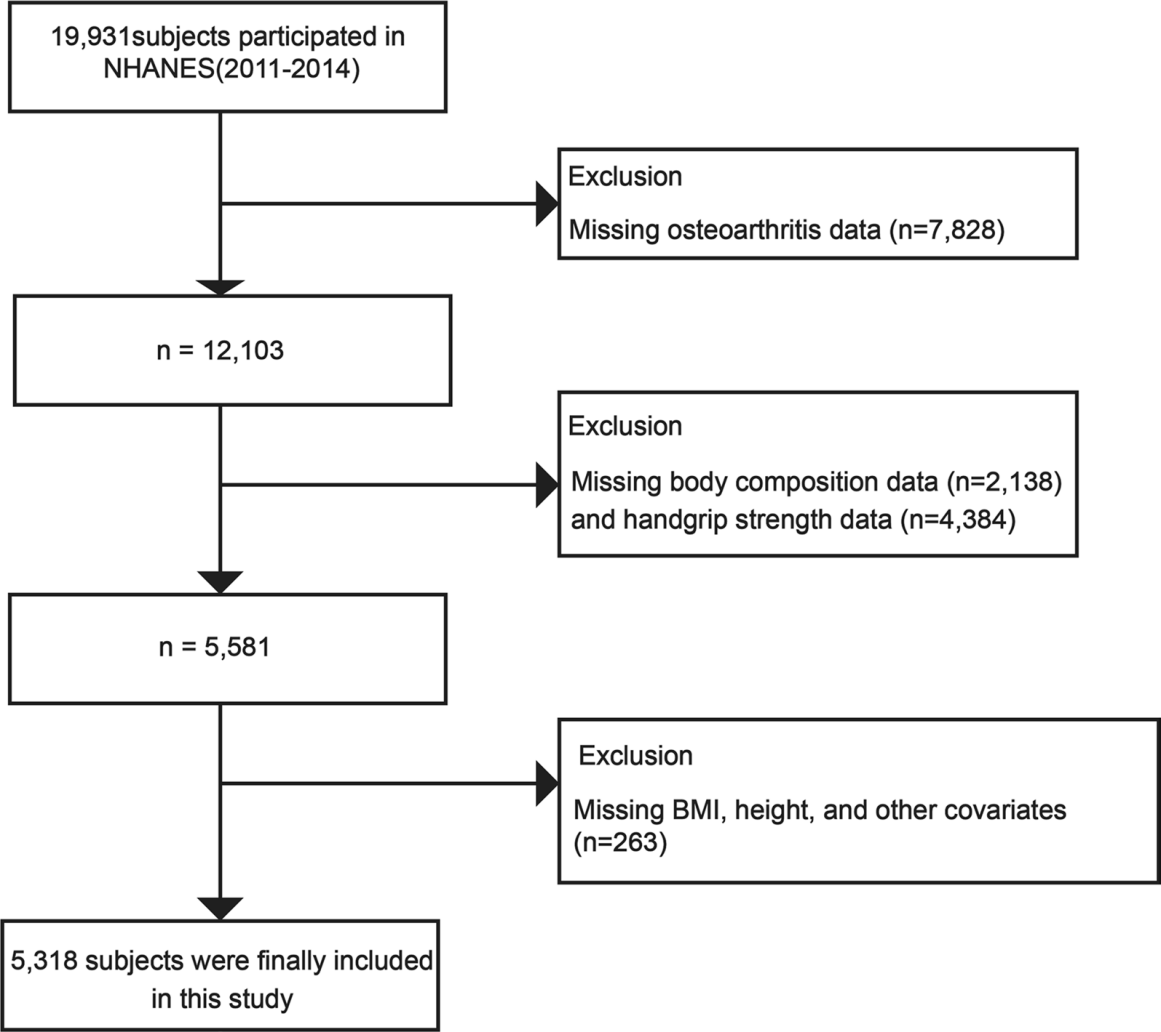
Study flowchart. NHANES, National Health and Nutrition Examination Survey

For our study, data were selected in two cycles of the NHANES survey (2011–2012 and 2013–2014). In total, there were 19,931 participants who completed demographic survey, laboratory examination, and health condition questionnaires. The exclusion criteria were as follows: (1) Missing osteoarthritis data (*n* = 7828); (2) Missing body composition data (*n* = 2138) and hand grip strength data (*n* = 4384); (3) Missing BMI, height, and other covariates (*n* = 263). Ultimately, a total of 5318 participants were recruited in this analysis (Fig. 1).

#### Diagnosis of osteoarthritis

Osteoarthritis diagnosis data was from the “Medical Conditions” questionnaire section of the NHANES. First of all, participants were asked if doctor ever said they had arthritis. If they answered “yes,” they would be further asked to identify “which type of arthritis was it” (The arthritis was classified as osteoarthritis, rheumatoid arthritis, psoriatic arthritis, and others based on NHANES questionnaire data) [17].

#### Definition of body composition variables and sarcopenia

In the NHANES, dual-energy X-ray absorptiometry (DXA) was used to assess the body composition. Among them, appendicular lean mass (ALM) was determined as the sum of four limbs’ muscle mass [18]. In addition, the criterion result of the total grip strength (kg) was measured with a dynamometer. Participants were asked to stand up and grasp the dynamometer as firmly as possible with one hand. Each hand was examined three times, with a 60-s rest alternating between two measurements on the same hand. The sum of the maximum grip strength of each hand was determined as the combined grip strength [19].

Sarcopenia was defined following the Foundation for the National Institutes of Health (FNIH) definition using the ALM and body mass index ratio (ALM:BMI). For men and women, the cut values for sarcopenia were, respectively, < 0.789 kg/m^2^ and < 0.512 kg/m^2^. In addition, grip strength was also recommended as an effective simple measure of sarcopenia, and sarcopenia was defined as hand grip strength < 28 kg for men and < 18 kg for women [20].

#### Other covariates

The covariates are demographic data, examination data, laboratory data, and questionnaire data. Demographic data included age (years, range 20 to 59, average: 38.88) [21], gender (male and female), race, level of education (less than high school, high school, more than high school), race (Mexican American, other race, Non-Hispanic white, and Non-Hispanic black). Examination data included weight (kg), height (cm), waist circumference (WC, cm) [22], and BMI (kg/m^2^) [23]. Laboratory data covered blood urea nitrogen (BUN, mmol/L) [24], total calcium (Ca, mmol/L) [25], phosphorus (P, mmol/L) [26], triglycerides (TG, mmol/L) [27], total cholesterol (TC, mmol/L) [28], creatinine (Cr, µmol/L) [29], and uric acid (UA, µmol/L) [30]. As a final point, questionnaire data included information on smoking behavior (Yes/No) [31], alcohol consumption (Yes/No) [32], hypertension (Yes/No) [33], and diabetes (Yes/No) [34].

#### Statistical analyses

Our categorical variables were expressed as percentages, and our continuous variables were expressed as means and standard deviations. The Shapiro–Wilk test and the Kolmogorov–Smirnov test are to test normality of the distribution for each continuous variables. Firstly, the regular T test was used to compare the baseline characteristics of the participants with and without OA. Multicollinearity diagnostic was performed to check whether there was multicollinearity between the covariates and to exclude covariates with variance inflation factor (VIF) values greater than 10. Then, to further investigate the association between independent variables and dependent variables, we carried out multiple regressions. In the models of multivariate linear regression, an unadjusted model (Model 1) was first established, followed by an adjusted model (Model 2) that took age, gender and race/ethnicity into consideration. Then, a fully adjusted model (Model 3) was then calculated using variables such as age, gender, race/ethnicity, smoking behavior, alcohol consumption, hypertension, diabetes, BUN, Ca, P, TC, TG, Cr, and UA. Moreover, we stratified the data by gender, BMI, and age to examine the robustness of the association. R software (version 4.1.3) and Empower Stats (version 2.0) were used for all analyses.

### Mendelian randomization analysis

#### Genome-Wide Association Studies Sources

According to the consensus of the European Working Group on Sarcopenia in Older People (EWGSOP), muscle mass and hand grip strength were used as criteria for diagnosing sarcopenia. GWAS summary data for ALM and hand grip strength were obtained from the UK Biobank study. An analysis of 450,243 UK Biobank cohort participants was conducted to quantify ALM-related values by summing fat-free mass, and adjusted for age, sex, the 10 most important principal components, and other covariates [35]. For hand grip strength, UK Biobank provided GWAS summary statistics on right- and left-hand grip strength based on 461,089 and 461,026 United Kingdom people, respectively [36]. A calibrated grip strength device adjusted for hand size was used to measure the grip strength, and each SNP was evaluated for a link with hand grip strength after adjusting for age, sex, and other variables. However, due to the lack of demographic data in the original GWAS study, therefore, we were unable to perform a subgroup analysis for factors such as gender and age.

Given that both hip and knee are common sites for OA in clinic, there are three sources of OA data in this study, including total OA, KOA, and hip osteoarthritis (HOA). The summary data of OA were derived from the Genetics of Osteoarthritis (GO) Consortium GWAS, which included 826,690 individuals from nine European populations [37]. Detailed information on the demographic characteristics of selected summary-level GWASs applied in the MR study is shown in Additional file 1: Table S1.

#### Selection of genetic instrumental variables

(1) The instrumental variables selected for analysis are highly related to the corresponding exposures (*P* < 5*10^–8^). (2) The instrumental variables are mutually independent and avoid the offset caused by linkage disequilibrium (LD) between the SNPs (r^2^ < 0.001, LD distance > 10,000 kb). (3) We eliminated instrumental variables with an F-statistic less than 10 to minimize potential weak instrument bias F = R^2^(n-k-1)/k(1-R^2^) (n is the sample size, k is the number of included instrumental variables, and R^2^ is the exposure variance explained by the selected SNPs).

#### Statistical analysis

The inverse variance weighted (IVW) method was employed as the main analysis, to obtain an unbiased estimate of the causal relationship between sarcopenia-related traits and OA. Furthermore, the weighted median and MR-Egger were applied as additional methods to estimate causal effects under different conditions. The weighted median could combine data from multiple genetic variants into a single causal estimate, providing a consistent estimate when at least 50% of weights are from valid IVs [38]. The MR-Egger method, which allows all SNPs with horizontal pleiotropic effects to be unbalanced or directed, was used to estimate the causal effect of exposure on the outcome.

The intercept of MR-Egger regression was used to assess horizontal pleiotropy, and *P* value > 0.05 indicated that the possibility of pleiotropy effect in causal analysis is weak [39]. In addition, two-sample MR analysis might have heterogeneity due to the differences in analytical platforms, experimental conditions, and enrolled populations, which might lead to bias in the estimation of causal effects. Then, we employed Cochran's Q test to evaluate the heterogeneity of instrumental variables [40]. If the *P* value of the test result is greater than 0.05, it was considered that there was no heterogeneity in the included instrumental variables. Moreover, we applied the Mendelian randomization pleiotropy residual sum and outlier (MR-PRESSO) method to determine horizontal pleiotropy and correct potential outliers. Finally, the leave-one-out method was used for sensitivity analysis, which sequentially removed one of the SNPs and used the remaining SNPs as instrumental variables for two-sample MR analysis to judge the degree of influence of causal association effect by a single SNP. The ‘TwoSampleMR’ package and the ‘MRPRESSO’ package in R software (version 4.1.3) were used for all MR analyses.

## Results

### Baseline characteristics of the participants

A total of 5318 participants were involved in the cross-sectional study, with the weighted characteristics of the participants as shown in Table 1. In comparison with the non-osteoarthritis group, those with OA tended to be older, female, more educated, non-Hispanic white, and higher BUN, P, TC, TG, UA, weight, WC, BMI (*P* < 0.001). Meanwhile, participants who smoked, had diabetes, hypertension, and sarcopenia were more likely to have an increased risk of OA. In addition, the values of ALM/BMI and grip strength were significantly lower in OA patients (*P* < 0.001).

**Table 1:** Baseline characteristics of the research population with and without osteoarthritis

	Osteoarthritis (*n* = 698)	Non-osteoarthritis (*n* = 4620)	*P* value
*Demographics*			
Age (years)	48.62 ± 8.80	38.84 ± 11.65	< 0.001
Gender			< 0.001
Male	279 (40.01%)	2400 (51.93%)	
Female	419 (59.99%)	2220 (48.07%)	
Race/ethnicity (%)			< 0.001
Mexican American	27 (3.82%)	477 (10.33%)	
Other race	53 (7.61%)	707 (15.31%)	
Non-Hispanic white	565 (80.90%)	2915 (63.09%)	
Non-Hispanic black	53 (7.67%)	521 (11.27%)	
Level of education (%)			< 0.001
Less than high school	81 (11.65%)	639 (13.82%)	
High school	143 (20.49%)	946 (20.48%)	
More than high school	474 (67.86%)	3035 (65.69%)	
Smoking behavior (%)			< 0.001
Smoker	388 (55.59%)	1886 (40.81%)	
Non-smoker	310 (44.41%)	2734 (59.17%)	
Alcohol consumption (%)			0.351
Drinker	368 (52.69%)	2041 (44.19%)	
Non-drinker	330 (47.31%)	2589 (55.81%)	
Hypertension (%)			< 0.001
Yes	325 (46.59%)	997 (21.59%)	
No	373(53.41%)	3623 (78.41%)	
Diabetes (%)			< 0.001
Yes	76 (10.88%)	236 (5.12%)	
No	622 (89.12%)	4384 (94.88%)	
*Laboratory indices*			
Blood urea nitrogen (mmol/L)	4.54 ± 1.62	4.29 ± 1.48	0.003
Total calcium (mmol/L)	2.36 ± 0.10	2.35 ± 0.09	0.879
Phosphorus (mmol/L)	1.25 ± 0.17	1.22 ± 0.18	0.003
Triglycerides (mmol/L)	2.17 ± 2.08	1.68 ± 1.61	< 0.001
Total cholesterol (mmol/L)	5.17 ± 1.13	4.95 ± 1.04	< 0.001
Creatinine (µmol/L)	74.21 ± 16.18	76.66 ± 25.57	0.083
Uric acid (µmol/L)	326.31 ± 83.20	317.28 ± 80.26	0.047
*Anthropometric and body composition*			
Weight (kg)	88.71 ± 20.51	82.05 ± 20.43	< 0.001
Height (cm)	169.16 ± 9.92	169.64 ± 9.56	0.373
WC (cm)	104.22 ± 15.29	97.01 ± 15.81	< 0.001
BMI (kg/m^2^)	31.10 ± 7.27	28.43 ± 6.38	< 0.001
Appendicular lean mass (kg)	22.68 ± 6.34	23.08 ± 6.33	0.112
ALM: BMI	0.74 ± 0.19	0.83 ± 0.20	< 0.001
Grip strength (kg)	37.35 ± 11.16	40.74 ± 11.25	< 0.001
Sarcopenia (%)			< 0.001
Yes	75 (10.75%)	322 (6.97%)	
No	623 (89.26%)	4298 (93.03%)	

Continuous variables were presented by mean ± standard deviation (SD), and categorical variables were presented with numbers and percentages (%). T-test was used to assess the statistical difference between the osteoarthritis group and the non-osteoarthritis group

BMI, body mass index; WC, waist circumference

### Associations between sarcopenia and the prevalence of osteoarthritis.

As presented in Table 2, sarcopenia was positively related with the prevalence of OA, and the odds ratio (OR) is 1.607 (95% CI 1.233–2.094), 1.833 (95% CI 1.393–2.413), and 1.356 (95% CI 1.003–1.832), respectively, in the model 1, model 2, and model 3. To further evaluate the relationship between sarcopenia and OA risk, we used subgroup analysis and generalized additive models. In the subgroup analyses that were stratified by gender, BMI and age, we discovered that a significant positive association between sarcopenia and OA risk in males (OR 1.592, 95% CI 1.219 to 2.079, *P* < 0.001), females (OR 1.833, 95% CI 1.393 to 2.413, *P* < 0.001), and the age (46–59 years) group (OR 1.929, 95% CI 1.359 to 2.739, *P* < 0.001) in model 3. In addition, sarcopenia was consistently associated with OA risk in participants with BMI between 18.5 and 24.9 kg/m^2^ after adjusting for different covariates.

**Table 2:** Associations between sarcopenia and the prevalence of osteoarthritis

	Model 1, OR (95% CI, *P*)	Model 2, OR (95% CI, *P*)	Model 3, OR (95% CI, *P*)
No-sarcopenia	Reference	Reference	Reference
Sarcopenia	1.607 (1.233, 2.094) < 0.001	1.833 (1.393, 2.413) < 0.001	1.356 (1.003, 1.832) 0.048
Subgroup analysis stratified by gender			
Male			
No-sarcopenia	Reference	Reference	Reference
Sarcopenia	1.699 (1.120, 2.578) 0.013	1.524 (1.079, 2.154) 0.017	1.592 (1.219, 2.079) < 0.001
Female			
No-sarcopenia	Reference	Reference	Reference
Sarcopenia	1.848 (1.204, 2.834) 0.005	1.798 (1.257, 2.571) 0.001	1.833 (1.393, 2.413) < 0.001
Subgroup analysis stratified by BMI			
BMI < 18.5			
No-sarcopenia	Reference	Reference	Reference
Sarcopenia	1.212 (0.923, 1.591) 0.166	1.380 (1.042, 1.827) 0.025	1.152 (0.850, 1.561) 0.361
BMI 18.5–24.9			
No-sarcopenia	Reference	Reference	Reference
Sarcopenia	1.910 (1.337, 2.482) 0.029	1.833 (1.393, 2.413) < 0.001	1.356 (1.003, 1.832) 0.048
BMI 25–29.9			
No-sarcopenia	Reference	Reference	Reference
Sarcopenia	1.345 (0.732, 2.470) 0.339	1.640 (0.876, 3.069) 0.122	1.597 (0.815, 3.131) 0.173
BMI ≥ 30			
No-sarcopenia	Reference	Reference	Reference
Sarcopenia	1.378 (1.031, 1.841) 0.030	1.439 (1.043, 1.985) 0.027	1.181 (0.836, 1.667) 0.345
Subgroup analysis stratified by age			
Age 20–45			
No-sarcopenia	Reference	Reference	Reference
Sarcopenia	0.702 (0.438, 1.125) 0.142	0.756 (0.468, 1.223) 0.255	1.490 (0.881, 2.519) 0.137
Age 46–59			
No-sarcopenia	Reference	Reference	Reference
Sarcopenia	1.225 (0.897, 1.673) 0.202	1.361 (0.985, 1.881) 0.062	1.929 (1.359, 2.739) < 0.001

Logistic regression models:

Model 1: no covariates were adjusted

Model 2 was adjusted for demographic factors, including gender, age, and race

Model 3 was adjusted for gender, age, race, education, smoking behavior, alcohol consumption, hypertension, diabetes, blood urea nitrogen, total calcium, phosphorus, triglycerides, total cholesterol, creatinine, and uric acid

### Associations between grip strength and the prevalence of osteoarthritis.

We explored the association between grip strength and OA risk by quartile stratification of grip strength (Table 3). We observed that significant associations between grip strength and OA risk across all the quadrant categories and the risk of OA decreased with increasing extent of grip strength. Besides, the trend remained significant among different quartile groups (*P* for trend < 0.001). The results of subgroup analyses according to gender, BMI and age are presented in Table 3, and there were similar negative associations between grip strength and OA risk in male, female, and the age (46–59 years) group. Furthermore, in the BMI subgroup analysis, grip strength and the prevalence of OA still showed substantial association in the four BMI groups after completely adjusting for interference factors.

**Table 3:** Associations between grip strength and the prevalence of osteoarthritis

	Model 1, OR (95% CI, *P*)	Model 2, OR (95% CI, *P*)	Model 3, OR (95% CI, *P*)
Grip strength (kg)	0.972 (0.964, 0.979) < 0.001	0.969 (0.957, 0.981) < 0.001	0.978 (0.965, 0.991) < 0.001
Grip strength (quartile)			
Q1	Reference	Reference	Reference
Q2	0.644 (0.522, 0794) < 0.001	0.577 (0.463, 0.718) < 0.001	0.652 (0.516, 0.825) < 0.001
Q3	0.512 (0.410, 0.638) < 0.001	0.536 (0.396, 0.726) < 0.001	0.567 (0.409, 0.786) < 0.001
Q4	0.442 (0.351, 0.555) < 0.001	0.437 (0.304, 0.627) < 0.001	0.517 (0.352, 0.761) < 0.001
*P* for trend	< 0.001	< 0.001	< 0.001
Subgroup analysis stratified by gender			
Male	0.980 (0.973, 0.987) 0.027	0.980 (0.965, 0.995) 0.009	0.979 (0.970, 0.987) 0.021
Female	0.971 (0.953, 0.990) 0.003	0.949 (0.930, 0.968) < 0.001	0.961 (0.941, 0.982) < 0.001
Subgroup analysis stratified by BMI			
BMI < 18.5	0.968 (0.961, 0.976) < 0.001	0.964 (0.956, 0.971) < 0.001	0.965 (0.956, 0.973) < 0.001
BMI 18.5–24.9	0.975 (0.957, 0.993) 0.006	0.968 (0.950, 0.987) < 0.001	0.963 (0.943, 0.983) < 0.001
BMI 25–29.9	0.969 (0.956, 0.983) < 0.001	0.964 (0.950, 0.978) < 0.001	0.962 (0.947, 0.977) < 0.001
BMI ≥ 30	0.965 (0.955, 0.976) < 0.001	0.962 (0.952, 0.973) < 0.001	0.967 (0.955, 0.978) < 0.001
Subgroup analysis stratified by age			
Age 20–45	0.993 (0.981, 1.005) 0.256	1.011 (0.990, 1.031) 0.313	0.996 (0.975, 1.018) 0.738
Age 46–59	0.967 (0.957, 0.977) < 0.001	0.973 (0.957, 0.990) 0.002	0.972 (0.954, 0.989) 0.002

Logistic regression models:

Model 1: no covariates were adjusted

Model 2 was adjusted for demographic factors, including gender, age, and race

Model 3 was adjusted for gender, age, race, education, smoking behavior, alcohol consumption, hypertension, diabetes, blood urea nitrogen, total calcium, phosphorus, triglycerides, total cholesterol, creatinine, and uric acid

### MR analyses—different MR estimation methods for assessing the causal effect of sarcopenia-related traits on osteoarthritis

**Fig. 2:**
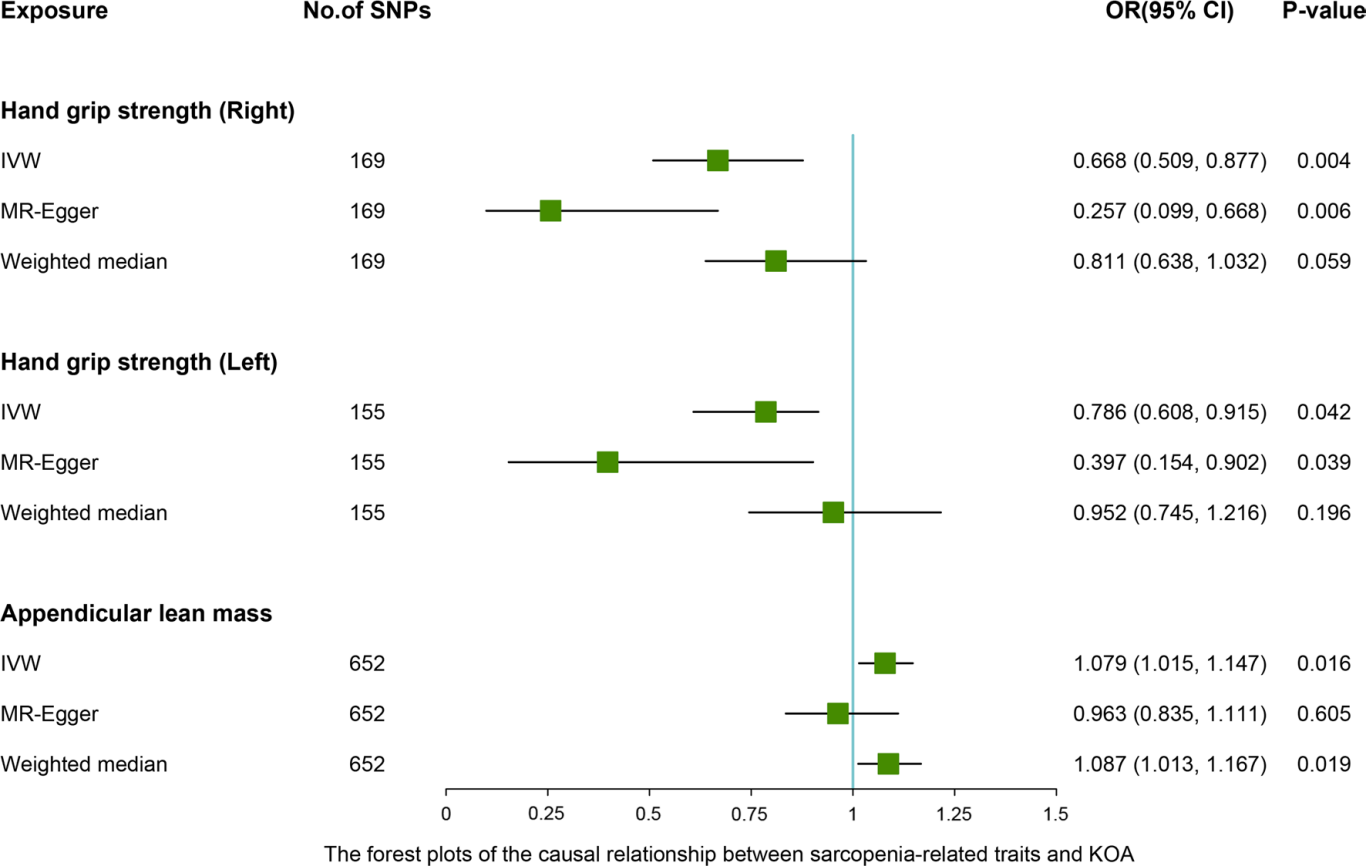
Associations between levels of sarcopenia-related traits (right-hand grip, left-hand grip, and appendicular lean mass) and knee osteoarthritis (KOA). The forest plot contains the effects, 95% CI, and *P* values of all the examined associations in analyses

**Fig. 3:**
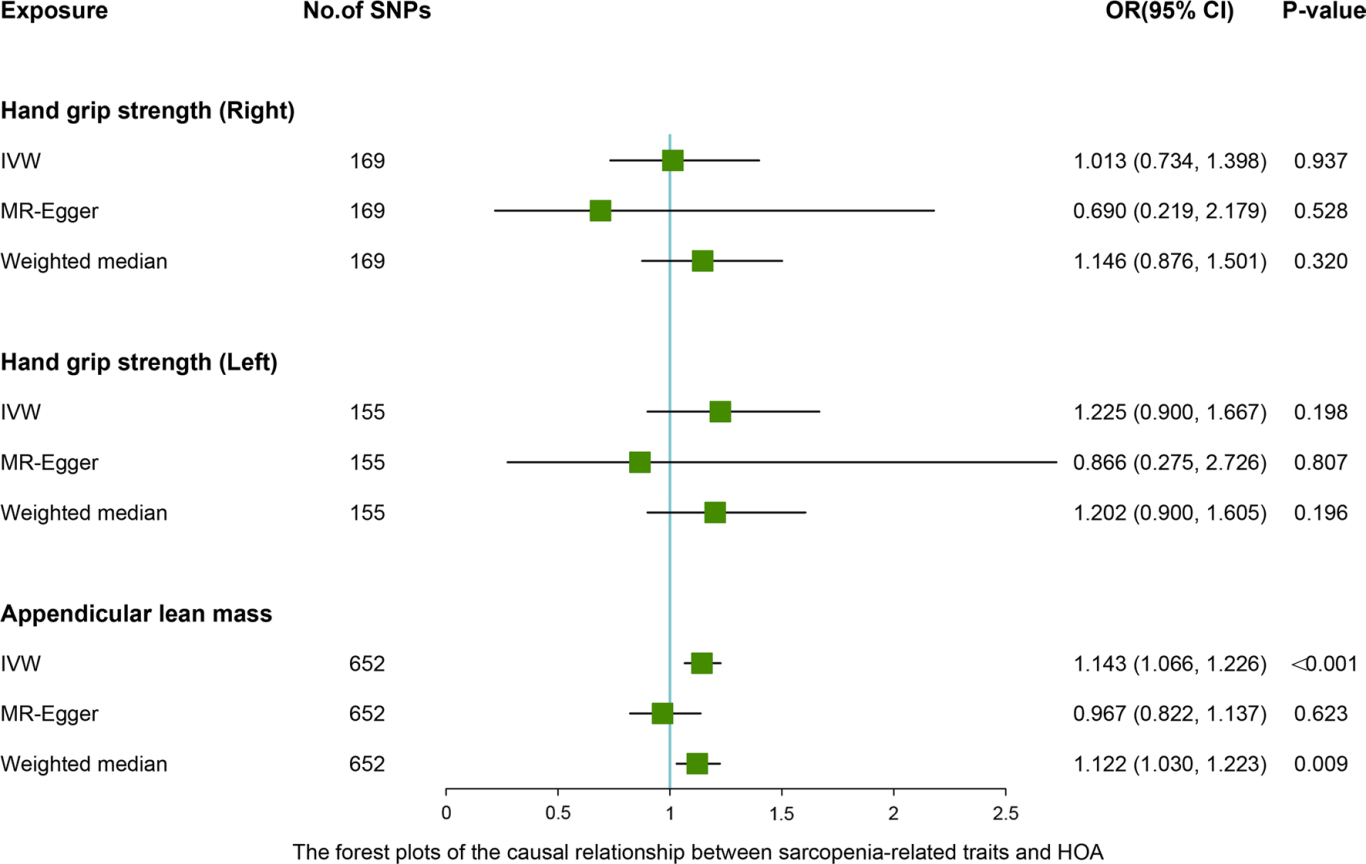
Associations between levels of sarcopenia-related traits (right-hand grip, left-hand grip, and appendicular lean mass) and hip osteoarthritis (HOA). The forest plot contains the effects, 95% CI, and *P* values of all the examined associations in analyses

**Fig. 4:**
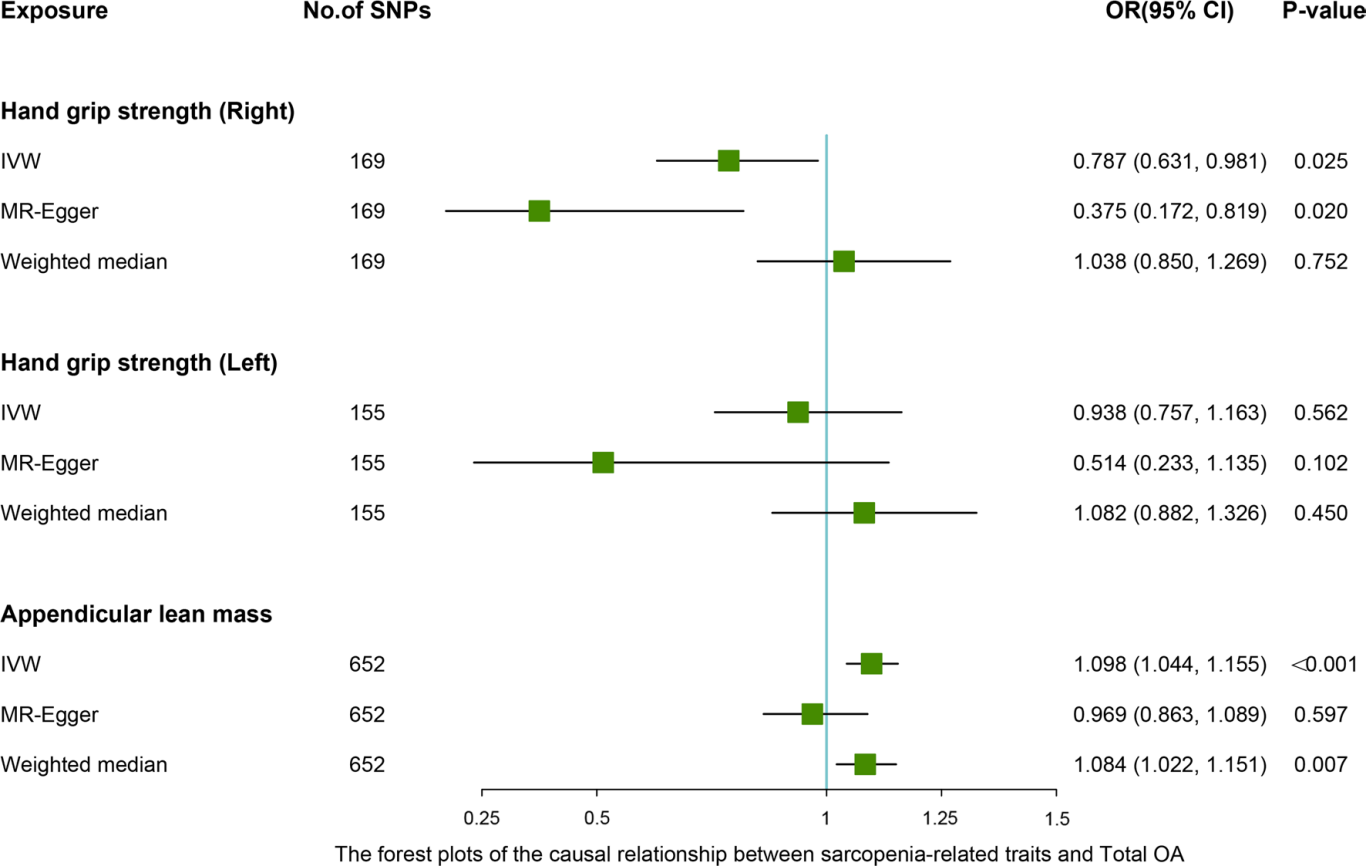
Associations between levels of sarcopenia-related traits (right-hand grip, left-hand grip, and appendicular lean mass) and total osteoarthritis (TOA). The forest plot contains the effects, 95% CI, and *P* values of all the examined associations in analyses

After removing the linkage disequilibrium effect for sarcopenia-related traits, we first identified 176, 157, and 690 significant genome-wide and independently inherited SNPs associated with right grip, left grip, and ALM, respectively. Detailed information on the SNPs associated with sarcopenia-related traits that were ultimately used for MR analysis is shown in Additional file 1: Table S2-S4. The IVW results suggested that genetically predicted right grip was linked to a decreased risk of KOA (OR 0.668; 95% CI -0.715 to -0.157; *P* = 0.002), total OA (OR 0.787; 95% CI 0.631 to 0.981; *P* = 0.025). Similar causal estimates for KOA and total OA were obtained from the MR-Egger (KOA: OR 0.257, 95% CI 0.099 to 0.668, *P* = 0.006), (total OA: OR 0.375, 95% CI 0.172 to 0.819, *P* = 0.020) (Table 4). However, no statistically significant associations were observed between right grip and HOA. As shown in Table 4, genetically increased left grip was negatively correlated with KOA (IVW: OR 0.786, 95% CI 0.608 to 0.915, *P* = 0.042), which indicated that a 1-SD increase in left grip was associated with a 21.4% decrease in the risk of KOA. And this significant finding was also supported by the MR-Egger method (OR 0.397, 95% CI 0.154 to 0.902, *P* = 0.039). In addition, according to the IVW analysis results, we discovered a direct association between ALM with KOA (OR 1.079, 95% CI 1.015 to 1.147, *P* = 0.016), HOA (OR 1.143, 95% CI 1.066 to 1.226, *P* < 0.001), total OA (OR 1.098, 95% CI 1.044 to 1.155, *P* < 0.001), and weighted median obtained a similar pattern of effect (Table 4). However, little evidence was provided for the causality between ALM and KOA (OR 0.963, 95% CI 0.835 to 1.111, *P* = 0.605), HOA (OR 0.967, 95% CI 0.822 to 1.137, *P* = 0.623), and total OA (OR 0.969, 95% CI 0.863 to 1.089, *P* = 0.597) in the MR-Egger method, and MR-Egger estimates were directionally inconsistent with the IVW and weighted median results. The forest plots of the causal relationship between sarcopenia-related traits and KOA, HOA, total OA are shown in Figs. 2, 3 and 4.

**Table 4:** MR estimates for the causal effect of sarcopenia-related traits on osteoarthritis

Exposure	Outcome	Number of SNPs	IVW OR (95% CI)	*P *value	MR-Egger OR (95% CI)	*P *value	Weighted median OR (95% CI)	*P *value
Right grip	KOA	169	0.668 (0.509, 0.877)	0.004	0.257 (0.099, 0.668)	0.006	0.811 (0.638, 1.032)	0.059
	HOA	169	1.013 (0.734, 1.398)	0.937	0.690 (0.219, 2.179)	0.528	1.146 (0.876, 1.501)	0.320
	Total OA	169	0.787 (0.631, 0.981)	0.025	0.375 (0.172, 0.819)	0.020	1.038 (0.850, 1.269)	0.752
Left grip	KOA	155	0.786 (0.608, 0.915)	0.042	0.397 (0.154, 0.902)	0.039	0.952 (0.745, 1.216)	0.196
	HOA	155	1.225 (0.900, 1.667)	0.198	0.866 (0.275, 2.726)	0.807	1.202 (0.900, 1.605)	0.213
	Total OA	155	0.938 (0.757, 1.163)	0.562	0.514 (0.233, 1.135)	0.102	1.082 (0.882, 1.326)	0.450
Appendicular lean mass	KOA	652	1.079 (1.015, 1.147)	0.016	0.963 (0.835, 1.111)	0.605	1.087 (1.013, 1.167)	0.019
	HOA	652	1.143 (1.066, 1.226)	< 0.001	0.967 (0.822, 1.137)	0.623	1.122 (1.030, 1.223)	0.009
	Total OA	652	1.098 (1.044, 1.155)	< 0.001	0.969 (0.863, 1.089)	0.597	1.084 (1.022, 1.151)	0.007

IVW, inverse-variance weighted; MR-Egger, Mendelian randomization Egger; SNP, single nucleotide polymorphism; 95% CI, 95% confidence interval; KOA, knee osteoarthritis; HOA, hip osteoarthritis;

### Sensitivity analysis

To assess the stability and objectivity of the above results, we performed a series of sensitivity analyses, including Cochran’s Q test, MR-Egger regression, and MR-PRESSO for right grip, left grip, and ALM with three or more genetic variants (Table 5). The Cochran’s Q test revealed no heterogeneity for the causal effect of right grip, left grip, and ALM on KOA, HOA, and total OA (*P* > 0.05) (Table 5). All *P* values of the MR-Egger intercept tests were > 0.05, which indicated a low chance of heterogeneity. Furthermore, Egger intercepts also did not detect any pleiotropy, suggesting that no pleiotropic bias was introduced to MR estimates in the context of heterogeneity (no horizontal pleiotropy existed). In addition, we also did not discover any outliers through the MR-PRESSO global test.

**Table 5:** Sensitivity analysis of the Mendelian randomization (MR) analysis results of sarcopenia-related traits and BMD of KOA, HOA, Total OA

Exposure	Outcome	Cochran Q statistic	Heterogeneity * P *value	MR-Egger Intercept	Intercept * P *value	MR-PRESSO Global test * P *value
Right grip	KOA	63.716	0.370	0.013	0.033	0.204
	HOA	58.078	0.109	0.005	0.531	0.157
	Total OA	63.860	0.430	0.010	0.439	0.387
Left grip	KOA	48.186	0.561	0.009	0.115	0.183
	HOA	44.973	0.357	0.005	0.495	0.808
	Total OA	50.834	0.483	0.008	0.102	0.188
Appendicular lean mass	KOA	1664.708	0.565	0.003	0.836	0.217
	HOA	1367.043	0.490	0.004	0.248	0.213
	Total OA	1670.698	0.145	0.003	0.197	0.228

MR-Egger, Mendelian randomization Egger; SNP, single nucleotide polymorphism; 95% CI, 95% confidence interval; KOA, knee osteoarthritis; HOA, hip osteoarthritis

## Discussion

In the study, we used the cross-sectional analysis and MR analysis to explore whether there were independent associations between sarcopenia and OA. The results of the observational study revealed that sarcopenia was positively associated with the risk of OA. In addition, grip strength was found to be significantly negatively linked to the prevalence of OA. Then, we used GWAS data and conducted a two-sample MR analysis to further explore the causal relationship between sarcopenia-related traits and OA at different skeleton sites. Our MR results confirmed that right grip, left grip were inversely and causally associated with the risk of KOA, but ALM was not significantly related to OA risk. To our knowledge, this was the first investigation to genetically estimate the predicted effect of sarcopenia-related traits on OA based on publicly available GWAS data, which may provide new ideas for future treatment of OA.

Bone, cartilage, and muscle are closely related and their function declines with aging [41–43]. According to previous clinical studies, muscles and joints are functionally interdependent, with muscles controlling joint movement and maintaining joint stability [44, 45]. In recent years, researchers have increasingly focused on the role of muscle atrophy in OA [46, 47]. In our study, we discovered a significant positive correlation between sarcopenia and OA, and this correlation remained significant after adjusting for multiple variables. Similarly, a large observational cohort study observed an increased risk of KOA in individuals with lower quadriceps muscle strength [48]. Two additional studies also suggested that muscle strength exercise training improve quadriceps muscle strength and performance to relieve knee pain and improves knee function in individuals with KOA [49, 50]. According to a large cross-sectional study conducted in Germany, the prevalence of sarcopenia among elder women over 70 with OA was 1.60 times higher than those without OA [51]. In addition, an experimental study observed that quadriceps muscle atrophy resulted in joint instability and would subsequently trigger the subchondral bone abnormal change in rats with quadriceps muscle atrophy, which indicates the quality and strength of the lower limb muscles play an important role in the development of OA [52].

In the current study, we also found that decreased strength, measured as grip strength, was linked to a higher risk of OA. Additionally, our MR analysis demonstrated that genetically proxied higher right grip and left grip were causally correlated with KOA, which indicates a significant role of grip strength in the development of OA. Grip strength was considered a proxy measure of overall muscle strength and was the most commonly used indicator of muscle condition in large epidemic studies [53, 54]. In a cross-sectional investigation, lower grip strength was linked to the severity of joint space narrowing, osteophytes, and subchondral cysts in the hand [55]. A Framingham study found that participants with symptomatic hand OA had lower grip strength compared with individuals without symptomatic hand OA after accounting for age, gender, occupation, BMI, and physical activity [56]. Furthermore, our results indicated that muscle strength had different effects on OA at different skeletal sites. In our MR analysis, we did not find a significant correlation between grip strength and HOA, or total OA. This might be partially due to the different composition of bones (cortical and trabecular bone and the significant regional variation in bone microstructure) from different skeletal sites, which is determined by genetic factors [57].

In addition, some information in the subgroup analysis should also be noted. First of all, in the subgroup analysis by gender, we discovered that sarcopenia and grip strength were positively and negatively related to the risk of OA in both males and females, respectively. The relationship remained significant after adjusting for multiple confounders, indicating this relationship is independent of gender. However, we observed that sarcopenia was associated with increased risk of OA only among individuals with normal BMI (BMI: 18.5–24.9 kg/m^2^), but not in the other groups. As is well-known, obesity was an important risk factor for the occurrence and progression of sarcopenia and OA [58, 59]. According to a large longitudinal cohort of 1653 subjects, we demonstrated that body composition-based obesity and sarcopenic obesity contribute to KOA [60]. Another cross-sectional study found an increase in KOA risk with increasing quartiles of BMI and fat mass but no association was found with lower extremity muscle mass [61]. Therefore, we considered that body composition and fat mass may be significant influencing factors in the study of the association of sarcopenia with OA. In the future, it would be necessary to analyze subgroups of sarcopenia, obesity, and sarcopenia to better understand how these disorders are related.

Some mechanistic studies seem to provide preliminary explanations for the relationship between sarcopenia and OA. There was increasing evidence that inflammation is capable of triggering or facilitating the onset of important age-related diseases such as sarcopenia, osteoporosis, and OA [62, 63]. The imbalance in the inflammatory system due to increased pro-inflammatory cytokines induces the dysfunction of chondrocyte synthesis and breakdown in muscles and articular cartilage, which eventually leads to muscle loss and cartilage destruction [64, 65]. Inflammatory factors such as tumor necrosis factor-alpha (TNF-α), C-reactive protein (CRP), and interleukin-6 (IL-6) could increase the expression of atrogin-1 and MuRF-1 by the ubiquitin–proteasome system (UPS), thereby increasing muscle breakdown and decreasing synthesis [66, 67]. Meanwhile, TNF-α and IL-6 are involved in the pathogenesis of OA by activating the nuclear factor kappa-B (NF-κB) pathway in chondrocytes and synoviocytes, leading to chondrocyte apoptosis, cartilage extracellular matrix (ECM) degradation, and subchondral bone dysfunction [68–70]. Additionally, there was evidence that insulin resistance is associated with the occurrence of sarcopenia and OA. Several studies have suggested that insulin resistance mediated the activation of caspase-3 by signaling through the PI3K/Akt pathway, inducing skeletal muscle loss by reducing protein synthesis with increased protein breakdown in muscle [71]. Insulin resistance resulted in the obstruction of the combination of insulin and insulin receptors, which weakens the role of insulin in blocking inflammation-causing substances and the ability to inhibit catabolism [72], thereby increasing synovial inflammation and contributing to OA development. Besides, insulin resistance limited pro-anabolic effects of insulin and enhances free fatty acids (FFA) production that facilitates chondrocyte apoptosis and induces OA pathogenesis via TLR-4 [73].

Our study has some strengths. To begin with, we used the generalizability of NHANES data, which included representative non-institutionalized Americans, which enabled our findings to be presented as generalizability. Second, we combined the cross-sectional study with Mendelian randomization and obtained consistent results to ensure the robustness and objectivity of the study results. Last but not least, multiple logistic regression, stratified analysis, sensitivity analyses, and heterogeneity analysis were used to provide reliable evidence of an independent association between grip strength and OA risk. Nevertheless, the present study also had some limitations. Firstly, data on OA were collected by questionnaire in the cross-sectional study and might inevitably be influenced to recall bias. Secondly, due to the lack of demographic data in the MR study (e.g., gender, age and race), we were unable to perform further subgroup analyses to obtain more specific effect relationships. Finally, our study population was mostly European–American, and the findings should be confirmed in other races or populations.

## Conclusion

The present study demonstrates a positive correlation between sarcopenia and prevalence of OA, and this relationship is independent of gender. Furthermore, the MR study provides evidence for the negative causal relationship between grip strength and OA. Still, a large amount of studies are needed to further elucidate the role of sarcopenia in the occurrence and progression of OA in the future.

## Supplementary Information

**Additional file 1.** Detailed characteristics of GWAS associated with exposures and outcomes in the study. Scatter plot, funnel plot, and leave-one-out analysis of the causal effect of sarcopenia on OA risk.

## Data Availability

The survey data are publicly available on the Internet for data users and researchers throughout the world http://www.cdc.gov/nchs/nhanes/.
