# Supplementary material for: Osteoarthritis and sarcopenia-related traits: the cross-sectional study from NHANES 2011–2014 and Mendelian randomization study
Source: J Orthop Surg Res. 2023 Jul 15;18:502. doi: 10.1186/s13018-023-03960-w (PMC10349510; doi:10.1186/s13018-023-03960-w)

Supplementary Material

**Supplementary Table S1.** Detailed characteristics of GWAS associated with exposures and outcomes in the study.

| **Traits** | **Consortium** | **Year** | **Population** | **Sample size** | **PMID** |
| --- | --- | --- | --- | --- | --- |
| **Exposure** |  |  |  |  |  |
| Hand grip strength (right) | MRC-IEU | 2018 | European | 461,089 | 25826379 |
| Hand grip strength (left) | MRC-IEU | 2018 | European | 461,026 | 25826379 |
| Appendicular lean mass | UK Biobank | 2020 | European | 450,243 | 33097823 |
| **Outcome** |  |  |  |  |  |
| Knee osteoarthritis | Genetics of Osteoarthritis (GO) Consortium | 2021 | European | 826,690 | 34822786 |
| Hip osteoarthritis | Genetics of Osteoarthritis (GO) Consortium | 2021 | European | 826,690 | 34822786 |
| Osteoarthritis of the hip or knee | Genetics of Osteoarthritis (GO) Consortium | 2021 | European | 826,690 | 34822786 |

Note: GWAS, genome-wide association study

**Supplementary Table S2.** Summary information on the SNPs used as genetic instruments for Hand grip strength (right).

| SNP | Chr | Pos | Effect allele | Other allele | EAF | Beta | SE | *P* val | F statistic |
| --- | --- | --- | --- | --- | --- | --- | --- | --- | --- |
| rs58670122 | 1 | 22492613 | G | A | 0.143 | -0.013 | 0.002 | 7.40E-10 | 38 |
| rs10798876 | 1 | 32074514 | G | C | 0.552 | 0.009 | 0.001 | 4.30E-09 | 34 |
| rs10798483 | 1 | 176799143 | A | G | 0.547 | 0.015 | 0.001 | 2.80E-22 | 94 |
| rs6693965 | 1 | 10378416 | T | G | 0.128 | -0.016 | 0.002 | 3.20E-13 | 53 |
| rs4927015 | 1 | 54072471 | A | G | 0.583 | 0.013 | 0.002 | 5.00E-18 | 75 |
| rs1952256 | 1 | 184035116 | G | A | 0.345 | 0.010 | 0.002 | 4.10E-10 | 39 |
| rs35304341 | 1 | 200971049 | A | G | 0.089 | -0.014 | 0.003 | 4.00E-08 | 30 |
| rs823130 | 1 | 205714372 | T | C | 0.433 | -0.012 | 0.002 | 1.70E-16 | 68 |
| rs56144131 | 1 | 208977656 | C | T | 0.149 | -0.013 | 0.002 | 3.80E-10 | 39 |
| rs7549184 | 1 | 215412906 | A | G | 0.787 | 0.011 | 0.002 | 5.80E-09 | 34 |
| rs10799428 | 1 | 227798565 | T | C | 0.187 | -0.014 | 0.002 | 4.60E-14 | 57 |
| rs12562146 | 1 | 86247267 | A | T | 0.145 | 0.012 | 0.002 | 2.00E-08 | 31 |
| rs2147461 | 1 | 191051627 | C | T | 0.117 | 0.013 | 0.002 | 6.10E-09 | 34 |
| rs4121165 | 1 | 78276977 | A | G | 0.211 | -0.012 | 0.002 | 4.80E-11 | 43 |
| rs6693567 | 1 | 150510660 | T | C | 0.733 | -0.010 | 0.002 | 6.30E-09 | 34 |
| rs1892425 | 1 | 41744821 | A | G | 0.239 | 0.011 | 0.002 | 1.10E-10 | 42 |
| rs150330307 | 1 | 160160801 | C | T | 0.032 | -0.033 | 0.004 | 1.20E-14 | 60 |
| rs1550115 | 2 | 25041620 | T | C | 0.749 | 0.015 | 0.002 | 4.50E-19 | 80 |
| rs1442883 | 2 | 59970660 | A | C | 0.253 | -0.011 | 0.002 | 5.80E-10 | 38 |
| rs6711390 | 2 | 135629439 | T | C | 0.371 | 0.013 | 0.002 | 4.00E-18 | 75 |
| rs35833641 | 2 | 179462494 | G | A | 0.312 | 0.009 | 0.002 | 8.50E-09 | 33 |
| rs1840753 | 2 | 199060177 | T | C | 0.063 | 0.018 | 0.003 | 1.40E-08 | 32 |
| rs1047891 | 2 | 211540507 | A | C | 0.316 | 0.010 | 0.002 | 1.30E-09 | 37 |
| rs2894602 | 2 | 227249802 | G | A | 0.766 | 0.010 | 0.002 | 9.90E-09 | 33 |
| rs7565148 | 2 | 44188396 | G | T | 0.501 | -0.010 | 0.001 | 3.60E-12 | 48 |
| rs7576964 | 2 | 48601448 | T | G | 0.342 | 0.010 | 0.002 | 5.30E-10 | 39 |
| rs34030812 | 2 | 144248905 | C | T | 0.367 | -0.009 | 0.002 | 3.30E-09 | 35 |
| rs7575451 | 2 | 152352843 | G | C | 0.650 | -0.011 | 0.002 | 9.30E-12 | 46 |
| rs12616285 | 2 | 169156907 | G | T | 0.149 | 0.012 | 0.002 | 5.40E-09 | 34 |
| rs6715064 | 2 | 220041928 | T | C | 0.310 | -0.009 | 0.002 | 9.60E-09 | 33 |
| rs1641457 | 2 | 40421990 | G | T | 0.223 | 0.013 | 0.002 | 2.40E-13 | 54 |
| rs3771498 | 2 | 70720070 | T | C | 0.514 | 0.014 | 0.001 | 1.90E-21 | 90 |
| rs12052508 | 2 | 72757843 | T | C | 0.879 | -0.013 | 0.002 | 4.70E-09 | 34 |
| rs10193039 | 2 | 99692373 | T | A | 0.281 | -0.010 | 0.002 | 4.10E-10 | 39 |
| rs2194747 | 2 | 218126181 | G | A | 0.707 | 0.010 | 0.002 | 1.90E-09 | 36 |
| rs6792762 | 3 | 38574491 | A | G | 0.420 | -0.009 | 0.002 | 1.80E-09 | 36 |
| rs7652177 | 3 | 171969077 | G | C | 0.505 | 0.009 | 0.001 | 7.90E-09 | 33 |
| rs2194411 | 3 | 185548663 | A | G | 0.128 | 0.014 | 0.002 | 2.10E-10 | 40 |
| rs1440152 | 3 | 98489915 | G | C | 0.445 | 0.008 | 0.001 | 3.50E-08 | 30 |
| rs2362972 | 3 | 158163272 | A | C | 0.578 | -0.009 | 0.002 | 1.30E-08 | 32 |
| rs62234790 | 3 | 13750642 | A | C | 0.246 | 0.011 | 0.002 | 8.80E-10 | 38 |
| rs35701422 | 3 | 85575775 | C | T | 0.626 | -0.009 | 0.002 | 1.50E-08 | 32 |
| rs2341184 | 3 | 196930781 | C | T | 0.272 | 0.010 | 0.002 | 1.70E-09 | 36 |
| rs35457492 | 3 | 70194564 | C | A | 0.495 | 0.008 | 0.001 | 2.20E-08 | 31 |
| rs9757079 | 3 | 53155158 | T | C | 0.318 | 0.010 | 0.002 | 8.40E-10 | 38 |
| rs71298370 | 3 | 71164965 | A | G | 0.086 | 0.017 | 0.003 | 8.90E-10 | 38 |
| rs9853018 | 3 | 141101961 | T | C | 0.443 | 0.010 | 0.001 | 8.80E-12 | 47 |
| rs34587452 | 4 | 1009900 | C | G | 0.215 | -0.011 | 0.002 | 9.10E-10 | 38 |
| rs7657558 | 4 | 30648636 | G | T | 0.720 | 0.011 | 0.002 | 1.10E-10 | 42 |
| rs114924396 | 4 | 119755621 | G | A | 0.054 | -0.019 | 0.003 | 8.20E-09 | 33 |
| rs13146142 | 4 | 17931318 | C | T | 0.159 | -0.021 | 0.002 | 1.40E-24 | 105 |
| rs13106087 | 4 | 145566864 | C | T | 0.830 | 0.013 | 0.002 | 6.20E-11 | 43 |
| rs997850 | 4 | 154838434 | C | G | 0.605 | -0.009 | 0.002 | 3.70E-09 | 35 |
| rs13107325 | 4 | 103188709 | T | C | 0.075 | -0.028 | 0.003 | 2.10E-22 | 95 |
| rs13169333 | 5 | 153064994 | C | T | 0.260 | 0.009 | 0.002 | 4.30E-08 | 30 |
| rs75457267 | 5 | 102658770 | T | C | 0.051 | -0.019 | 0.003 | 3.40E-08 | 30 |
| rs12522139 | 5 | 122699812 | G | T | 0.171 | -0.011 | 0.002 | 6.80E-09 | 34 |
| rs6882168 | 5 | 39402647 | T | C | 0.337 | -0.009 | 0.002 | 7.90E-09 | 33 |
| rs13356200 | 5 | 67820946 | G | T | 0.394 | -0.009 | 0.002 | 1.80E-09 | 36 |
| rs6870324 | 5 | 141787317 | G | C | 0.270 | -0.010 | 0.002 | 3.00E-09 | 35 |
| rs13355365 | 5 | 154942606 | T | C | 0.380 | -0.008 | 0.002 | 3.40E-08 | 30 |
| rs4868110 | 5 | 171164168 | T | A | 0.323 | -0.010 | 0.002 | 1.00E-09 | 37 |
| rs2431112 | 5 | 103931707 | A | G | 0.441 | -0.011 | 0.001 | 8.60E-14 | 56 |
| rs2631360 | 5 | 131707429 | A | G | 0.519 | -0.011 | 0.001 | 5.40E-14 | 57 |
| rs2322754 | 6 | 81046299 | A | G | 0.832 | -0.012 | 0.002 | 3.80E-09 | 35 |
| rs9322822 | 6 | 105369598 | T | C | 0.320 | 0.011 | 0.002 | 3.50E-12 | 48 |
| rs9388051 | 6 | 122577108 | A | G | 0.186 | 0.011 | 0.002 | 2.70E-08 | 31 |
| rs7451021 | 6 | 130381246 | C | T | 0.689 | -0.016 | 0.002 | 7.70E-23 | 97 |
| rs113835839 | 6 | 13784625 | T | C | 0.248 | -0.010 | 0.002 | 1.10E-08 | 33 |
| rs11243202 | 6 | 7719065 | C | T | 0.486 | 0.012 | 0.001 | 6.50E-15 | 61 |
| rs645144 | 6 | 141180780 | C | T | 0.330 | -0.009 | 0.002 | 4.30E-08 | 30 |
| rs721101 | 6 | 155632844 | C | T | 0.271 | 0.009 | 0.002 | 1.60E-08 | 32 |
| rs77485342 | 6 | 30842866 | T | C | 0.018 | 0.035 | 0.006 | 2.60E-10 | 40 |
| rs9267806 | 6 | 32110886 | A | G | 0.256 | -0.017 | 0.002 | 7.70E-23 | 97 |
| rs185320691 | 6 | 32490292 | C | G | 0.104 | -0.020 | 0.003 | 5.60E-14 | 57 |
| rs113315602 | 6 | 32574575 | C | A | 0.096 | -0.021 | 0.003 | 1.40E-15 | 64 |
| rs1125 | 6 | 149979416 | A | G | 0.337 | -0.010 | 0.002 | 1.90E-10 | 41 |
| rs9396861 | 6 | 18404133 | A | C | 0.599 | -0.010 | 0.002 | 5.70E-10 | 38 |
| rs35175534 | 6 | 32530029 | C | A | 0.140 | -0.019 | 0.002 | 4.20E-16 | 66 |
| rs1885690 | 6 | 109672998 | A | C | 0.410 | -0.008 | 0.002 | 2.80E-08 | 31 |
| rs852520 | 7 | 5695267 | A | C | 0.662 | -0.009 | 0.002 | 1.50E-08 | 32 |
| rs4549685 | 7 | 39326478 | T | C | 0.330 | 0.010 | 0.002 | 8.10E-10 | 38 |
| rs112330055 | 7 | 23109316 | A | G | 0.063 | 0.018 | 0.003 | 1.70E-08 | 32 |
| rs7790322 | 7 | 2830498 | T | C | 0.416 | -0.009 | 0.002 | 1.20E-08 | 32 |
| rs6962338 | 7 | 69160985 | G | A | 0.044 | -0.020 | 0.004 | 2.00E-08 | 32 |
| rs6977081 | 7 | 150542515 | T | G | 0.334 | 0.013 | 0.002 | 5.70E-16 | 66 |
| rs2389763 | 7 | 17307847 | C | T | 0.592 | -0.008 | 0.002 | 3.80E-08 | 30 |
| rs2717351 | 7 | 19019880 | G | A | 0.212 | 0.013 | 0.002 | 3.40E-12 | 48 |
| rs9639938 | 7 | 46262729 | G | C | 0.541 | 0.009 | 0.001 | 5.20E-09 | 34 |
| rs10278546 | 7 | 100516003 | C | A | 0.195 | 0.011 | 0.002 | 1.10E-08 | 33 |
| rs4730984 | 7 | 120655676 | T | G | 0.240 | 0.010 | 0.002 | 1.80E-09 | 36 |
| rs4737446 | 8 | 57665019 | T | G | 0.695 | 0.010 | 0.002 | 2.40E-10 | 40 |
| rs62509875 | 8 | 110360944 | G | A | 0.171 | -0.013 | 0.002 | 2.40E-11 | 45 |
| rs1486925 | 8 | 78827617 | C | T | 0.315 | -0.010 | 0.002 | 2.90E-09 | 35 |
| rs6473015 | 8 | 78178485 | C | A | 0.286 | 0.010 | 0.002 | 5.50E-09 | 34 |
| rs7871404 | 9 | 99262296 | G | A | 0.189 | 0.012 | 0.002 | 3.40E-10 | 39 |
| rs116922558 | 9 | 118802375 | G | A | 0.040 | -0.025 | 0.004 | 1.80E-10 | 41 |
| rs2208562 | 9 | 119344528 | T | C | 0.610 | -0.012 | 0.002 | 3.10E-14 | 58 |
| rs7034200 | 9 | 4289050 | A | C | 0.480 | 0.009 | 0.001 | 5.30E-09 | 34 |
| rs113851275 | 9 | 98297220 | A | G | 0.108 | 0.013 | 0.002 | 4.30E-08 | 30 |
| rs11998884 | 9 | 33684436 | T | C | 0.062 | 0.017 | 0.003 | 2.80E-08 | 31 |
| rs600038 | 9 | 136151806 | C | T | 0.207 | -0.010 | 0.002 | 1.50E-08 | 32 |
| rs10761411 | 9 | 136973826 | T | C | 0.812 | -0.011 | 0.002 | 1.30E-08 | 32 |
| rs72820369 | 10 | 81251539 | T | A | 0.118 | 0.016 | 0.002 | 4.40E-12 | 48 |
| rs4751671 | 10 | 116138744 | A | G | 0.531 | 0.008 | 0.002 | 4.10E-08 | 30 |
| rs4752689 | 10 | 124131176 | A | G | 0.584 | 0.009 | 0.002 | 6.40E-09 | 34 |
| rs12412806 | 10 | 24860913 | A | G | 0.294 | -0.009 | 0.002 | 3.00E-08 | 31 |
| rs2273555 | 10 | 104127171 | A | G | 0.606 | 0.011 | 0.002 | 4.20E-13 | 53 |
| rs4962700 | 10 | 126479989 | G | C | 0.302 | 0.009 | 0.002 | 1.20E-08 | 32 |
| rs1556659 | 10 | 130834698 | T | C | 0.382 | 0.018 | 0.002 | 3.80E-30 | 130 |
| rs12763284 | 10 | 104508202 | G | A | 0.466 | 0.010 | 0.001 | 1.20E-10 | 42 |
| rs11022513 | 11 | 12840986 | T | C | 0.569 | -0.009 | 0.002 | 1.10E-09 | 37 |
| rs11039348 | 11 | 47728617 | A | G | 0.348 | -0.010 | 0.002 | 3.90E-10 | 39 |
| rs2244621 | 11 | 64026219 | T | C | 0.144 | 0.012 | 0.002 | 4.50E-08 | 30 |
| rs61389091 | 11 | 74427921 | T | C | 0.042 | 0.022 | 0.004 | 3.60E-09 | 35 |
| rs34845616 | 11 | 133792644 | A | G | 0.246 | 0.010 | 0.002 | 1.70E-08 | 32 |
| rs12790261 | 11 | 66988048 | A | C | 0.082 | -0.026 | 0.003 | 2.00E-22 | 95 |
| rs72977282 | 11 | 74300441 | A | T | 0.414 | -0.017 | 0.002 | 1.20E-28 | 123 |
| rs6592737 | 11 | 77322619 | T | A | 0.372 | -0.009 | 0.002 | 2.00E-09 | 36 |
| rs10770125 | 11 | 2169014 | G | A | 0.477 | 0.008 | 0.001 | 1.40E-08 | 32 |
| rs1635527 | 12 | 48396364 | C | G | 0.547 | 0.010 | 0.001 | 6.80E-11 | 43 |
| rs76895963 | 12 | 4384844 | G | T | 0.021 | 0.036 | 0.006 | 4.10E-10 | 39 |
| rs7301953 | 12 | 124405871 | A | G | 0.312 | -0.012 | 0.002 | 6.40E-13 | 52 |
| rs10846071 | 12 | 15016236 | T | C | 0.394 | -0.016 | 0.002 | 6.50E-25 | 106 |
| rs12823922 | 12 | 24186697 | G | A | 0.222 | -0.011 | 0.002 | 1.60E-10 | 41 |
| rs10784502 | 12 | 66343810 | T | C | 0.512 | -0.011 | 0.001 | 7.10E-14 | 56 |
| rs7963801 | 12 | 79685226 | C | T | 0.572 | -0.011 | 0.002 | 8.40E-14 | 56 |
| rs12316046 | 12 | 15054415 | G | A | 0.378 | -0.016 | 0.002 | 3.80E-26 | 112 |
| rs4768725 | 12 | 46848478 | C | T | 0.700 | 0.009 | 0.002 | 2.30E-08 | 31 |
| rs7953280 | 12 | 94136009 | C | G | 0.507 | -0.009 | 0.001 | 1.80E-09 | 36 |
| rs3118914 | 13 | 51116901 | T | G | 0.215 | -0.019 | 0.002 | 5.90E-27 | 116 |
| rs2296316 | 14 | 65520246 | C | T | 0.464 | -0.008 | 0.002 | 4.60E-08 | 30 |
| rs12889267 | 14 | 21542766 | G | A | 0.167 | -0.012 | 0.002 | 6.70E-10 | 38 |
| rs935728 | 14 | 80957923 | T | C | 0.328 | 0.010 | 0.002 | 1.80E-09 | 36 |
| rs7148603 | 14 | 36683779 | A | G | 0.359 | 0.009 | 0.002 | 4.70E-09 | 34 |
| rs10483727 | 14 | 61072875 | C | T | 0.610 | -0.009 | 0.002 | 3.30E-09 | 35 |
| rs9652468 | 15 | 56823913 | A | G | 0.249 | -0.013 | 0.002 | 3.30E-13 | 53 |
| rs2871865 | 15 | 99194896 | G | C | 0.116 | -0.024 | 0.002 | 1.80E-24 | 104 |
| rs2165241 | 15 | 74222202 | C | T | 0.509 | 0.012 | 0.001 | 1.90E-16 | 68 |
| rs4553566 | 15 | 58336319 | C | T | 0.453 | -0.009 | 0.001 | 4.60E-10 | 39 |
| rs12914702 | 15 | 96887277 | A | G | 0.300 | 0.011 | 0.002 | 1.90E-10 | 41 |
| rs12101479 | 15 | 74248548 | C | G | 0.237 | -0.011 | 0.002 | 1.50E-09 | 37 |
| rs12899474 | 15 | 77391603 | T | C | 0.108 | -0.015 | 0.002 | 5.40E-10 | 39 |
| rs246181 | 16 | 14392641 | T | C | 0.373 | 0.010 | 0.002 | 3.70E-10 | 39 |
| rs11642954 | 16 | 24824248 | A | G | 0.195 | -0.013 | 0.002 | 2.20E-12 | 49 |
| rs7196917 | 16 | 69896527 | G | A | 0.430 | -0.011 | 0.002 | 1.90E-12 | 50 |
| rs8055199 | 16 | 84867404 | A | G | 0.660 | -0.009 | 0.002 | 1.20E-08 | 32 |
| rs7206195 | 16 | 2145280 | T | C | 0.180 | -0.015 | 0.002 | 2.80E-15 | 62 |
| rs248831 | 16 | 11281218 | A | G | 0.265 | 0.010 | 0.002 | 8.40E-09 | 33 |
| rs62037412 | 16 | 28917746 | A | G | 0.357 | 0.009 | 0.002 | 1.90E-09 | 36 |
| rs4785574 | 16 | 89568875 | G | A | 0.555 | -0.010 | 0.001 | 4.50E-12 | 48 |
| rs3848369 | 16 | 415078 | T | C | 0.387 | -0.010 | 0.002 | 4.70E-10 | 39 |
| rs76749769 | 16 | 3291408 | T | C | 0.091 | 0.014 | 0.003 | 2.20E-08 | 31 |
| rs4784329 | 16 | 53910261 | C | A | 0.426 | -0.013 | 0.002 | 9.00E-19 | 78 |
| rs7214252 | 17 | 27486673 | A | G | 0.211 | -0.010 | 0.002 | 2.00E-08 | 31 |
| rs2854152 | 17 | 61986027 | G | A | 0.678 | 0.011 | 0.002 | 6.00E-12 | 47 |
| rs1043515 | 17 | 36922196 | G | A | 0.566 | 0.014 | 0.001 | 2.80E-20 | 85 |
| rs12452505 | 17 | 63556402 | G | C | 0.142 | -0.014 | 0.002 | 1.40E-11 | 46 |
| rs2587505 | 17 | 77784268 | C | T | 0.420 | -0.009 | 0.002 | 8.10E-10 | 38 |
| rs4793658 | 17 | 45878733 | C | A | 0.110 | -0.014 | 0.002 | 3.80E-08 | 30 |
| rs56074046 | 17 | 7358930 | A | G | 0.372 | -0.009 | 0.002 | 4.40E-09 | 34 |
| rs56365901 | 17 | 43960323 | G | A | 0.223 | -0.014 | 0.002 | 1.80E-15 | 63 |
| rs10520770 | 18 | 46602964 | C | T | 0.449 | 0.012 | 0.001 | 4.50E-15 | 61 |
| rs635538 | 18 | 53273614 | A | G | 0.914 | -0.022 | 0.003 | 1.50E-16 | 68 |
| rs4369779 | 18 | 20735408 | C | T | 0.789 | 0.017 | 0.002 | 3.40E-21 | 89 |
| rs34217742 | 19 | 37376830 | A | T | 0.124 | 0.015 | 0.002 | 1.40E-10 | 41 |
| rs7249 | 19 | 18391171 | T | C | 0.366 | 0.008 | 0.002 | 4.00E-08 | 30 |
| rs4802848 | 19 | 52218342 | C | G | 0.730 | 0.011 | 0.002 | 3.80E-11 | 44 |
| rs36065733 | 19 | 2163771 | G | T | 0.473 | 0.010 | 0.001 | 8.20E-11 | 42 |
| rs79723785 | 19 | 55818225 | C | T | 0.016 | -0.034 | 0.006 | 1.60E-08 | 32 |
| rs7266065 | 20 | 47531817 | A | G | 0.323 | 0.010 | 0.002 | 4.60E-10 | 39 |
| rs911642 | 20 | 13260252 | T | C | 0.376 | 0.009 | 0.002 | 2.00E-08 | 31 |
| rs6063504 | 20 | 48981014 | G | C | 0.493 | 0.009 | 0.001 | 7.80E-09 | 33 |
| rs143384 | 20 | 34025756 | G | A | 0.404 | 0.023 | 0.002 | 2.50E-52 | 232 |
| rs2226685 | 21 | 40069825 | C | T | 0.759 | 0.010 | 0.002 | 3.10E-09 | 35 |
| rs6006984 | 22 | 45714937 | C | T | 0.278 | 0.010 | 0.002 | 5.10E-10 | 39 |

Abbreviations: SNPs, single-nucleotide polymorphisms; EAF, effect allele frequency; Se, standard error.

**Supplementary Table S3.** Summary information on the SNPs used as genetic instruments for Hand grip strength (left).

| SNP | Chr | Pos | Effect allele | Other allele | EAF | Beta | SE | *P* val | F statistic |
| --- | --- | --- | --- | --- | --- | --- | --- | --- | --- |
| rs6680160 | 1 | 32072737 | G | A | 0.628 | 0.010 | 0.002 | 6.00E-11 | 43 |
| rs7516571 | 1 | 40733658 | G | A | 0.259 | 0.009 | 0.002 | 3.10E-08 | 31 |
| rs150330307 | 1 | 160160801 | C | T | 0.032 | -0.031 | 0.004 | 2.90E-13 | 53 |
| rs2800789 | 1 | 164578242 | C | A | 0.480 | 0.008 | 0.001 | 3.10E-08 | 31 |
| rs1044299 | 1 | 176811873 | T | C | 0.546 | 0.014 | 0.001 | 6.60E-21 | 88 |
| rs11121542 | 1 | 10393920 | A | G | 0.123 | -0.016 | 0.002 | 2.99985E-12 | 49 |
| rs4121165 | 1 | 78276977 | A | G | 0.211 | -0.011 | 0.002 | 3.40001E-10 | 39 |
| rs58670122 | 1 | 22492613 | G | A | 0.143 | -0.012 | 0.002 | 3.59998E-08 | 30 |
| rs10788958 | 1 | 54040670 | G | C | 0.645 | 0.014 | 0.002 | 1E-19 | 83 |
| rs4335354 | 1 | 88899964 | A | C | 0.316 | -0.009 | 0.002 | 4.60002E-09 | 34 |
| rs1884447 | 1 | 185021410 | A | G | 0.401 | 0.008 | 0.002 | 2.30001E-08 | 31 |
| rs61818100 | 1 | 190962663 | C | T | 0.117 | 0.013 | 0.002 | 6.19998E-09 | 34 |
| rs823130 | 1 | 205714372 | T | C | 0.433 | -0.011 | 0.002 | 4.40048E-14 | 57 |
| rs11204664 | 1 | 150531380 | C | T | 0.579 | -0.009 | 0.002 | 8.10009E-09 | 33 |
| rs6689375 | 1 | 227721627 | T | A | 0.185 | -0.016 | 0.002 | 5.30029E-17 | 70 |
| rs6433478 | 2 | 175241482 | C | T | 0.544 | 0.009 | 0.001 | 1.5E-09 | 36 |
| rs12473732 | 2 | 44118428 | T | C | 0.487 | 0.011 | 0.001 | 1.39991E-13 | 55 |
| rs7571789 | 2 | 70714793 | C | T | 0.523 | 0.013 | 0.001 | 2.99985E-18 | 76 |
| rs7575451 | 2 | 152352843 | G | C | 0.650 | -0.010 | 0.002 | 3.79997E-10 | 39 |
| rs1434095 | 2 | 179254330 | C | T | 0.875 | 0.014 | 0.002 | 5.30005E-10 | 39 |
| rs17630248 | 2 | 201137782 | C | T | 0.348 | -0.009 | 0.002 | 4.09996E-09 | 35 |
| rs1981612 | 2 | 199235664 | A | C | 0.456 | 0.009 | 0.002 | 0.000000001 | 37 |
| rs11125803 | 2 | 25052177 | T | C | 0.741 | 0.014 | 0.002 | 3.80014E-17 | 71 |
| rs1641457 | 2 | 40421990 | G | T | 0.223 | 0.012 | 0.002 | 1.39991E-11 | 46 |
| rs3819121 | 2 | 135622860 | C | T | 0.369 | 0.014 | 0.002 | 2.90001E-20 | 85 |
| rs10176878 | 2 | 59952274 | C | T | 0.191 | -0.013 | 0.002 | 8.60003E-12 | 47 |
| rs61286123 | 2 | 60205600 | C | T | 0.228 | -0.010 | 0.002 | 0.000000012 | 33 |
| rs34030812 | 2 | 144248905 | C | T | 0.367 | -0.010 | 0.002 | 4.10015E-11 | 44 |
| rs10205394 | 2 | 218150948 | C | G | 0.201 | -0.011 | 0.002 | 1.09999E-09 | 37 |
| rs1551042 | 3 | 85630551 | C | A | 0.647 | -0.011 | 0.002 | 7.39946E-13 | 51 |
| rs9866627 | 3 | 135522715 | A | C | 0.084 | -0.016 | 0.003 | 5.69994E-09 | 34 |
| rs112485536 | 3 | 195971019 | T | C | 0.076 | 0.016 | 0.003 | 9.40005E-09 | 33 |
| rs62253653 | 3 | 53013267 | G | A | 0.295 | 0.011 | 0.002 | 5.50047E-11 | 43 |
| rs6802071 | 3 | 38574237 | T | C | 0.435 | -0.009 | 0.002 | 3.79997E-10 | 39 |
| rs71298370 | 3 | 71164965 | A | G | 0.086 | 0.015 | 0.003 | 3.79997E-08 | 30 |
| rs13091492 | 3 | 81891476 | G | A | 0.373 | -0.008 | 0.002 | 3.29997E-08 | 31 |
| rs10934857 | 3 | 128199662 | A | G | 0.259 | 0.009 | 0.002 | 4.79999E-08 | 30 |
| rs4498020 | 3 | 13810820 | A | C | 0.724 | -0.010 | 0.002 | 4.30002E-10 | 39 |
| rs4677601 | 3 | 71368790 | G | A | 0.510 | 0.009 | 0.001 | 1.09999E-09 | 37 |
| rs2871960 | 3 | 141121814 | C | A | 0.445 | 0.012 | 0.001 | 5.50047E-16 | 66 |
| rs35609019 | 4 | 7847892 | C | G | 0.398 | 0.009 | 0.002 | 7.69999E-10 | 38 |
| rs13107325 | 4 | 103188709 | T | C | 0.075 | -0.026 | 0.003 | 1.80011E-20 | 86 |
| rs56338231 | 4 | 30867393 | G | A | 0.258 | -0.011 | 0.002 | 1.7E-10 | 41 |
| rs13146142 | 4 | 17931318 | C | T | 0.159 | -0.020 | 0.002 | 2.29985E-23 | 99 |
| rs34587452 | 4 | 1009900 | C | G | 0.215 | -0.011 | 0.002 | 3.2E-10 | 40 |
| rs13106087 | 4 | 145566864 | C | T | 0.830 | 0.012 | 0.002 | 3.89996E-09 | 35 |
| rs997850 | 4 | 154838434 | C | G | 0.605 | -0.009 | 0.002 | 6.29999E-09 | 34 |
| rs34722008 | 4 | 38659594 | A | G | 0.353 | 0.009 | 0.002 | 3.29997E-08 | 31 |
| rs2850379 | 4 | 102917419 | A | C | 0.432 | -0.008 | 0.002 | 3.29997E-08 | 31 |
| rs75497896 | 4 | 119636703 | C | T | 0.051 | -0.021 | 0.003 | 9.09997E-10 | 38 |
| rs116409670 | 5 | 37327472 | T | C | 0.080 | -0.015 | 0.003 | 2.59998E-08 | 31 |
| rs55681913 | 5 | 42687629 | C | T | 0.106 | 0.014 | 0.002 | 0.000000016 | 32 |
| rs13356200 | 5 | 67820946 | G | T | 0.394 | -0.009 | 0.002 | 9.69996E-09 | 33 |
| rs2431112 | 5 | 103931707 | A | G | 0.441 | -0.010 | 0.001 | 1.40001E-10 | 41 |
| rs2631360 | 5 | 131707429 | A | G | 0.519 | -0.011 | 0.001 | 1.9002E-13 | 54 |
| rs6882168 | 5 | 39402647 | T | C | 0.337 | -0.009 | 0.002 | 2.80001E-09 | 35 |
| rs113918482 | 5 | 161289270 | G | A | 0.223 | -0.010 | 0.002 | 0.000000015 | 32 |
| rs2974438 | 5 | 168250903 | A | G | 0.211 | -0.010 | 0.002 | 3.29997E-08 | 31 |
| rs185320691 | 6 | 32490292 | C | G | 0.104 | -0.017 | 0.003 | 5.49997E-10 | 39 |
| rs12528131 | 6 | 105389104 | G | A | 0.488 | -0.009 | 0.001 | 2.80001E-09 | 35 |
| rs9371201 | 6 | 150145001 | T | C | 0.335 | -0.009 | 0.002 | 2.99999E-09 | 35 |
| rs77485342 | 6 | 30842866 | T | C | 0.018 | 0.033 | 0.006 | 3.50002E-09 | 35 |
| rs9388769 | 6 | 130379954 | A | G | 0.673 | -0.014 | 0.002 | 5.10035E-19 | 79 |
| rs35175534 | 6 | 32530029 | C | A | 0.140 | -0.016 | 0.002 | 3.59998E-12 | 48 |
| rs113315602 | 6 | 32574575 | C | A | 0.096 | -0.018 | 0.003 | 8.60003E-12 | 47 |
| rs723588 | 6 | 81056634 | C | T | 0.143 | 0.013 | 0.002 | 1.6E-09 | 36 |
| rs11243202 | 6 | 7719065 | C | T | 0.486 | 0.010 | 0.001 | 7.00003E-11 | 43 |
| rs4713506 | 6 | 32113980 | A | G | 0.256 | -0.016 | 0.002 | 1.99986E-20 | 86 |
| rs2038760 | 6 | 2680732 | T | C | 0.171 | -0.012 | 0.002 | 6.59994E-09 | 34 |
| rs41271299 | 6 | 19839415 | T | C | 0.051 | 0.021 | 0.003 | 2.99999E-10 | 40 |
| rs9371881 | 6 | 155638213 | A | G | 0.359 | 0.009 | 0.002 | 9.59997E-10 | 37 |
| rs4621706 | 7 | 39303296 | T | C | 0.544 | -0.012 | 0.002 | 6.00067E-15 | 61 |
| rs11769549 | 7 | 23122239 | A | T | 0.062 | 0.020 | 0.003 | 4.49987E-11 | 43 |
| rs12533765 | 7 | 127699186 | G | A | 0.280 | -0.009 | 0.002 | 2.59998E-08 | 31 |
| rs16870531 | 7 | 120660682 | T | C | 0.238 | 0.011 | 0.002 | 1.29999E-10 | 41 |
| rs13227429 | 7 | 140560023 | C | T | 0.561 | -0.009 | 0.001 | 9.80009E-09 | 33 |
| rs6977081 | 7 | 150542515 | T | G | 0.334 | 0.015 | 0.002 | 1.39991E-20 | 86 |
| rs12673062 | 7 | 4710677 | A | G | 0.216 | -0.011 | 0.002 | 2.59998E-09 | 35 |
| rs73307079 | 7 | 19020024 | C | T | 0.211 | 0.011 | 0.002 | 1.40001E-09 | 37 |
| rs6962338 | 7 | 69160985 | G | A | 0.044 | -0.021 | 0.004 | 3.2E-09 | 35 |
| rs17282763 | 7 | 82520166 | C | T | 0.296 | 0.009 | 0.002 | 4.39997E-08 | 30 |
| rs821100 | 8 | 89448877 | G | A | 0.266 | -0.010 | 0.002 | 1.5E-09 | 37 |
| rs59116179 | 8 | 22603454 | T | C | 0.617 | 0.009 | 0.002 | 2.39999E-08 | 31 |
| rs4737446 | 8 | 57665019 | T | G | 0.695 | 0.010 | 0.002 | 1.2E-10 | 41 |
| rs1486925 | 8 | 78827617 | C | T | 0.315 | -0.010 | 0.002 | 6.29941E-11 | 43 |
| rs4398863 | 8 | 135695110 | C | G | 0.737 | -0.009 | 0.002 | 0.00000002 | 31 |
| rs4739739 | 8 | 81304576 | G | A | 0.415 | -0.009 | 0.002 | 1.40001E-08 | 32 |
| rs10097417 | 8 | 110361477 | G | A | 0.171 | -0.013 | 0.002 | 1.9002E-11 | 45 |
| rs7026798 | 9 | 16427378 | C | T | 0.432 | 0.008 | 0.002 | 4.60002E-08 | 30 |
| rs7856625 | 9 | 119345083 | T | C | 0.610 | -0.011 | 0.002 | 2.49977E-13 | 54 |
| rs16910750 | 9 | 99084471 | C | G | 0.160 | 0.011 | 0.002 | 3.59998E-08 | 30 |
| rs116922558 | 9 | 118802375 | G | A | 0.040 | -0.022 | 0.004 | 0.000000021 | 31 |
| rs2789514 | 9 | 129833029 | A | G | 0.868 | 0.012 | 0.002 | 4.90004E-08 | 30 |
| rs10988217 | 9 | 131888116 | G | A | 0.604 | -0.009 | 0.002 | 1.7E-09 | 36 |
| rs11002322 | 10 | 79649653 | T | G | 0.340 | -0.010 | 0.002 | 2.1E-10 | 40 |
| rs10786706 | 10 | 104500659 | T | C | 0.466 | 0.010 | 0.001 | 1.69981E-11 | 45 |
| rs35236379 | 10 | 5727292 | T | G | 0.142 | 0.012 | 0.002 | 5.80003E-09 | 34 |
| rs11003014 | 10 | 81231387 | G | A | 0.161 | 0.011 | 0.002 | 0.000000016 | 32 |
| rs4962700 | 10 | 126479989 | G | C | 0.302 | 0.009 | 0.002 | 0.000000032 | 31 |
| rs772014 | 10 | 104062494 | G | A | 0.393 | -0.011 | 0.002 | 2.70023E-12 | 49 |
| rs10821939 | 10 | 63751748 | A | G | 0.573 | -0.009 | 0.002 | 4.90004E-10 | 39 |
| rs1556659 | 10 | 130834698 | T | C | 0.382 | 0.016 | 0.002 | 2.49977E-26 | 113 |
| rs72977282 | 11 | 74300441 | A | T | 0.414 | -0.016 | 0.002 | 7.8001E-25 | 106 |
| rs4930236 | 11 | 68414000 | A | C | 0.836 | 0.012 | 0.002 | 3.79997E-09 | 35 |
| rs10831903 | 11 | 12758660 | T | C | 0.423 | 0.009 | 0.002 | 8.10009E-10 | 38 |
| rs12790261 | 11 | 66988048 | A | C | 0.082 | -0.025 | 0.003 | 1.20005E-20 | 87 |
| rs7124681 | 11 | 47529947 | A | C | 0.408 | -0.012 | 0.002 | 1E-14 | 60 |
| rs61389091 | 11 | 74427921 | T | C | 0.042 | 0.026 | 0.004 | 2.39994E-12 | 49 |
| rs34845616 | 11 | 133792644 | A | G | 0.246 | 0.011 | 0.002 | 4E-10 | 39 |
| rs76895963 | 12 | 4384844 | G | T | 0.021 | 0.036 | 0.006 | 3.89996E-10 | 39 |
| rs10846071 | 12 | 15016236 | T | C | 0.394 | -0.017 | 0.002 | 5.30029E-28 | 120 |
| rs11168357 | 12 | 48412138 | A | G | 0.246 | -0.010 | 0.002 | 0.000000025 | 31 |
| rs4575361 | 12 | 124410529 | T | A | 0.312 | -0.011 | 0.002 | 1.59993E-11 | 45 |
| rs12316046 | 12 | 15054415 | G | A | 0.378 | -0.017 | 0.002 | 5.00035E-30 | 130 |
| rs7970350 | 12 | 66360164 | T | C | 0.494 | -0.010 | 0.001 | 8.60003E-12 | 47 |
| rs7963801 | 12 | 79685226 | C | T | 0.572 | -0.010 | 0.002 | 4.60045E-12 | 48 |
| rs11111267 | 12 | 102811239 | G | A | 0.181 | 0.011 | 0.002 | 1.89998E-08 | 32 |
| rs3118903 | 13 | 51099577 | A | G | 0.220 | -0.017 | 0.002 | 2.70023E-22 | 94 |
| rs56060323 | 13 | 60473485 | T | C | 0.315 | 0.009 | 0.002 | 0.000000015 | 32 |
| rs12889267 | 14 | 21542766 | G | A | 0.167 | -0.014 | 0.002 | 4.70002E-12 | 48 |
| rs7148603 | 14 | 36683779 | A | G | 0.359 | 0.010 | 0.002 | 1.6E-09 | 36 |
| rs2359239 | 14 | 75326771 | T | C | 0.392 | -0.009 | 0.002 | 6.90001E-09 | 34 |
| rs10144445 | 14 | 39695362 | G | C | 0.350 | -0.009 | 0.002 | 1.89998E-09 | 36 |
| rs28542042 | 15 | 74213357 | T | C | 0.309 | 0.011 | 0.002 | 1.20005E-11 | 46 |
| rs12906830 | 15 | 56963503 | C | T | 0.601 | 0.011 | 0.002 | 9.30037E-13 | 51 |
| rs3959716 | 15 | 73106615 | G | C | 0.567 | -0.008 | 0.002 | 3.09999E-08 | 31 |
| rs17466480 | 15 | 77390870 | G | A | 0.387 | -0.012 | 0.002 | 9.79941E-15 | 60 |
| rs12914702 | 15 | 96887277 | A | G | 0.300 | 0.011 | 0.002 | 9.30037E-11 | 42 |
| rs7176095 | 15 | 74886411 | G | A | 0.128 | -0.013 | 0.002 | 1.7E-09 | 36 |
| rs2871865 | 15 | 99194896 | G | C | 0.116 | -0.022 | 0.002 | 5.00035E-21 | 89 |
| rs13337177 | 16 | 2175323 | T | G | 0.181 | -0.014 | 0.002 | 1.59993E-13 | 54 |
| rs11642954 | 16 | 24824248 | A | G | 0.195 | -0.012 | 0.002 | 4.49997E-10 | 39 |
| rs217181 | 16 | 72114002 | T | C | 0.193 | 0.012 | 0.002 | 2.1E-10 | 40 |
| rs9944324 | 16 | 80929342 | G | A | 0.457 | -0.009 | 0.002 | 0.000000012 | 32 |
| rs7197751 | 16 | 84940033 | T | G | 0.363 | -0.009 | 0.002 | 1.40001E-09 | 37 |
| rs7196917 | 16 | 69896527 | G | A | 0.430 | -0.012 | 0.002 | 5.40008E-15 | 61 |
| rs181766 | 16 | 14394878 | C | T | 0.322 | 0.010 | 0.002 | 1.89998E-09 | 36 |
| rs3814877 | 16 | 30042677 | T | G | 0.402 | 0.011 | 0.002 | 3.40017E-12 | 48 |
| rs11076004 | 16 | 53913930 | A | G | 0.418 | -0.012 | 0.002 | 2.09991E-14 | 58 |
| rs113434679 | 17 | 44126765 | A | C | 0.200 | -0.015 | 0.002 | 1.9002E-15 | 63 |
| rs755547 | 17 | 43011908 | A | G | 0.189 | 0.017 | 0.002 | 3.19963E-18 | 76 |
| rs2532111 | 17 | 62017421 | G | A | 0.640 | 0.010 | 0.002 | 4.10015E-11 | 44 |
| rs999493 | 17 | 46625519 | A | G | 0.622 | 0.013 | 0.002 | 6.20012E-17 | 70 |
| rs2587505 | 17 | 77784268 | C | T | 0.420 | -0.009 | 0.002 | 2.30001E-09 | 36 |
| rs635538 | 18 | 53273614 | A | G | 0.914 | -0.022 | 0.003 | 3.80014E-16 | 66 |
| rs4308051 | 18 | 20735461 | G | T | 0.789 | 0.016 | 0.002 | 1.39991E-18 | 77 |
| rs62081464 | 18 | 35142133 | T | C | 0.227 | -0.010 | 0.002 | 2.80001E-08 | 31 |
| rs143002906 | 18 | 12992162 | T | C | 0.028 | 0.026 | 0.005 | 9.80009E-09 | 33 |
| rs35054365 | 18 | 46612306 | A | T | 0.438 | 0.013 | 0.001 | 1.20005E-17 | 73 |
| rs10403906 | 19 | 37376756 | A | G | 0.476 | -0.010 | 0.001 | 1.50003E-11 | 46 |
| rs11669079 | 19 | 52219938 | T | A | 0.705 | 0.011 | 0.002 | 1.9002E-11 | 45 |
| rs8101782 | 19 | 12507992 | C | A | 0.703 | 0.010 | 0.002 | 2.90001E-08 | 31 |
| rs8108461 | 19 | 2186757 | C | T | 0.573 | 0.010 | 0.002 | 2.59998E-10 | 40 |
| rs143384 | 20 | 34025756 | G | A | 0.404 | 0.021 | 0.002 | 1.50003E-43 | 192 |
| rs4811040 | 20 | 48968438 | G | C | 0.277 | -0.009 | 0.002 | 2.99999E-08 | 31 |
| rs9611273 | 22 | 40534466 | T | C | 0.253 | 0.011 | 0.002 | 4.49997E-10 | 39 |
| rs6006984 | 22 | 45714937 | C | T | 0.278 | 0.010 | 0.002 | 2.59998E-09 | 35 |

Abbreviations: SNPs, single-nucleotide polymorphisms; EAF, effect allele frequency; Se, standard error.

**Supplementary Table S4.** Summary information on the SNPs used as genetic instruments for Appendicular lean mass.

| SNP | Chr | Pos | Effect allele | Other allele | EAF | Beta | SE | *P* val | F statistic |
| --- | --- | --- | --- | --- | --- | --- | --- | --- | --- |
| rs200439 | 1 | 6716083 | C | A | 0.221 | -0.013 | 0.002 | 1.51E-08 | 31 |
| rs2807339 | 1 | 22578063 | C | T | 0.759 | 0.016 | 0.002 | 1.24E-13 | 54 |
| rs150188352 | 1 | 56632354 | CAAT | C | 0.194 | 0.031 | 0.002 | 8.43E-38 | 162 |
| rs60804050 | 1 | 118870373 | A | G | 0.256 | -0.022 | 0.002 | 5.01E-24 | 107 |
| rs2025808 | 1 | 184161757 | A | C | 0.254 | 0.012 | 0.002 | 1.72E-08 | 31 |
| rs6693481 | 1 | 203766395 | C | T | 0.695 | -0.014 | 0.002 | 2.21E-12 | 51 |
| rs61827272 | 1 | 203810763 | C | T | 0.275 | 0.014 | 0.002 | 7.89E-12 | 47 |
| rs12724708 | 1 | 219620569 | T | A | 0.357 | 0.024 | 0.002 | 1.66E-35 | 148 |
| rs3033487 | 1 | 227731073 | TCC | T | 0.812 | 0.015 | 0.002 | 1.93E-10 | 41 |
| rs11260623 | 1 | 1781456 | T | G | 0.509 | 0.012 | 0.002 | 4.86E-10 | 38 |
| rs301807 | 1 | 8484823 | G | A | 0.582 | -0.014 | 0.002 | 2.53E-14 | 57 |
| rs212526 | 1 | 21584941 | C | T | 0.601 | 0.021 | 0.002 | 3.84E-29 | 127 |
| rs7522400 | 1 | 36613380 | G | A | 0.768 | 0.013 | 0.002 | 5.62E-09 | 34 |
| rs670318 | 1 | 63727542 | C | T | 0.952 | 0.041 | 0.004 | 2.52E-21 | 88 |
| rs2025609 | 1 | 67422990 | G | C | 0.851 | 0.019 | 0.003 | 2.00E-12 | 51 |
| rs7543202 | 1 | 73872885 | G | A | 0.623 | 0.013 | 0.002 | 2.86E-11 | 46 |
| rs11590254 | 1 | 92316573 | T | A | 0.311 | 0.019 | 0.002 | 4.34E-20 | 86 |
| rs34654458 | 1 | 150546790 | ATTT | A | 0.578 | -0.019 | 0.002 | 5.72E-24 | 101 |
| rs4644481 | 1 | 155130900 | T | C | 0.433 | -0.011 | 0.002 | 3.54E-09 | 35 |
| rs7367519 | 1 | 204479176 | C | T | 0.685 | 0.016 | 0.002 | 4.68E-16 | 67 |
| rs951366 | 1 | 205685352 | C | T | 0.393 | 0.021 | 0.002 | 9.15E-27 | 116 |
| rs7418410 | 1 | 10236402 | T | C | 0.409 | 0.016 | 0.002 | 5.64E-16 | 67 |
| rs7543136 | 1 | 22472451 | T | C | 0.721 | -0.021 | 0.002 | 9.96E-24 | 100 |
| rs4360494 | 1 | 38455891 | C | G | 0.554 | -0.020 | 0.002 | 7.88E-26 | 109 |
| rs55717234 | 1 | 150999863 | G | A | 0.573 | 0.012 | 0.002 | 1.34E-10 | 41 |
| rs11580040 | 1 | 155198222 | G | A | 0.080 | 0.033 | 0.004 | 6.76E-21 | 86 |
| rs2209098 | 1 | 172167226 | C | T | 0.311 | 0.024 | 0.002 | 1.73E-32 | 144 |
| rs17278379 | 1 | 172381284 | C | T | 0.124 | 0.023 | 0.003 | 2.40E-15 | 61 |
| rs66579625 | 1 | 212201175 | CT | C | 0.584 | 0.018 | 0.002 | 8.91E-21 | 88 |
| rs4655345 | 1 | 214608704 | G | A | 0.397 | -0.025 | 0.002 | 5.81E-38 | 168 |
| rs1797070 | 1 | 218630201 | A | G | 0.268 | 0.022 | 0.002 | 4.93E-25 | 109 |
| rs12563442 | 1 | 19786695 | C | T | 0.267 | 0.012 | 0.002 | 9.93E-09 | 34 |
| rs80295797 | 1 | 23341690 | T | C | 0.327 | -0.020 | 0.002 | 3.83E-23 | 98 |
| rs4274112 | 1 | 26746199 | G | A | 0.373 | -0.022 | 0.002 | 2.47E-28 | 118 |
| rs113107560 | 1 | 36747842 | G | T | 0.412 | -0.019 | 0.002 | 6.99E-23 | 100 |
| rs11210892 | 1 | 44100084 | A | G | 0.675 | 0.012 | 0.002 | 3.57E-09 | 35 |
| rs12074850 | 1 | 51248316 | G | A | 0.090 | 0.039 | 0.003 | 2.72E-33 | 142 |
| rs1514134 | 1 | 56116513 | C | T | 0.385 | -0.011 | 0.002 | 3.57E-09 | 36 |
| rs34517439 | 1 | 78450517 | A | C | 0.122 | 0.042 | 0.003 | 5.80E-48 | 211 |
| rs10922475 | 1 | 89142142 | A | C | 0.540 | 0.016 | 0.002 | 2.17E-17 | 70 |
| rs3768495 | 1 | 109935325 | T | C | 0.717 | -0.018 | 0.002 | 1.07E-17 | 72 |
| rs28736838 | 1 | 120148713 | T | C | 0.302 | -0.012 | 0.002 | 1.07E-08 | 34 |
| rs200091076 | 1 | 154598598 | GT | G | 0.646 | -0.012 | 0.002 | 6.92E-09 | 34 |
| rs905938 | 1 | 154991389 | C | T | 0.265 | 0.039 | 0.002 | 8.43E-77 | 352 |
| rs6675858 | 1 | 224559936 | T | C | 0.214 | -0.014 | 0.002 | 2.40E-09 | 35 |
| rs2789365 | 1 | 235515534 | T | C | 0.481 | -0.015 | 0.002 | 1.14E-14 | 58 |
| rs377599 | 1 | 2164699 | T | C | 0.383 | 0.022 | 0.002 | 3.50E-29 | 130 |
| rs11121615 | 1 | 10825577 | T | C | 0.689 | -0.020 | 0.002 | 3.32E-23 | 102 |
| rs2791654 | 1 | 11129317 | A | G | 0.706 | -0.024 | 0.002 | 1.21E-28 | 118 |
| rs6425817 | 1 | 33873034 | G | A | 0.672 | 0.016 | 0.002 | 2.98E-15 | 62 |
| rs2885697 | 1 | 41544279 | T | G | 0.665 | -0.032 | 0.002 | 9.21E-60 | 261 |
| rs4847378 | 1 | 93324634 | T | G | 0.612 | 0.014 | 0.002 | 1.64E-12 | 51 |
| rs1405227 | 1 | 98873390 | A | G | 0.317 | 0.013 | 0.002 | 1.57E-10 | 42 |
| rs1325596 | 1 | 176794066 | A | G | 0.548 | 0.029 | 0.002 | 2.77E-52 | 228 |
| rs200348453 | 1 | 182970296 | AG | A | 0.474 | 0.018 | 0.002 | 7.88E-21 | 90 |
| rs234640 | 1 | 184867830 | T | C | 0.514 | -0.013 | 0.002 | 3.87E-12 | 48 |
| rs1005723 | 1 | 243646251 | T | C | 0.191 | 0.016 | 0.002 | 1.79E-11 | 45 |
| rs10171272 | 2 | 25946636 | A | C | 0.304 | 0.014 | 0.002 | 2.87E-11 | 46 |
| rs17681189 | 2 | 65976175 | A | C | 0.423 | -0.013 | 0.002 | 5.82E-12 | 48 |
| rs76517946 | 2 | 68354936 | A | C | 0.081 | -0.037 | 0.004 | 1.69E-26 | 111 |
| rs867529 | 2 | 88913273 | C | G | 0.280 | 0.018 | 0.002 | 1.00E-18 | 77 |
| rs6543146 | 2 | 103096695 | G | T | 0.558 | 0.015 | 0.002 | 4.19E-16 | 66 |
| rs6738207 | 2 | 105989716 | A | G | 0.401 | 0.013 | 0.002 | 4.14E-11 | 45 |
| rs71414738 | 2 | 127876242 | T | C | 0.176 | 0.015 | 0.003 | 1.00E-09 | 36 |
| rs2390669 | 2 | 169091942 | C | A | 0.129 | 0.017 | 0.003 | 7.00E-10 | 39 |
| rs13430869 | 2 | 218146818 | T | G | 0.743 | 0.027 | 0.002 | 6.37E-37 | 168 |
| rs17408561 | 2 | 225474277 | G | A | 0.391 | 0.012 | 0.002 | 6.00E-10 | 38 |
| rs17246129 | 2 | 227259964 | A | G | 0.304 | 0.025 | 0.002 | 1.27E-35 | 161 |
| rs2305141 | 2 | 233684402 | G | A | 0.596 | 0.018 | 0.002 | 1.07E-21 | 93 |
| rs2971857 | 2 | 234369487 | A | G | 0.575 | -0.012 | 0.002 | 3.70E-10 | 39 |
| rs10203320 | 2 | 9771620 | C | T | 0.329 | 0.014 | 0.002 | 7.86E-12 | 48 |
| rs144627572 | 2 | 20583907 | A | G | 0.033 | 0.044 | 0.005 | 1.30E-16 | 69 |
| rs1260326 | 2 | 27730940 | C | T | 0.605 | 0.032 | 0.002 | 6.16E-64 | 289 |
| rs650508 | 2 | 45880122 | C | G | 0.301 | -0.013 | 0.002 | 1.86E-10 | 42 |
| rs60142646 | 2 | 46532856 | TC | T | 0.948 | -0.024 | 0.004 | 2.49E-08 | 32 |
| rs75022676 | 2 | 60293216 | A | G | 0.208 | -0.016 | 0.002 | 2.84E-12 | 50 |
| rs199647708 | 2 | 125352195 | A | G | 0.397 | 0.011 | 0.002 | 3.43E-09 | 36 |
| rs17400325 | 2 | 178565913 | C | T | 0.042 | 0.035 | 0.005 | 2.10E-13 | 54 |
| rs1035583 | 2 | 207326937 | A | G | 0.618 | 0.015 | 0.002 | 1.99E-14 | 61 |
| rs7598430 | 2 | 219193963 | T | C | 0.505 | -0.016 | 0.002 | 1.37E-17 | 71 |
| rs10202701 | 2 | 232328681 | T | C | 0.542 | 0.023 | 0.002 | 3.11E-33 | 143 |
| rs10205141 | 2 | 11313340 | G | A | 0.048 | 0.024 | 0.004 | 4.71E-08 | 30 |
| rs10203386 | 2 | 25136866 | A | T | 0.452 | -0.024 | 0.002 | 1.75E-36 | 157 |
| rs10202845 | 2 | 42575820 | G | A | 0.113 | -0.029 | 0.003 | 5.35E-22 | 92 |
| rs67716382 | 2 | 46890317 | C | G | 0.221 | 0.023 | 0.002 | 1.65E-23 | 97 |
| rs2717008 | 2 | 58149158 | C | T | 0.384 | -0.013 | 0.002 | 4.99E-11 | 45 |
| rs4852257 | 2 | 71678520 | G | T | 0.576 | -0.023 | 0.002 | 6.21E-34 | 148 |
| rs201570119 | 2 | 112256320 | C | T | 0.210 | 0.019 | 0.002 | 5.48E-17 | 71 |
| rs35223841 | 2 | 144053000 | GAA | G | 0.389 | 0.011 | 0.002 | 4.25E-08 | 31 |
| rs55852614 | 2 | 172416869 | C | T | 0.247 | -0.039 | 0.002 | 3.29E-73 | 319 |
| rs3063063 | 2 | 183231361 | TGAA | T | 0.686 | -0.018 | 0.002 | 2.57E-19 | 83 |
| rs144343497 | 2 | 191588534 | TA | T | 0.216 | -0.013 | 0.002 | 5.06E-09 | 34 |
| rs700677 | 2 | 198702424 | A | C | 0.351 | 0.017 | 0.002 | 1.13E-18 | 75 |
| rs12997625 | 2 | 202970250 | T | C | 0.527 | -0.017 | 0.002 | 1.50E-19 | 80 |
| rs3116194 | 2 | 233061266 | A | T | 0.098 | -0.030 | 0.003 | 8.29E-21 | 85 |
| rs7570235 | 2 | 242491353 | C | T | 0.591 | -0.017 | 0.002 | 2.08E-18 | 78 |
| rs7563362 | 2 | 620297 | G | A | 0.857 | 0.035 | 0.003 | 3.27E-39 | 170 |
| rs6721191 | 2 | 10190115 | G | A | 0.578 | -0.014 | 0.002 | 3.17E-14 | 57 |
| rs12713004 | 2 | 23896049 | G | A | 0.725 | 0.037 | 0.002 | 2.40E-68 | 305 |
| rs6739278 | 2 | 44401055 | C | T | 0.808 | -0.021 | 0.002 | 1.27E-18 | 77 |
| rs2347603 | 2 | 47297426 | A | T | 0.742 | -0.018 | 0.002 | 5.65E-17 | 68 |
| rs702886 | 2 | 65753310 | G | A | 0.351 | 0.012 | 0.002 | 1.11E-09 | 36 |
| rs55980611 | 2 | 74771027 | A | C | 0.146 | 0.016 | 0.003 | 4.22E-09 | 34 |
| rs14976 | 2 | 85818886 | T | C | 0.306 | 0.014 | 0.002 | 1.43E-12 | 52 |
| rs72809820 | 2 | 97360079 | T | C | 0.324 | -0.011 | 0.002 | 3.13E-08 | 31 |
| rs9636364 | 2 | 111992435 | A | G | 0.543 | 0.011 | 0.002 | 5.09E-09 | 34 |
| rs10864899 | 2 | 112929481 | G | A | 0.564 | -0.011 | 0.002 | 3.85E-09 | 35 |
| rs488621 | 2 | 169707552 | G | A | 0.469 | 0.019 | 0.002 | 2.86E-24 | 101 |
| rs2138374 | 2 | 190014317 | C | T | 0.696 | -0.015 | 0.002 | 2.79E-13 | 56 |
| rs10221831 | 2 | 202107829 | T | C | 0.032 | 0.030 | 0.005 | 1.80E-08 | 32 |
| rs17773965 | 2 | 217631338 | T | C | 0.141 | -0.016 | 0.003 | 1.51E-09 | 36 |
| rs62106258 | 2 | 417167 | C | T | 0.049 | -0.050 | 0.004 | 6.45E-31 | 131 |
| rs3769598 | 2 | 32679732 | G | A | 0.144 | 0.017 | 0.003 | 1.32E-10 | 40 |
| rs202098543 | 2 | 55576869 | AT | A | 0.581 | 0.013 | 0.002 | 1.56E-11 | 44 |
| rs59985551 | 2 | 56106928 | T | C | 0.226 | -0.031 | 0.002 | 2.43E-44 | 202 |
| rs62143873 | 2 | 72035050 | A | G | 0.503 | -0.012 | 0.002 | 1.19E-09 | 37 |
| rs12616192 | 2 | 121568931 | A | G | 0.068 | -0.026 | 0.004 | 6.83E-12 | 47 |
| rs61397287 | 2 | 144223279 | T | A | 0.075 | 0.024 | 0.004 | 4.45E-11 | 43 |
| rs13391980 | 2 | 165504841 | A | G | 0.120 | -0.023 | 0.003 | 7.60E-15 | 60 |
| rs34788019 | 2 | 201192126 | CA | C | 0.543 | -0.014 | 0.002 | 2.84E-13 | 54 |
| rs1047891 | 2 | 211540507 | A | C | 0.316 | 0.023 | 0.002 | 5.70E-31 | 136 |
| rs1478575 | 2 | 218278555 | A | T | 0.684 | 0.031 | 0.002 | 5.20E-54 | 243 |
| rs11684531 | 2 | 219835489 | G | A | 0.133 | -0.017 | 0.003 | 4.17E-10 | 38 |
| rs1899040 | 2 | 223901896 | T | C | 0.796 | 0.015 | 0.002 | 9.04E-11 | 44 |
| rs2270894 | 3 | 9975386 | G | C | 0.203 | -0.033 | 0.002 | 1.25E-42 | 191 |
| rs113671109 | 3 | 12620885 | C | T | 0.220 | -0.015 | 0.002 | 4.23E-11 | 43 |
| rs6789000 | 3 | 25188002 | T | G | 0.645 | 0.012 | 0.002 | 1.61E-09 | 37 |
| rs4504126 | 3 | 33600582 | C | A | 0.028 | 0.046 | 0.006 | 1.62E-15 | 63 |
| rs140440099 | 3 | 50632595 | A | G | 0.023 | 0.061 | 0.006 | 1.45E-22 | 95 |
| rs17718736 | 3 | 71555205 | A | C | 0.323 | 0.012 | 0.002 | 1.39E-08 | 33 |
| rs4682483 | 3 | 112993982 | A | G | 0.153 | -0.017 | 0.003 | 2.65E-10 | 40 |
| rs4683435 | 3 | 142624732 | G | A | 0.772 | 0.014 | 0.002 | 1.60E-10 | 43 |
| rs900399 | 3 | 156798732 | G | A | 0.399 | 0.016 | 0.002 | 1.35E-17 | 75 |
| rs1290786 | 3 | 169097381 | T | C | 0.431 | -0.014 | 0.002 | 7.14E-14 | 57 |
| rs9647379 | 3 | 171785168 | C | G | 0.411 | 0.022 | 0.002 | 5.55E-29 | 128 |
| rs2194411 | 3 | 185548663 | A | G | 0.128 | 0.044 | 0.003 | 2.43E-54 | 233 |
| rs11720869 | 3 | 185619716 | A | G | 0.669 | 0.014 | 0.002 | 2.54E-12 | 50 |
| rs73052033 | 3 | 185828465 | C | T | 0.185 | -0.015 | 0.002 | 4.79E-10 | 40 |
| rs336630 | 3 | 18607538 | T | C | 0.430 | -0.011 | 0.002 | 2.90E-08 | 31 |
| rs9838614 | 3 | 38537671 | G | T | 0.388 | -0.019 | 0.002 | 1.21E-21 | 95 |
| rs6762851 | 3 | 56686329 | C | T | 0.357 | -0.022 | 0.002 | 1.50E-28 | 119 |
| rs182798714 | 3 | 128960869 | T | A | 0.027 | 0.038 | 0.006 | 1.45E-09 | 37 |
| rs591668 | 3 | 27535931 | A | G | 0.396 | -0.017 | 0.002 | 2.00E-19 | 84 |
| rs36012032 | 3 | 52814709 | A | C | 0.091 | 0.030 | 0.003 | 9.93E-20 | 82 |
| rs839255 | 3 | 57974580 | G | T | 0.685 | -0.013 | 0.002 | 8.38E-10 | 36 |
| rs9809116 | 3 | 72397279 | G | A | 0.408 | -0.016 | 0.002 | 1.31E-16 | 71 |
| rs7633464 | 3 | 98715823 | A | G | 0.479 | 0.018 | 0.002 | 1.27E-20 | 85 |
| rs115010283 | 3 | 172162393 | C | G | 0.316 | 0.034 | 0.002 | 2.35E-63 | 289 |
| rs61732778 | 3 | 187443314 | A | G | 0.071 | 0.023 | 0.004 | 3.13E-10 | 39 |
| rs4076108 | 3 | 13736088 | T | A | 0.245 | 0.017 | 0.002 | 2.30E-15 | 63 |
| rs34312629 | 3 | 24079795 | G | C | 0.261 | -0.017 | 0.002 | 2.12E-15 | 66 |
| rs200739311 | 3 | 85650323 | C | T | 0.619 | -0.013 | 0.002 | 1.55E-10 | 41 |
| rs544136 | 3 | 101041229 | G | T | 0.748 | 0.012 | 0.002 | 2.50E-08 | 30 |
| rs4073154 | 3 | 129035485 | G | A | 0.778 | 0.027 | 0.002 | 1.92E-33 | 142 |
| rs2871960 | 3 | 141121814 | C | A | 0.445 | 0.047 | 0.002 | 2.17E-135 | 609 |
| rs1730028 | 3 | 157900789 | G | T | 0.417 | 0.013 | 0.002 | 7.39E-12 | 48 |
| rs71635721 | 3 | 171960170 | G | C | 0.065 | 0.032 | 0.004 | 3.46E-16 | 66 |
| rs34390533 | 3 | 184030838 | A | C | 0.248 | -0.026 | 0.002 | 6.69E-32 | 136 |
| rs7610055 | 3 | 12388409 | A | G | 0.121 | -0.037 | 0.003 | 3.55E-38 | 165 |
| rs56239180 | 3 | 32937951 | G | T | 0.024 | -0.046 | 0.006 | 1.85E-13 | 55 |
| rs9828525 | 3 | 61552810 | T | C | 0.411 | 0.012 | 0.002 | 2.51E-10 | 41 |
| rs116493405 | 3 | 114733556 | A | G | 0.054 | 0.029 | 0.004 | 9.52E-12 | 47 |
| rs9832919 | 3 | 132184526 | G | A | 0.357 | -0.018 | 0.002 | 7.90E-20 | 80 |
| rs1823217 | 3 | 134380959 | G | A | 0.645 | -0.018 | 0.002 | 4.06E-20 | 82 |
| rs11461979 | 3 | 196887239 | TA | T | 0.265 | 0.014 | 0.002 | 9.80E-11 | 42 |
| rs113289555 | 4 | 996998 | T | G | 0.233 | -0.021 | 0.002 | 7.33E-20 | 80 |
| rs13127468 | 4 | 8599658 | A | C | 0.463 | -0.012 | 0.002 | 9.86E-11 | 42 |
| rs10005035 | 4 | 12865684 | G | C | 0.282 | -0.018 | 0.002 | 6.51E-17 | 69 |
| rs1472852 | 4 | 17910236 | A | C | 0.158 | -0.064 | 0.003 | 8.22E-135 | 602 |
| rs963317 | 4 | 45129970 | G | A | 0.663 | -0.014 | 0.002 | 1.03E-11 | 46 |
| rs781669 | 4 | 57819794 | T | C | 0.524 | 0.016 | 0.002 | 3.14E-18 | 75 |
| rs13103161 | 4 | 106216459 | A | T | 0.389 | -0.028 | 0.002 | 2.38E-48 | 223 |
| rs6849302 | 4 | 156665074 | G | A | 0.198 | 0.016 | 0.002 | 7.11E-11 | 42 |
| rs1443536 | 4 | 82174165 | G | A | 0.305 | 0.022 | 0.002 | 1.91E-26 | 108 |
| rs72657800 | 4 | 90822051 | C | T | 0.077 | -0.022 | 0.004 | 6.39E-10 | 39 |
| rs11098677 | 4 | 123833516 | T | G | 0.788 | -0.026 | 0.002 | 3.94E-30 | 131 |
| rs12512942 | 4 | 177766307 | A | G | 0.638 | -0.016 | 0.002 | 1.58E-16 | 66 |
| rs59950280 | 4 | 3452345 | A | G | 0.332 | -0.025 | 0.002 | 7.32E-36 | 161 |
| rs10019221 | 4 | 21785364 | T | G | 0.597 | -0.012 | 0.002 | 1.22E-10 | 43 |
| rs36052389 | 4 | 39357183 | AT | A | 0.455 | 0.013 | 0.002 | 5.27E-12 | 48 |
| rs3103223 | 4 | 42402721 | C | T | 0.740 | 0.013 | 0.002 | 6.02E-09 | 33 |
| rs139921635 | 4 | 73181637 | T | G | 0.024 | 0.039 | 0.006 | 6.16E-10 | 39 |
| rs111612346 | 4 | 109098374 | ATTCTT | A | 0.640 | -0.014 | 0.002 | 4.25E-12 | 48 |
| rs34548509 | 4 | 119599469 | GA | G | 0.357 | 0.012 | 0.002 | 3.20E-09 | 35 |
| rs13123591 | 4 | 120105990 | G | T | 0.338 | 0.019 | 0.002 | 2.35E-20 | 86 |
| rs6821305 | 4 | 122713863 | C | A | 0.399 | 0.020 | 0.002 | 3.15E-26 | 115 |
| rs72695791 | 4 | 184059452 | G | C | 0.036 | -0.030 | 0.005 | 4.58E-09 | 34 |
| rs2324154 | 4 | 24027226 | A | C | 0.510 | 0.015 | 0.002 | 1.92E-15 | 62 |
| rs116339650 | 4 | 26200972 | G | A | 0.127 | -0.018 | 0.003 | 1.05E-09 | 36 |
| rs2303423 | 4 | 38120029 | C | T | 0.110 | 0.017 | 0.003 | 2.64E-08 | 31 |
| rs13109280 | 4 | 54380513 | G | A | 0.662 | 0.013 | 0.002 | 9.15E-11 | 43 |
| rs116052377 | 4 | 124787756 | A | G | 0.081 | 0.023 | 0.004 | 7.89E-11 | 41 |
| rs7689420 | 4 | 145568352 | C | T | 0.831 | 0.047 | 0.003 | 1.50E-76 | 347 |
| rs11721522 | 4 | 156976051 | G | A | 0.413 | 0.011 | 0.002 | 4.03E-08 | 31 |
| rs73856768 | 4 | 157788804 | C | T | 0.081 | -0.025 | 0.004 | 1.55E-12 | 50 |
| rs111622870 | 4 | 2613109 | C | T | 0.048 | -0.028 | 0.004 | 1.86E-10 | 41 |
| rs190823861 | 4 | 73505911 | A | G | 0.047 | -0.035 | 0.005 | 2.09E-14 | 59 |
| rs148617731 | 4 | 87616668 | CAG | C | 0.052 | 0.041 | 0.004 | 5.92E-22 | 91 |
| rs11727162 | 4 | 88606761 | T | C | 0.499 | -0.017 | 0.002 | 2.15E-19 | 80 |
| rs2035901 | 4 | 145521867 | G | A | 0.468 | 0.024 | 0.002 | 9.43E-37 | 160 |
| rs7679276 | 4 | 146860186 | G | A | 0.954 | -0.033 | 0.005 | 5.93E-12 | 47 |
| rs395980 | 4 | 177430072 | G | T | 0.264 | -0.018 | 0.002 | 1.02E-17 | 77 |
| rs2578565 | 5 | 5460569 | T | C | 0.658 | -0.014 | 0.002 | 1.37E-12 | 50 |
| rs12655296 | 5 | 15890643 | T | C | 0.625 | -0.011 | 0.002 | 1.62E-08 | 30 |
| rs1177765 | 5 | 32829929 | C | T | 0.468 | -0.023 | 0.002 | 1.32E-34 | 149 |
| rs11959466 | 5 | 42803824 | T | C | 0.056 | 0.038 | 0.004 | 2.24E-19 | 82 |
| rs62370472 | 5 | 52767109 | C | T | 0.209 | -0.025 | 0.002 | 1.45E-27 | 121 |
| rs10471339 | 5 | 67823773 | G | C | 0.382 | -0.011 | 0.002 | 1.45E-08 | 34 |
| rs36048468 | 5 | 122879901 | T | C | 0.209 | 0.025 | 0.002 | 9.34E-28 | 122 |
| rs7735891 | 5 | 131597005 | T | C | 0.463 | 0.026 | 0.002 | 1.14E-42 | 186 |
| rs55758152 | 5 | 171317318 | A | G | 0.326 | 0.015 | 0.002 | 1.05E-12 | 53 |
| rs57059662 | 5 | 33217275 | C | T | 0.677 | 0.012 | 0.002 | 5.63E-09 | 35 |
| rs34313173 | 5 | 36803614 | TA | T | 0.448 | -0.028 | 0.002 | 3.17E-50 | 223 |
| rs10461725 | 5 | 39437129 | C | G | 0.656 | 0.013 | 0.002 | 1.98E-11 | 45 |
| rs4865956 | 5 | 54882505 | A | T | 0.697 | -0.026 | 0.002 | 3.85E-36 | 151 |
| rs12517711 | 5 | 60754661 | C | T | 0.392 | -0.015 | 0.002 | 2.79E-14 | 60 |
| rs12188208 | 5 | 77442791 | C | A | 0.235 | -0.020 | 0.002 | 1.40E-18 | 79 |
| rs115912456 | 5 | 82815158 | G | A | 0.041 | 0.058 | 0.005 | 3.69E-34 | 151 |
| rs861674 | 5 | 112064475 | T | A | 0.458 | 0.013 | 0.002 | 1.36E-11 | 45 |
| rs10068640 | 5 | 123981977 | A | G | 0.365 | 0.011 | 0.002 | 1.28E-08 | 31 |
| rs2545339 | 5 | 149911219 | G | A | 0.629 | 0.012 | 0.002 | 3.48E-09 | 33 |
| rs144622623 | 5 | 156734644 | GGTGTGTGTGT | G | 0.746 | 0.015 | 0.002 | 4.67E-11 | 43 |
| rs31196 | 5 | 158300798 | A | C | 0.572 | -0.011 | 0.002 | 2.07E-08 | 32 |
| rs111365325 | 5 | 170865229 | T | C | 0.231 | -0.027 | 0.002 | 1.12E-33 | 152 |
| rs6874142 | 5 | 172753555 | G | T | 0.114 | 0.029 | 0.003 | 5.15E-20 | 86 |
| rs10075249 | 5 | 52846505 | T | C | 0.495 | 0.014 | 0.002 | 4.56E-14 | 57 |
| rs10036789 | 5 | 71695918 | G | C | 0.461 | 0.016 | 0.002 | 1.16E-17 | 74 |
| rs33986149 | 5 | 88270485 | CA | C | 0.388 | -0.015 | 0.002 | 2.23E-15 | 66 |
| rs261223 | 5 | 95901046 | C | A | 0.370 | 0.018 | 0.002 | 2.30E-19 | 85 |
| rs12519407 | 5 | 137651012 | C | A | 0.257 | 0.018 | 0.002 | 3.38E-17 | 68 |
| rs249677 | 5 | 141539339 | A | C | 0.633 | -0.011 | 0.002 | 2.40E-08 | 30 |
| rs13170063 | 5 | 157895013 | A | G | 0.592 | -0.015 | 0.002 | 4.11E-15 | 64 |
| rs447352 | 5 | 678750 | T | C | 0.141 | -0.018 | 0.003 | 6.62E-10 | 39 |
| rs7731023 | 5 | 36181627 | G | A | 0.575 | 0.017 | 0.002 | 3.48E-18 | 76 |
| rs7448554 | 5 | 95711603 | A | C | 0.500 | -0.013 | 0.002 | 1.77E-11 | 44 |
| rs3822742 | 5 | 139059017 | A | C | 0.371 | 0.016 | 0.002 | 1.03E-16 | 66 |
| rs4282339 | 5 | 168256240 | A | G | 0.208 | -0.031 | 0.002 | 6.16E-41 | 183 |
| rs244711 | 5 | 176509193 | T | C | 0.686 | 0.028 | 0.002 | 1.54E-37 | 161 |
| rs40270 | 5 | 55804552 | C | A | 0.772 | 0.015 | 0.002 | 1.90E-11 | 47 |
| rs34287 | 5 | 67585143 | A | G | 0.341 | 0.019 | 0.002 | 1.17E-20 | 87 |
| rs331917 | 5 | 98158524 | G | A | 0.580 | -0.013 | 0.002 | 3.54E-11 | 45 |
| rs6860245 | 5 | 127367998 | C | G | 0.248 | 0.059 | 0.002 | 9.66E-160 | 717 |
| rs4976262 | 5 | 134379531 | C | T | 0.316 | -0.025 | 0.002 | 4.37E-33 | 150 |
| rs258794 | 5 | 142540040 | T | G | 0.276 | 0.015 | 0.002 | 6.23E-12 | 49 |
| rs7701233 | 5 | 171218388 | C | T | 0.428 | -0.018 | 0.002 | 5.12E-21 | 89 |
| rs3792819 | 5 | 172576296 | G | A | 0.085 | 0.021 | 0.003 | 4.42E-10 | 38 |
| rs11243202 | 6 | 7719065 | C | T | 0.486 | 0.030 | 0.002 | 2.83E-57 | 253 |
| rs13209685 | 6 | 7779729 | T | G | 0.159 | 0.028 | 0.003 | 7.49E-27 | 114 |
| rs2142644 | 6 | 19053843 | A | C | 0.672 | -0.018 | 0.002 | 3.37E-19 | 82 |
| rs78000963 | 6 | 30502802 | A | G | 0.104 | 0.017 | 0.003 | 4.39E-08 | 30 |
| rs370927791 | 6 | 33621055 | CT | C | 0.496 | -0.021 | 0.002 | 4.99E-25 | 108 |
| rs2268718 | 6 | 52415023 | T | C | 0.270 | 0.014 | 0.002 | 3.24E-11 | 45 |
| rs6931421 | 6 | 80880138 | G | T | 0.322 | -0.028 | 0.002 | 2.31E-43 | 195 |
| rs9375188 | 6 | 98555272 | T | C | 0.484 | 0.014 | 0.002 | 6.80E-13 | 51 |
| rs9391254 | 6 | 105377347 | T | C | 0.321 | 0.017 | 0.002 | 2.10E-16 | 69 |
| rs113898003 | 6 | 130341235 | C | T | 0.264 | -0.036 | 0.002 | 1.19E-63 | 294 |
| rs1933081 | 6 | 151651505 | A | T | 0.083 | 0.027 | 0.003 | 5.33E-15 | 62 |
| rs141641494 | 6 | 53342562 | CT | C | 0.369 | -0.012 | 0.002 | 8.39E-09 | 33 |
| rs9385002 | 6 | 117552469 | T | A | 0.239 | -0.015 | 0.002 | 3.26E-11 | 45 |
| rs7768382 | 6 | 166341870 | C | T | 0.477 | -0.020 | 0.002 | 1.57E-26 | 112 |
| rs372987459 | 6 | 169374339 | TAC | T | 0.604 | 0.015 | 0.002 | 6.85E-14 | 54 |
| rs2788213 | 6 | 703249 | A | G | 0.710 | 0.012 | 0.002 | 3.80E-09 | 34 |
| rs2569888 | 6 | 1625803 | A | G | 0.245 | 0.013 | 0.002 | 2.29E-09 | 37 |
| rs876122 | 6 | 6886297 | G | A | 0.879 | 0.016 | 0.003 | 2.19E-08 | 31 |
| rs41271299 | 6 | 19839415 | T | C | 0.051 | 0.062 | 0.004 | 3.19E-47 | 205 |
| rs188617336 | 6 | 20610730 | T | C | 0.298 | 0.014 | 0.002 | 6.66E-11 | 43 |
| rs4380799 | 6 | 32571864 | G | T | 0.390 | -0.026 | 0.002 | 6.45E-33 | 147 |
| rs1319012 | 6 | 41852616 | A | T | 0.926 | -0.052 | 0.004 | 3.30E-45 | 198 |
| rs1324538 | 6 | 45080144 | A | T | 0.384 | 0.024 | 0.002 | 1.73E-34 | 156 |
| rs655113 | 6 | 52269151 | C | T | 0.301 | 0.019 | 0.002 | 7.42E-20 | 80 |
| rs9344126 | 6 | 81907559 | C | T | 0.514 | -0.019 | 0.002 | 2.21E-22 | 95 |
| rs293517 | 6 | 83662455 | C | T | 0.701 | -0.013 | 0.002 | 2.80E-10 | 38 |
| rs7768973 | 6 | 109745325 | A | T | 0.411 | -0.024 | 0.002 | 5.44E-36 | 160 |
| rs78051210 | 6 | 131379491 | C | T | 0.077 | 0.026 | 0.004 | 1.63E-13 | 53 |
| rs10807137 | 6 | 34183026 | T | C | 0.825 | -0.046 | 0.003 | 5.68E-75 | 331 |
| rs9343327 | 6 | 76606296 | T | A | 0.499 | 0.014 | 0.002 | 1.09E-13 | 54 |
| rs35166681 | 6 | 85369727 | TA | T | 0.159 | 0.021 | 0.003 | 1.55E-16 | 68 |
| rs2754255 | 6 | 88393572 | G | A | 0.225 | -0.015 | 0.002 | 1.05E-11 | 44 |
| rs9388490 | 6 | 126704795 | T | C | 0.439 | 0.046 | 0.002 | 1.33E-130 | 591 |
| rs6902109 | 6 | 130316559 | G | A | 0.540 | -0.017 | 0.002 | 1.03E-18 | 77 |
| rs599004 | 6 | 140439740 | T | C | 0.281 | -0.016 | 0.002 | 6.53E-14 | 56 |
| rs2748501 | 6 | 146312258 | A | G | 0.440 | -0.020 | 0.002 | 1.31E-24 | 105 |
| rs718603 | 6 | 2644245 | T | C | 0.277 | 0.013 | 0.002 | 6.68E-10 | 39 |
| rs9266244 | 6 | 31325692 | A | G | 0.708 | -0.043 | 0.002 | 1.21E-94 | 413 |
| rs72894003 | 6 | 34775096 | T | C | 0.065 | -0.042 | 0.004 | 1.90E-28 | 124 |
| rs2764264 | 6 | 108934461 | T | C | 0.695 | 0.020 | 0.002 | 5.07E-23 | 93 |
| rs6570509 | 6 | 142716286 | T | G | 0.287 | -0.024 | 0.002 | 1.27E-31 | 135 |
| rs543650 | 6 | 152110943 | G | T | 0.599 | 0.025 | 0.002 | 1.49E-37 | 156 |
| rs3828729 | 6 | 155554707 | G | A | 0.309 | -0.016 | 0.002 | 4.67E-15 | 64 |
| rs2105333 | 6 | 158755437 | G | T | 0.665 | -0.019 | 0.002 | 1.70E-21 | 90 |
| rs2763263 | 6 | 168814392 | A | T | 0.245 | -0.017 | 0.002 | 1.37E-14 | 60 |
| rs798548 | 7 | 2760935 | C | T | 0.301 | -0.036 | 0.002 | 2.86E-68 | 292 |
| rs12533452 | 7 | 19016871 | T | C | 0.157 | 0.024 | 0.003 | 1.31E-19 | 83 |
| rs34776209 | 7 | 23513093 | T | C | 0.248 | -0.032 | 0.002 | 1.78E-47 | 208 |
| rs12536902 | 7 | 33213009 | A | C | 0.014 | 0.048 | 0.008 | 3.66E-09 | 35 |
| rs1880318 | 7 | 46028167 | A | G | 0.204 | 0.015 | 0.002 | 6.86E-10 | 38 |
| rs73696333 | 7 | 46669400 | G | C | 0.201 | 0.019 | 0.002 | 3.20E-15 | 63 |
| rs35732917 | 7 | 73013269 | C | T | 0.284 | 0.020 | 0.002 | 2.08E-22 | 94 |
| rs139163241 | 7 | 76709213 | G | T | 0.139 | -0.016 | 0.003 | 1.79E-09 | 37 |
| rs62466110 | 7 | 92623541 | C | T | 0.067 | -0.037 | 0.004 | 5.74E-20 | 82 |
| rs143355941 | 7 | 93231238 | TGAGA | T | 0.437 | 0.019 | 0.002 | 4.65E-23 | 99 |
| rs987666 | 7 | 116267938 | A | G | 0.119 | 0.019 | 0.003 | 2.33E-10 | 41 |
| rs62621812 | 7 | 127015083 | A | G | 0.020 | 0.074 | 0.007 | 3.16E-27 | 116 |
| rs757834 | 7 | 139717200 | C | T | 0.186 | 0.026 | 0.002 | 1.25E-25 | 114 |
| rs28529426 | 7 | 4678264 | T | C | 0.171 | -0.017 | 0.003 | 5.82E-11 | 42 |
| rs10242866 | 7 | 17920613 | T | C | 0.398 | 0.016 | 0.002 | 3.66E-16 | 68 |
| rs2529090 | 7 | 24662280 | G | C | 0.181 | 0.014 | 0.003 | 3.39E-08 | 30 |
| rs2237485 | 7 | 50749870 | A | G | 0.223 | 0.019 | 0.002 | 3.73E-17 | 69 |
| rs10627558 | 7 | 135212623 | CGT | C | 0.235 | -0.018 | 0.002 | 6.91E-16 | 68 |
| rs12672217 | 7 | 156310948 | A | G | 0.361 | 0.014 | 0.002 | 1.33E-12 | 48 |
| rs177591 | 7 | 28556199 | G | C | 0.149 | -0.019 | 0.003 | 1.51E-12 | 50 |
| rs60408354 | 7 | 70158495 | A | G | 0.073 | 0.026 | 0.004 | 1.15E-12 | 52 |
| rs42039 | 7 | 92244422 | T | C | 0.244 | 0.048 | 0.002 | 3.53E-106 | 478 |
| rs56363908 | 7 | 96611052 | G | A | 0.042 | -0.038 | 0.005 | 3.88E-16 | 66 |
| rs2140619 | 7 | 114007270 | G | A | 0.413 | 0.011 | 0.002 | 5.18E-09 | 35 |
| rs3778858 | 7 | 129963356 | T | G | 0.370 | 0.011 | 0.002 | 4.26E-08 | 29 |
| rs822530 | 7 | 148631555 | T | A | 0.795 | 0.026 | 0.002 | 2.36E-27 | 113 |
| rs12702693 | 7 | 8101039 | T | C | 0.454 | 0.017 | 0.002 | 6.56E-20 | 83 |
| rs680882 | 7 | 18325278 | G | T | 0.761 | 0.013 | 0.002 | 1.98E-09 | 37 |
| rs723149 | 7 | 46577056 | G | A | 0.563 | -0.028 | 0.002 | 1.43E-47 | 211 |
| rs1202186 | 7 | 87213258 | T | C | 0.655 | -0.012 | 0.002 | 1.65E-09 | 36 |
| rs9640283 | 7 | 150485659 | C | G | 0.518 | -0.012 | 0.002 | 4.53E-10 | 39 |
| rs6977416 | 7 | 150542711 | A | G | 0.334 | 0.046 | 0.002 | 1.43E-113 | 522 |
| rs10225945 | 7 | 28250083 | G | A | 0.150 | -0.015 | 0.003 | 3.28E-08 | 32 |
| rs12700901 | 7 | 28783171 | A | C | 0.406 | -0.018 | 0.002 | 1.67E-21 | 94 |
| rs6593210 | 7 | 55254186 | A | G | 0.208 | 0.015 | 0.002 | 4.88E-10 | 37 |
| rs11562101 | 7 | 56059458 | T | A | 0.458 | 0.012 | 0.002 | 9.39E-09 | 33 |
| rs2188805 | 7 | 93078400 | C | A | 0.336 | 0.011 | 0.002 | 2.00E-08 | 32 |
| rs76364830 | 8 | 13372120 | A | G | 0.063 | -0.047 | 0.004 | 2.70E-33 | 146 |
| rs7826059 | 8 | 22512068 | C | T | 0.642 | 0.011 | 0.002 | 7.61E-09 | 32 |
| rs1063582 | 8 | 23167353 | G | T | 0.765 | -0.019 | 0.002 | 1.12E-16 | 71 |
| rs117818446 | 8 | 67223589 | A | G | 0.020 | 0.042 | 0.007 | 5.29E-10 | 39 |
| rs7014590 | 8 | 89335647 | C | T | 0.261 | -0.023 | 0.002 | 4.48E-26 | 107 |
| rs112537273 | 8 | 38248306 | C | T | 0.230 | -0.021 | 0.002 | 3.34E-21 | 93 |
| rs4602848 | 8 | 92186933 | G | A | 0.681 | 0.016 | 0.002 | 3.14E-15 | 64 |
| rs35086476 | 8 | 97607566 | GA | G | 0.497 | 0.011 | 0.002 | 8.43E-09 | 33 |
| rs2142331 | 8 | 116636719 | T | C | 0.602 | -0.017 | 0.002 | 1.38E-17 | 75 |
| rs6470771 | 8 | 130743726 | C | A | 0.169 | -0.027 | 0.003 | 1.54E-26 | 115 |
| rs12334478 | 8 | 141998765 | G | C | 0.496 | -0.016 | 0.002 | 2.27E-17 | 72 |
| rs10112506 | 8 | 13164746 | G | A | 0.390 | -0.012 | 0.002 | 5.76E-10 | 40 |
| rs7816345 | 8 | 36846109 | T | C | 0.168 | 0.026 | 0.003 | 6.22E-24 | 104 |
| rs2923411 | 8 | 42455206 | C | T | 0.595 | 0.013 | 0.002 | 4.77E-11 | 45 |
| rs72656010 | 8 | 57122215 | C | T | 0.132 | -0.067 | 0.003 | 7.31E-126 | 569 |
| rs7828086 | 8 | 120843775 | C | T | 0.239 | 0.014 | 0.002 | 1.11E-09 | 38 |
| rs62501195 | 8 | 24041988 | C | A | 0.171 | -0.020 | 0.003 | 8.11E-15 | 63 |
| rs62515437 | 8 | 57160328 | T | G | 0.225 | 0.037 | 0.002 | 8.79E-60 | 257 |
| rs2925155 | 8 | 75886297 | T | C | 0.261 | -0.015 | 0.002 | 5.47E-12 | 46 |
| rs11778491 | 8 | 120451362 | C | G | 0.252 | -0.025 | 0.002 | 8.39E-30 | 126 |
| rs1340022 | 8 | 131334465 | C | T | 0.487 | 0.012 | 0.002 | 4.47E-10 | 39 |
| rs72721979 | 8 | 135827942 | G | T | 0.143 | -0.023 | 0.003 | 2.20E-17 | 72 |
| rs7007389 | 8 | 25355022 | A | T | 0.373 | -0.013 | 0.002 | 2.94E-11 | 45 |
| rs115105539 | 8 | 49409496 | C | G | 0.167 | 0.023 | 0.003 | 7.83E-20 | 85 |
| rs4077103 | 8 | 49557732 | A | C | 0.843 | -0.014 | 0.003 | 4.18E-08 | 30 |
| rs61729527 | 8 | 77761919 | T | C | 0.052 | -0.035 | 0.004 | 4.86E-16 | 65 |
| rs4735761 | 8 | 78097161 | C | A | 0.286 | 0.033 | 0.002 | 3.66E-56 | 248 |
| rs10283100 | 8 | 120596023 | G | A | 0.945 | 0.058 | 0.004 | 4.11E-44 | 197 |
| rs4870941 | 8 | 126498828 | C | G | 0.238 | -0.030 | 0.002 | 1.09E-39 | 167 |
| rs12541381 | 8 | 135649848 | A | G | 0.258 | -0.032 | 0.002 | 2.81E-49 | 210 |
| rs10107388 | 8 | 145004944 | C | T | 0.369 | -0.016 | 0.002 | 6.95E-16 | 63 |
| rs10815274 | 9 | 5728968 | C | A | 0.456 | 0.012 | 0.002 | 6.46E-11 | 43 |
| rs7858712 | 9 | 16738312 | G | A | 0.914 | 0.035 | 0.003 | 1.04E-24 | 104 |
| rs34522021 | 9 | 23350420 | T | C | 0.455 | 0.013 | 0.002 | 3.38E-11 | 44 |
| rs75508358 | 9 | 96926382 | T | C | 0.046 | 0.027 | 0.005 | 4.29E-09 | 35 |
| rs12347137 | 9 | 119122721 | C | A | 0.202 | -0.046 | 0.002 | 9.80E-85 | 367 |
| rs10123619 | 9 | 119353611 | G | A | 0.843 | -0.017 | 0.003 | 4.05E-11 | 43 |
| rs10975935 | 9 | 6954579 | G | A | 0.246 | -0.012 | 0.002 | 4.15E-08 | 30 |
| rs1056747 | 9 | 35690102 | G | A | 0.412 | -0.016 | 0.002 | 8.05E-16 | 67 |
| rs143554698 | 9 | 95538573 | T | C | 0.141 | -0.026 | 0.003 | 3.39E-21 | 91 |
| rs10982888 | 9 | 118468947 | A | T | 0.114 | -0.033 | 0.003 | 3.97E-28 | 120 |
| rs73384223 | 9 | 3869315 | C | T | 0.197 | -0.021 | 0.002 | 1.27E-17 | 73 |
| rs10962212 | 9 | 15911745 | C | G | 0.418 | 0.014 | 0.002 | 7.47E-14 | 57 |
| rs7863102 | 9 | 73963468 | T | A | 0.447 | -0.011 | 0.002 | 9.97E-09 | 34 |
| rs3901421 | 9 | 96204538 | C | G | 0.487 | 0.022 | 0.002 | 7.55E-30 | 128 |
| rs2236406 | 9 | 98221861 | C | T | 0.349 | 0.039 | 0.002 | 1.26E-87 | 388 |
| rs373966865 | 9 | 99089087 | CA | C | 0.176 | 0.039 | 0.003 | 1.84E-53 | 242 |
| rs3205136 | 9 | 136126631 | A | C | 0.095 | -0.018 | 0.003 | 1.85E-08 | 31 |
| rs10858246 | 9 | 139102831 | C | G | 0.318 | -0.019 | 0.002 | 2.35E-20 | 88 |
| rs1330826 | 9 | 85129970 | C | G | 0.227 | 0.016 | 0.002 | 1.04E-12 | 50 |
| rs7020491 | 9 | 128144477 | T | C | 0.427 | -0.018 | 0.002 | 1.22E-20 | 88 |
| rs74458759 | 9 | 136940614 | G | C | 0.289 | 0.017 | 0.002 | 3.12E-15 | 60 |
| rs12340775 | 9 | 13226945 | A | G | 0.055 | -0.029 | 0.004 | 1.73E-11 | 45 |
| rs12351226 | 9 | 98405230 | T | C | 0.171 | 0.022 | 0.003 | 9.13E-18 | 76 |
| rs1341215 | 9 | 111662350 | A | G | 0.137 | 0.023 | 0.003 | 6.32E-17 | 72 |
| rs12344515 | 9 | 113801231 | T | C | 0.240 | -0.016 | 0.002 | 2.28E-13 | 55 |
| rs80280630 | 9 | 117030861 | T | C | 0.112 | -0.017 | 0.003 | 2.28E-08 | 31 |
| rs112367251 | 9 | 129738592 | TCTCACA | T | 0.357 | 0.018 | 0.002 | 1.29E-17 | 73 |
| rs10793931 | 9 | 133436478 | C | G | 0.356 | -0.013 | 0.002 | 3.23E-11 | 44 |
| rs7082659 | 10 | 12017584 | C | T | 0.866 | 0.016 | 0.003 | 2.27E-08 | 31 |
| rs10829226 | 10 | 27573952 | A | G | 0.636 | -0.011 | 0.002 | 1.33E-08 | 31 |
| rs10776560 | 10 | 50542358 | T | C | 0.500 | -0.016 | 0.002 | 7.88E-17 | 68 |
| rs68049170 | 10 | 72432047 | A | G | 0.276 | -0.026 | 0.002 | 2.69E-34 | 152 |
| rs2274351 | 10 | 104264107 | T | C | 0.543 | 0.017 | 0.002 | 3.07E-19 | 80 |
| rs72841270 | 10 | 104642237 | G | T | 0.135 | 0.029 | 0.003 | 2.25E-26 | 110 |
| rs11421589 | 10 | 115193949 | CT | C | 0.556 | 0.012 | 0.002 | 5.99E-10 | 39 |
| rs11424084 | 10 | 126612510 | CA | C | 0.140 | 0.023 | 0.003 | 7.24E-17 | 71 |
| rs1556659 | 10 | 130834698 | T | C | 0.382 | 0.016 | 0.002 | 7.19E-17 | 66 |
| rs35288270 | 10 | 4961278 | C | T | 0.134 | -0.033 | 0.003 | 3.43E-32 | 137 |
| rs4748008 | 10 | 12935125 | C | T | 0.435 | -0.013 | 0.002 | 8.95E-11 | 43 |
| rs332116 | 10 | 28926099 | T | C | 0.280 | -0.021 | 0.002 | 2.89E-22 | 96 |
| rs10822117 | 10 | 52786701 | G | A | 0.237 | -0.018 | 0.002 | 4.24E-15 | 64 |
| rs67527161 | 10 | 63781824 | C | T | 0.209 | -0.018 | 0.002 | 5.79E-15 | 63 |
| rs10128333 | 10 | 64570038 | T | C | 0.168 | -0.015 | 0.003 | 9.51E-09 | 34 |
| rs7095472 | 10 | 70399109 | G | A | 0.534 | 0.027 | 0.002 | 7.66E-45 | 197 |
| rs117335233 | 10 | 79914330 | G | T | 0.053 | -0.024 | 0.004 | 2.56E-08 | 32 |
| rs12773500 | 10 | 81232632 | T | C | 0.138 | 0.017 | 0.003 | 5.05E-10 | 37 |
| rs11187838 | 10 | 96038686 | A | G | 0.435 | 0.039 | 0.002 | 1.16E-94 | 430 |
| rs2181834 | 10 | 102661251 | T | G | 0.550 | 0.025 | 0.002 | 7.69E-41 | 179 |
| rs496783 | 10 | 116137961 | G | A | 0.465 | -0.012 | 0.002 | 8.13E-11 | 43 |
| rs11198591 | 10 | 120515892 | A | G | 0.368 | 0.015 | 0.002 | 4.91E-14 | 55 |
| rs2362487 | 10 | 126208402 | G | C | 0.247 | 0.015 | 0.002 | 3.57E-12 | 49 |
| rs947099 | 10 | 31129883 | A | G | 0.354 | 0.012 | 0.002 | 2.71E-09 | 34 |
| rs71463518 | 10 | 65130905 | AT | A | 0.319 | -0.013 | 0.002 | 2.92E-10 | 40 |
| rs10824307 | 10 | 77185310 | C | G | 0.647 | -0.019 | 0.002 | 1.79E-22 | 94 |
| rs664317 | 10 | 89812230 | C | A | 0.838 | -0.018 | 0.003 | 4.50E-12 | 46 |
| rs2648725 | 10 | 93015079 | A | T | 0.213 | 0.017 | 0.002 | 8.20E-13 | 51 |
| rs7893378 | 10 | 93634095 | A | G | 0.114 | 0.018 | 0.003 | 2.36E-08 | 32 |
| rs291979 | 10 | 121129797 | A | G | 0.229 | 0.024 | 0.002 | 6.76E-27 | 111 |
| rs11009928 | 10 | 35058712 | G | A | 0.255 | -0.015 | 0.002 | 1.04E-11 | 45 |
| rs5786398 | 10 | 81143387 | CT | C | 0.377 | -0.018 | 0.002 | 1.54E-17 | 71 |
| rs4752689 | 10 | 124131176 | A | G | 0.584 | 0.021 | 0.002 | 1.36E-26 | 116 |
| rs11014285 | 10 | 25178864 | A | G | 0.165 | 0.034 | 0.003 | 2.89E-40 | 173 |
| rs2490302 | 10 | 37702435 | A | T | 0.914 | 0.022 | 0.003 | 6.24E-11 | 42 |
| rs11191208 | 10 | 103838497 | A | G | 0.206 | 0.015 | 0.002 | 3.69E-10 | 38 |
| rs10749157 | 10 | 115780129 | C | T | 0.358 | 0.011 | 0.002 | 1.24E-08 | 32 |
| rs2283200 | 11 | 2729340 | T | C | 0.056 | -0.028 | 0.004 | 1.48E-11 | 45 |
| rs73413540 | 11 | 3090976 | T | C | 0.225 | -0.012 | 0.002 | 4.42E-08 | 29 |
| rs985136 | 11 | 17497794 | G | C | 0.490 | 0.014 | 0.002 | 2.96E-12 | 48 |
| rs4752829 | 11 | 47396654 | A | G | 0.286 | 0.026 | 0.002 | 5.90E-36 | 156 |
| rs10796828 | 11 | 69490346 | G | T | 0.635 | 0.015 | 0.002 | 5.77E-15 | 59 |
| rs7902 | 11 | 95565288 | G | A | 0.447 | 0.015 | 0.002 | 5.22E-15 | 61 |
| rs11221657 | 11 | 129181358 | G | T | 0.135 | 0.018 | 0.003 | 1.21E-10 | 41 |
| rs112873218 | 11 | 1960119 | T | C | 0.105 | 0.022 | 0.003 | 4.02E-12 | 49 |
| rs10832963 | 11 | 18664241 | G | T | 0.745 | -0.020 | 0.002 | 9.23E-21 | 85 |
| rs704660 | 11 | 30447998 | T | C | 0.410 | 0.015 | 0.002 | 2.28E-15 | 65 |
| rs7107356 | 11 | 47676170 | G | A | 0.507 | 0.013 | 0.002 | 1.86E-12 | 49 |
| rs11233117 | 11 | 69924352 | G | C | 0.454 | -0.018 | 0.002 | 1.74E-20 | 86 |
| rs73006226 | 11 | 108072728 | A | C | 0.127 | -0.018 | 0.003 | 2.23E-10 | 39 |
| rs545104 | 11 | 118591352 | C | T | 0.616 | 0.013 | 0.002 | 8.08E-11 | 40 |
| rs56207600 | 11 | 126196537 | A | G | 0.111 | 0.019 | 0.003 | 2.50E-10 | 41 |
| rs61878760 | 11 | 12807189 | A | G | 0.083 | 0.019 | 0.003 | 3.74E-08 | 31 |
| rs7952436 | 11 | 67024534 | T | C | 0.082 | -0.045 | 0.003 | 1.62E-39 | 178 |
| rs4244809 | 11 | 2164333 | A | G | 0.208 | -0.026 | 0.002 | 5.55E-29 | 130 |
| rs1584011 | 11 | 27080527 | G | T | 0.356 | 0.016 | 0.002 | 9.78E-16 | 63 |
| rs7941305 | 11 | 28652116 | C | T | 0.313 | -0.013 | 0.002 | 6.01E-10 | 38 |
| rs11605297 | 11 | 58296806 | A | G | 0.233 | 0.015 | 0.002 | 8.03E-11 | 44 |
| rs7129320 | 11 | 68388220 | A | G | 0.166 | -0.039 | 0.003 | 7.29E-53 | 242 |
| rs34345560 | 11 | 69081998 | A | G | 0.195 | 0.022 | 0.002 | 7.10E-20 | 83 |
| rs604723 | 11 | 100610546 | C | T | 0.725 | -0.017 | 0.002 | 8.16E-15 | 62 |
| rs11042717 | 11 | 10303939 | C | T | 0.490 | -0.029 | 0.002 | 4.04E-53 | 233 |
| rs10657263 | 11 | 49690460 | G | C | 0.548 | -0.013 | 0.002 | 7.98E-12 | 47 |
| rs4938359 | 11 | 117093560 | G | A | 0.202 | -0.016 | 0.002 | 3.24E-11 | 42 |
| rs11217863 | 11 | 120293138 | A | G | 0.116 | -0.027 | 0.003 | 1.06E-19 | 80 |
| rs772222 | 12 | 52356892 | G | A | 0.265 | 0.012 | 0.002 | 1.55E-08 | 33 |
| rs1168768 | 12 | 66509650 | T | C | 0.975 | 0.033 | 0.006 | 3.61E-08 | 31 |
| rs2089111 | 12 | 91180019 | G | C | 0.267 | -0.017 | 0.002 | 1.73E-15 | 61 |
| rs4622329 | 12 | 102321935 | A | G | 0.350 | 0.015 | 0.002 | 8.62E-14 | 56 |
| rs3764002 | 12 | 108618630 | T | C | 0.262 | 0.028 | 0.002 | 4.47E-39 | 178 |
| rs12423821 | 12 | 132650284 | C | T | 0.158 | 0.016 | 0.003 | 1.17E-09 | 36 |
| rs7137546 | 12 | 577237 | T | A | 0.425 | 0.014 | 0.002 | 9.71E-14 | 56 |
| rs67551338 | 12 | 3393100 | T | C | 0.062 | 0.058 | 0.004 | 1.04E-47 | 207 |
| rs10845408 | 12 | 11880581 | T | C | 0.354 | 0.026 | 0.002 | 3.25E-38 | 163 |
| rs17478946 | 12 | 24093062 | G | A | 0.300 | -0.019 | 0.002 | 9.98E-21 | 84 |
| rs12230946 | 12 | 53498725 | A | G | 0.091 | 0.027 | 0.003 | 1.45E-16 | 67 |
| rs9669278 | 12 | 66374587 | C | T | 0.518 | -0.050 | 0.002 | 5.25E-151 | 681 |
| rs2229840 | 12 | 124826462 | T | C | 0.160 | 0.034 | 0.003 | 3.02E-40 | 172 |
| rs7485647 | 12 | 131631133 | A | G | 0.844 | -0.026 | 0.003 | 1.04E-23 | 101 |
| rs35756741 | 12 | 12868701 | T | C | 0.092 | -0.038 | 0.003 | 5.80E-31 | 131 |
| rs6582398 | 12 | 42870444 | T | C | 0.600 | 0.014 | 0.002 | 1.14E-12 | 49 |
| rs10748128 | 12 | 69827658 | T | G | 0.345 | 0.026 | 0.002 | 6.77E-38 | 163 |
| rs11178643 | 12 | 71522437 | T | A | 0.359 | 0.011 | 0.002 | 4.44E-08 | 30 |
| rs310796 | 12 | 77453226 | T | G | 0.681 | 0.014 | 0.002 | 2.53E-12 | 50 |
| rs9634212 | 12 | 93993266 | A | C | 0.221 | 0.047 | 0.002 | 8.59E-95 | 419 |
| rs7971536 | 12 | 102373788 | A | T | 0.495 | -0.019 | 0.002 | 1.06E-24 | 104 |
| rs2454390 | 12 | 103255613 | C | T | 0.846 | -0.018 | 0.003 | 1.74E-11 | 46 |
| rs11612462 | 12 | 104411368 | G | T | 0.170 | 0.015 | 0.003 | 2.55E-09 | 36 |
| rs34338597 | 12 | 106301580 | G | A | 0.383 | -0.011 | 0.002 | 7.88E-09 | 35 |
| rs3184504 | 12 | 111884608 | C | T | 0.517 | 0.018 | 0.002 | 2.71E-22 | 93 |
| rs610694 | 12 | 121304826 | C | T | 0.490 | 0.014 | 0.002 | 4.29E-13 | 51 |
| rs76895963 | 12 | 4384844 | G | T | 0.021 | 0.164 | 0.007 | 8.22E-112 | 504 |
| rs11175919 | 12 | 66180277 | A | G | 0.026 | 0.035 | 0.006 | 3.16E-09 | 35 |
| rs11068230 | 12 | 117349014 | G | C | 0.866 | 0.024 | 0.003 | 9.67E-18 | 72 |
| rs2101017 | 12 | 122306857 | T | C | 0.870 | -0.022 | 0.003 | 1.44E-15 | 63 |
| rs11060942 | 12 | 123434524 | A | G | 0.034 | -0.035 | 0.005 | 7.49E-12 | 46 |
| rs28592876 | 12 | 123866429 | A | G | 0.205 | 0.030 | 0.002 | 9.09E-38 | 170 |
| rs3782811 | 12 | 3339927 | A | C | 0.251 | -0.017 | 0.002 | 3.96E-14 | 56 |
| rs61919240 | 12 | 8831954 | A | T | 0.324 | 0.014 | 0.002 | 9.77E-12 | 47 |
| rs1444628 | 12 | 20563643 | T | C | 0.690 | 0.024 | 0.002 | 6.85E-32 | 144 |
| rs11049704 | 12 | 28691701 | G | C | 0.293 | -0.018 | 0.002 | 1.10E-18 | 76 |
| rs12831751 | 12 | 29520017 | C | A | 0.286 | 0.017 | 0.002 | 1.57E-16 | 67 |
| rs12099669 | 12 | 46783653 | A | G | 0.696 | 0.033 | 0.002 | 1.39E-58 | 274 |
| rs2071450 | 12 | 54428532 | T | C | 0.368 | -0.017 | 0.002 | 8.85E-19 | 76 |
| rs3782232 | 12 | 57116249 | A | G | 0.071 | -0.034 | 0.004 | 2.41E-20 | 84 |
| rs7301341 | 12 | 94083105 | C | T | 0.327 | -0.026 | 0.002 | 9.27E-37 | 163 |
| rs7321635 | 13 | 21472055 | C | A | 0.647 | -0.013 | 0.002 | 2.58E-11 | 44 |
| rs3116602 | 13 | 51111355 | G | T | 0.215 | -0.061 | 0.002 | 9.53E-155 | 708 |
| rs3818416 | 13 | 78474468 | C | A | 0.765 | 0.028 | 0.002 | 2.01E-35 | 161 |
| rs61944841 | 13 | 27049616 | A | G | 0.414 | 0.025 | 0.002 | 3.54E-37 | 160 |
| rs77013652 | 13 | 51142279 | G | T | 0.015 | 0.049 | 0.008 | 1.50E-09 | 37 |
| rs7328187 | 13 | 74189974 | G | T | 0.498 | 0.012 | 0.002 | 1.19E-09 | 37 |
| rs7320878 | 13 | 91994132 | A | G | 0.603 | -0.015 | 0.002 | 1.34E-14 | 62 |
| rs144109601 | 13 | 50455500 | A | C | 0.042 | -0.028 | 0.005 | 5.15E-09 | 34 |
| rs9590328 | 13 | 96448383 | G | A | 0.142 | 0.015 | 0.003 | 2.02E-08 | 32 |
| rs78525785 | 13 | 111038331 | T | C | 0.620 | -0.017 | 0.002 | 9.81E-18 | 71 |
| rs9594714 | 13 | 42800481 | T | G | 0.305 | 0.014 | 0.002 | 2.65E-12 | 47 |
| rs9568031 | 13 | 48897520 | T | C | 0.706 | -0.012 | 0.002 | 3.34E-08 | 30 |
| rs2812208 | 13 | 50707087 | C | G | 0.021 | 0.116 | 0.007 | 5.51E-68 | 307 |
| rs8000973 | 13 | 100691367 | C | T | 0.533 | 0.013 | 0.002 | 2.05E-12 | 50 |
| rs9525326 | 13 | 115075715 | G | A | 0.188 | -0.018 | 0.002 | 3.62E-14 | 59 |
| rs532499 | 13 | 30165465 | C | T | 0.741 | -0.013 | 0.002 | 4.90E-09 | 33 |
| rs9517483 | 13 | 99572712 | G | A | 0.699 | -0.018 | 0.002 | 2.26E-18 | 74 |
| rs2296316 | 14 | 65520246 | C | T | 0.464 | -0.019 | 0.002 | 1.59E-23 | 102 |
| rs113827862 | 14 | 89849527 | C | T | 0.061 | -0.024 | 0.004 | 4.67E-09 | 35 |
| rs4900578 | 14 | 103926010 | T | A | 0.369 | -0.018 | 0.002 | 1.76E-19 | 78 |
| rs56112295 | 14 | 105877057 | T | C | 0.226 | 0.015 | 0.002 | 1.12E-10 | 41 |
| rs8019890 | 14 | 21538067 | A | C | 0.531 | 0.025 | 0.002 | 1.96E-38 | 173 |
| rs8017006 | 14 | 42745052 | G | A | 0.328 | 0.012 | 0.002 | 2.22E-09 | 37 |
| rs10483727 | 14 | 61072875 | C | T | 0.611 | -0.037 | 0.002 | 6.73E-80 | 375 |
| rs8020095 | 14 | 67453858 | A | G | 0.150 | -0.015 | 0.003 | 4.53E-08 | 29 |
| rs35230100 | 14 | 70348141 | CATAT | C | 0.433 | -0.026 | 0.002 | 4.42E-40 | 172 |
| rs117068593 | 14 | 93118229 | T | C | 0.190 | 0.040 | 0.002 | 8.83E-62 | 282 |
| rs1190540 | 14 | 102897009 | G | A | 0.698 | 0.013 | 0.002 | 1.62E-09 | 35 |
| rs17197114 | 14 | 21894526 | C | T | 0.177 | 0.018 | 0.003 | 1.54E-12 | 50 |
| rs45528934 | 14 | 23793305 | T | C | 0.162 | 0.026 | 0.003 | 1.97E-24 | 102 |
| rs28529055 | 14 | 92428216 | T | G | 0.437 | -0.015 | 0.002 | 1.88E-14 | 60 |
| rs36226649 | 14 | 24835500 | C | T | 0.067 | 0.049 | 0.004 | 3.05E-37 | 163 |
| rs28678024 | 14 | 25937161 | G | A | 0.285 | -0.012 | 0.002 | 1.90E-08 | 32 |
| rs8904 | 14 | 35871217 | A | G | 0.363 | -0.016 | 0.002 | 1.52E-15 | 62 |
| rs10637890 | 14 | 54779522 | TTC | T | 0.621 | -0.012 | 0.002 | 3.96E-10 | 38 |
| rs2070598 | 14 | 75360906 | A | G | 0.456 | 0.020 | 0.002 | 6.36E-27 | 115 |
| rs909220 | 14 | 75908780 | A | G | 0.444 | -0.015 | 0.002 | 3.24E-15 | 62 |
| rs79066296 | 14 | 76391462 | T | C | 0.768 | -0.017 | 0.002 | 6.11E-14 | 59 |
| rs12882130 | 14 | 103878774 | G | C | 0.384 | -0.020 | 0.002 | 1.88E-24 | 102 |
| rs8018486 | 14 | 39818616 | G | A | 0.191 | -0.014 | 0.002 | 1.18E-08 | 33 |
| rs7144307 | 14 | 69533837 | C | T | 0.385 | -0.012 | 0.002 | 6.10E-10 | 37 |
| rs13316 | 14 | 93407301 | A | C | 0.430 | 0.012 | 0.002 | 3.66E-09 | 37 |
| rs147233090 | 15 | 44028047 | T | C | 0.025 | -0.045 | 0.006 | 3.95E-13 | 53 |
| rs5812543 | 15 | 51492850 | GT | G | 0.692 | 0.014 | 0.002 | 2.65E-10 | 41 |
| rs4383083 | 15 | 63080442 | A | G | 0.647 | 0.011 | 0.002 | 2.91E-08 | 31 |
| rs8042578 | 15 | 66992964 | G | C | 0.243 | 0.029 | 0.002 | 2.29E-38 | 170 |
| rs990315 | 15 | 69578811 | C | T | 0.623 | -0.012 | 0.002 | 5.07E-09 | 33 |
| rs74379684 | 15 | 94050205 | T | C | 0.075 | -0.027 | 0.004 | 4.39E-14 | 57 |
| rs2871865 | 15 | 99194896 | G | C | 0.116 | -0.049 | 0.003 | 3.40E-62 | 270 |
| rs17205463 | 15 | 62381413 | T | C | 0.448 | -0.026 | 0.002 | 4.21E-43 | 192 |
| rs36016415 | 15 | 86329195 | AC | A | 0.540 | -0.028 | 0.002 | 3.22E-47 | 209 |
| rs4965298 | 15 | 100802766 | T | C | 0.713 | -0.012 | 0.002 | 1.81E-08 | 32 |
| rs11070842 | 15 | 51624185 | C | T | 0.163 | -0.015 | 0.003 | 1.30E-08 | 32 |
| rs12907139 | 15 | 73521566 | A | G | 0.525 | -0.015 | 0.002 | 4.89E-15 | 61 |
| rs5742915 | 15 | 74336633 | C | T | 0.461 | 0.025 | 0.002 | 9.33E-39 | 170 |
| rs140657345 | 15 | 41615909 | TCCTC | T | 0.252 | -0.020 | 0.002 | 5.53E-20 | 83 |
| rs373736365 | 15 | 51360867 | G | T | 0.716 | 0.019 | 0.002 | 4.53E-18 | 74 |
| rs11633371 | 15 | 89356832 | T | G | 0.476 | 0.022 | 0.002 | 7.49E-30 | 129 |
| rs4932439 | 15 | 89401109 | G | A | 0.825 | -0.015 | 0.003 | 1.43E-09 | 36 |
| rs2174008 | 15 | 38510456 | C | G | 0.498 | -0.019 | 0.002 | 5.89E-24 | 102 |
| rs577289 | 15 | 40208911 | T | A | 0.281 | -0.013 | 0.002 | 5.39E-09 | 35 |
| rs72726050 | 15 | 42270059 | C | T | 0.096 | -0.019 | 0.003 | 1.84E-08 | 32 |
| rs12909863 | 15 | 75825822 | C | G | 0.251 | 0.019 | 0.002 | 6.05E-18 | 74 |
| rs713467 | 15 | 84646473 | A | G | 0.435 | 0.015 | 0.002 | 3.09E-14 | 59 |
| rs11629593 | 15 | 96033696 | G | T | 0.616 | -0.011 | 0.002 | 4.42E-08 | 30 |
| rs2663126 | 15 | 99563857 | A | G | 0.691 | -0.014 | 0.002 | 1.36E-11 | 44 |
| rs116092985 | 16 | 2160973 | G | A | 0.096 | -0.040 | 0.003 | 1.17E-34 | 148 |
| rs35811052 | 16 | 15128416 | G | A | 0.256 | -0.015 | 0.002 | 8.84E-12 | 45 |
| rs72771070 | 16 | 19993750 | T | C | 0.283 | 0.015 | 0.002 | 1.30E-12 | 51 |
| rs62033029 | 16 | 50107273 | A | G | 0.206 | -0.014 | 0.002 | 1.73E-09 | 38 |
| rs72801843 | 16 | 53508802 | A | T | 0.302 | 0.031 | 0.002 | 8.83E-52 | 222 |
| rs55872725 | 16 | 53809123 | T | C | 0.404 | 0.022 | 0.002 | 1.46E-30 | 137 |
| rs4985445 | 16 | 69867835 | G | A | 0.457 | -0.018 | 0.002 | 3.30E-20 | 85 |
| rs17818592 | 16 | 86088594 | C | T | 0.437 | -0.013 | 0.002 | 1.23E-11 | 46 |
| rs8054549 | 16 | 86417234 | A | C | 0.449 | -0.025 | 0.002 | 3.37E-39 | 175 |
| rs7185244 | 16 | 86546887 | C | T | 0.776 | -0.015 | 0.002 | 1.40E-10 | 41 |
| rs113478686 | 16 | 88850897 | CT | C | 0.229 | -0.024 | 0.002 | 3.76E-27 | 113 |
| rs12051245 | 16 | 783865 | C | T | 0.232 | 0.030 | 0.002 | 2.56E-40 | 185 |
| rs143076454 | 16 | 921179 | A | G | 0.019 | -0.050 | 0.007 | 1.06E-12 | 51 |
| rs246177 | 16 | 14380768 | T | C | 0.368 | 0.021 | 0.002 | 2.04E-27 | 114 |
| rs116008080 | 16 | 67254841 | A | G | 0.024 | -0.042 | 0.006 | 4.06E-11 | 43 |
| rs12926103 | 16 | 86371775 | A | G | 0.066 | 0.027 | 0.004 | 9.60E-13 | 51 |
| rs77364196 | 16 | 88353016 | A | G | 0.053 | -0.033 | 0.004 | 8.35E-15 | 59 |
| rs61528919 | 16 | 1004909 | C | T | 0.361 | 0.014 | 0.002 | 3.33E-12 | 49 |
| rs35816944 | 16 | 1828030 | A | G | 0.007 | -0.109 | 0.012 | 1.27E-20 | 86 |
| rs78457529 | 16 | 24950880 | T | C | 0.012 | -0.090 | 0.009 | 1.22E-24 | 106 |
| rs4788218 | 16 | 30055750 | C | T | 0.401 | 0.028 | 0.002 | 5.52E-46 | 209 |
| rs2240735 | 16 | 4027605 | T | C | 0.748 | 0.019 | 0.002 | 3.99E-18 | 74 |
| rs77809369 | 16 | 9052448 | T | C | 0.064 | 0.024 | 0.004 | 9.83E-10 | 37 |
| rs35268848 | 16 | 67927240 | A | C | 0.012 | 0.074 | 0.010 | 2.83E-13 | 53 |
| rs62070319 | 16 | 89573216 | T | C | 0.443 | -0.018 | 0.002 | 2.54E-20 | 81 |
| rs6502935 | 17 | 1650168 | T | C | 0.738 | -0.013 | 0.002 | 6.31E-09 | 32 |
| rs113146332 | 17 | 42565977 | C | G | 0.038 | 0.031 | 0.005 | 2.99E-10 | 40 |
| rs9894577 | 17 | 43223292 | A | G | 0.318 | -0.031 | 0.002 | 1.40E-52 | 240 |
| rs2005172 | 17 | 61996255 | C | A | 0.640 | 0.048 | 0.002 | 2.35E-128 | 576 |
| rs28485212 | 17 | 63550026 | T | C | 0.149 | -0.019 | 0.003 | 1.24E-12 | 48 |
| rs9890062 | 17 | 17434352 | A | G | 0.062 | 0.027 | 0.004 | 1.20E-11 | 47 |
| rs117972846 | 17 | 26947476 | G | T | 0.029 | 0.034 | 0.006 | 5.47E-09 | 35 |
| rs2289629 | 17 | 27959903 | A | G | 0.345 | -0.015 | 0.002 | 8.02E-14 | 55 |
| rs2019203 | 17 | 36908672 | A | C | 0.491 | 0.019 | 0.002 | 1.84E-23 | 99 |
| rs9905385 | 17 | 59498250 | G | A | 0.671 | -0.034 | 0.002 | 1.94E-63 | 287 |
| rs78766798 | 17 | 7517075 | C | T | 0.085 | 0.032 | 0.004 | 2.61E-20 | 83 |
| rs78378222 | 17 | 7571752 | G | T | 0.012 | 0.138 | 0.009 | 4.51E-56 | 252 |
| rs2112617 | 17 | 46977125 | A | G | 0.529 | -0.017 | 0.002 | 1.07E-18 | 77 |
| rs2676298 | 17 | 62726707 | T | C | 0.853 | -0.027 | 0.003 | 2.39E-23 | 99 |
| rs7220127 | 17 | 64545922 | C | T | 0.426 | -0.011 | 0.002 | 4.60E-08 | 31 |
| rs36000545 | 17 | 79093822 | G | A | 0.396 | -0.022 | 0.002 | 2.56E-29 | 121 |
| rs6505216 | 17 | 29206421 | T | G | 0.233 | -0.050 | 0.002 | 1.83E-101 | 469 |
| rs57791149 | 17 | 54222307 | C | T | 0.402 | -0.017 | 0.002 | 3.26E-19 | 83 |
| rs2521349 | 17 | 67503501 | A | G | 0.385 | 0.016 | 0.002 | 2.01E-15 | 67 |
| rs173135 | 17 | 68172326 | T | C | 0.115 | -0.034 | 0.003 | 3.25E-30 | 129 |
| rs12943867 | 17 | 79409710 | A | G | 0.337 | 0.018 | 0.002 | 7.57E-20 | 85 |
| rs9898189 | 17 | 80480516 | G | C | 0.645 | -0.016 | 0.002 | 1.88E-15 | 60 |
| rs57513571 | 17 | 2309130 | T | C | 0.200 | -0.019 | 0.002 | 7.02E-16 | 63 |
| rs11867855 | 17 | 18262584 | T | G | 0.237 | -0.013 | 0.002 | 2.93E-09 | 36 |
| rs4640244 | 17 | 21284223 | G | A | 0.399 | -0.020 | 0.002 | 3.81E-25 | 111 |
| rs72829852 | 17 | 46633974 | T | C | 0.062 | 0.031 | 0.004 | 3.74E-15 | 63 |
| rs2592208 | 17 | 67408873 | A | C | 0.513 | -0.012 | 0.002 | 5.52E-11 | 43 |
| rs113232639 | 18 | 20715656 | A | G | 0.493 | 0.033 | 0.002 | 4.79E-64 | 296 |
| rs8084413 | 18 | 22869123 | A | G | 0.469 | -0.013 | 0.002 | 3.25E-11 | 45 |
| rs2978362 | 18 | 32959397 | T | C | 0.533 | 0.011 | 0.002 | 2.85E-08 | 31 |
| rs71336393 | 18 | 57855319 | GA | G | 0.235 | 0.049 | 0.002 | 9.02E-105 | 454 |
| rs74494415 | 18 | 74972138 | T | C | 0.040 | -0.042 | 0.005 | 1.82E-17 | 72 |
| rs2347808 | 18 | 2750856 | A | G | 0.514 | -0.013 | 0.002 | 5.79E-11 | 43 |
| rs33973388 | 18 | 46611842 | T | G | 0.435 | 0.025 | 0.002 | 1.45E-38 | 172 |
| rs4940874 | 18 | 57105638 | G | A | 0.812 | 0.015 | 0.002 | 1.24E-09 | 38 |
| rs1786263 | 18 | 13116432 | T | G | 0.606 | -0.019 | 0.002 | 1.03E-22 | 100 |
| rs62103240 | 18 | 77650637 | A | G | 0.071 | 0.021 | 0.004 | 1.40E-08 | 33 |
| rs4121583 | 18 | 125075 | T | C | 0.619 | 0.012 | 0.002 | 4.62E-09 | 35 |
| rs568267 | 18 | 8799828 | T | C | 0.743 | 0.012 | 0.002 | 2.23E-08 | 31 |
| rs35073631 | 18 | 22696964 | C | T | 0.433 | 0.011 | 0.002 | 5.92E-09 | 35 |
| rs12962050 | 18 | 35179808 | A | G | 0.645 | 0.015 | 0.002 | 1.52E-14 | 59 |
| rs7229520 | 18 | 46516468 | A | G | 0.662 | -0.022 | 0.002 | 9.21E-29 | 125 |
| rs7228151 | 18 | 57181694 | C | T | 0.206 | -0.019 | 0.002 | 3.19E-15 | 65 |
| rs9957318 | 18 | 33039106 | G | A | 0.348 | 0.019 | 0.002 | 1.02E-20 | 87 |
| rs151123488 | 18 | 45982432 | CTGTGTGTGTG | C | 0.867 | 0.018 | 0.003 | 5.07E-10 | 38 |
| rs60389750 | 18 | 77182836 | T | C | 0.314 | -0.018 | 0.002 | 1.06E-16 | 69 |
| rs79441499 | 19 | 7201704 | T | C | 0.406 | -0.014 | 0.002 | 9.67E-13 | 53 |
| rs11373507 | 19 | 47404025 | GA | G | 0.629 | -0.011 | 0.002 | 1.12E-08 | 32 |
| rs45474992 | 19 | 47724564 | T | C | 0.036 | -0.062 | 0.005 | 2.17E-33 | 146 |
| rs10421750 | 19 | 50290604 | T | G | 0.312 | -0.015 | 0.002 | 5.73E-12 | 48 |
| rs16989695 | 19 | 4505445 | A | G | 0.517 | -0.014 | 0.002 | 1.93E-13 | 54 |
| rs12150907 | 19 | 4940630 | A | G | 0.198 | -0.022 | 0.002 | 7.22E-20 | 83 |
| rs10948 | 19 | 10754905 | T | G | 0.664 | -0.025 | 0.002 | 3.43E-36 | 159 |
| rs2607234 | 19 | 35563834 | G | A | 0.948 | -0.030 | 0.004 | 2.00E-12 | 49 |
| rs117203652 | 19 | 49857801 | A | G | 0.030 | -0.035 | 0.006 | 4.32E-10 | 40 |
| rs4807472 | 19 | 3448842 | C | T | 0.676 | -0.016 | 0.002 | 8.18E-15 | 62 |
| rs12461874 | 19 | 17180358 | A | C | 0.278 | -0.018 | 0.002 | 1.26E-17 | 74 |
| rs116919274 | 19 | 17359808 | A | G | 0.046 | 0.027 | 0.005 | 3.52E-09 | 35 |
| rs111901094 | 19 | 19513570 | T | G | 0.182 | -0.025 | 0.003 | 4.04E-24 | 102 |
| rs75702986 | 19 | 35566151 | A | G | 0.186 | -0.016 | 0.003 | 3.14E-11 | 43 |
| rs4252548 | 19 | 55879672 | T | C | 0.022 | -0.075 | 0.007 | 2.96E-31 | 134 |
| rs8112948 | 19 | 2175005 | A | T | 0.286 | -0.030 | 0.002 | 4.24E-42 | 182 |
| rs350832 | 19 | 4069426 | A | G | 0.771 | -0.017 | 0.002 | 3.44E-13 | 51 |
| rs11260035 | 19 | 7898957 | A | G | 0.276 | 0.015 | 0.002 | 1.86E-12 | 51 |
| rs11672848 | 19 | 37570704 | T | C | 0.525 | -0.017 | 0.002 | 7.73E-19 | 81 |
| rs2287821 | 19 | 33935102 | T | C | 0.509 | -0.015 | 0.002 | 6.95E-16 | 65 |
| rs147110934 | 19 | 55993436 | T | G | 0.024 | -0.072 | 0.006 | 9.39E-32 | 136 |
| rs6054390 | 20 | 6592094 | A | G | 0.628 | -0.019 | 0.002 | 1.45E-21 | 88 |
| rs73125634 | 20 | 20069826 | T | G | 0.278 | -0.020 | 0.002 | 5.11E-20 | 86 |
| rs12185775 | 20 | 20293769 | C | G | 0.117 | -0.017 | 0.003 | 3.66E-08 | 31 |
| rs112021215 | 20 | 62867186 | C | T | 0.201 | -0.015 | 0.003 | 5.99E-09 | 35 |
| rs4815952 | 20 | 6934897 | C | T | 0.522 | -0.016 | 0.002 | 1.24E-16 | 72 |
| rs35963161 | 20 | 49210635 | A | G | 0.476 | -0.016 | 0.002 | 7.46E-16 | 68 |
| rs77447813 | 20 | 50827041 | C | G | 0.087 | 0.022 | 0.003 | 3.21E-11 | 43 |
| rs2236096 | 20 | 3266319 | C | T | 0.233 | 0.018 | 0.002 | 1.29E-15 | 61 |
| rs6142059 | 20 | 32544327 | C | T | 0.493 | 0.012 | 0.002 | 1.19E-09 | 37 |
| rs143384 | 20 | 34025756 | G | A | 0.404 | 0.073 | 0.002 | 1.00E-200 | 1456 |
| rs80132799 | 20 | 62322896 | T | C | 0.068 | 0.023 | 0.004 | 1.31E-09 | 37 |
| rs6054491 | 20 | 6709535 | G | C | 0.240 | -0.014 | 0.002 | 2.15E-10 | 42 |
| rs684905 | 20 | 10472790 | T | C | 0.418 | -0.012 | 0.002 | 7.10E-10 | 39 |
| rs6082354 | 20 | 21217976 | C | A | 0.668 | -0.024 | 0.002 | 1.19E-32 | 144 |
| rs4287835 | 20 | 31457337 | C | T | 0.534 | 0.015 | 0.002 | 9.94E-15 | 60 |
| rs1291114 | 20 | 35500850 | C | G | 0.890 | 0.017 | 0.003 | 1.95E-08 | 31 |
| rs57696574 | 20 | 54884826 | C | A | 0.398 | 0.017 | 0.002 | 4.50E-18 | 75 |
| rs34879158 | 20 | 32300634 | C | A | 0.263 | -0.036 | 0.002 | 1.55E-63 | 272 |
| rs6028716 | 20 | 38547459 | A | G | 0.259 | -0.021 | 0.002 | 4.58E-22 | 91 |
| rs6066122 | 20 | 45558573 | G | C | 0.763 | 0.013 | 0.002 | 1.76E-08 | 30 |
| rs13037813 | 20 | 47750588 | C | T | 0.239 | 0.029 | 0.002 | 1.71E-39 | 176 |
| rs73197345 | 21 | 36770120 | A | T | 0.137 | 0.021 | 0.003 | 3.55E-14 | 57 |
| rs12483401 | 21 | 35443829 | C | T | 0.022 | -0.039 | 0.007 | 9.22E-09 | 33 |
| rs2230033 | 21 | 39671476 | A | G | 0.564 | -0.027 | 0.002 | 3.49E-43 | 195 |
| rs112153300 | 21 | 47547474 | A | G | 0.089 | 0.026 | 0.003 | 7.05E-15 | 59 |
| rs35631698 | 21 | 16549469 | GC | G | 0.375 | 0.015 | 0.002 | 2.59E-13 | 53 |
| rs2212926 | 21 | 38066883 | A | C | 0.211 | -0.022 | 0.002 | 7.75E-21 | 91 |
| rs4818280 | 21 | 18114472 | T | C | 0.627 | -0.012 | 0.002 | 2.84E-10 | 38 |
| rs9610447 | 22 | 20768891 | T | C | 0.747 | 0.015 | 0.002 | 5.29E-12 | 48 |
| rs5753518 | 22 | 31631314 | A | G | 0.090 | 0.024 | 0.003 | 5.04E-13 | 54 |
| rs7286917 | 22 | 39860868 | G | A | 0.746 | 0.017 | 0.002 | 5.18E-14 | 55 |
| rs41311445 | 22 | 42070374 | C | A | 0.096 | -0.033 | 0.003 | 4.73E-24 | 105 |
| rs5763821 | 22 | 30549071 | C | A | 0.391 | 0.019 | 0.002 | 1.06E-21 | 91 |
| rs6000886 | 22 | 38176670 | C | T | 0.648 | 0.013 | 0.002 | 5.88E-11 | 43 |
| rs8136517 | 22 | 46439433 | C | T | 0.065 | 0.027 | 0.004 | 6.58E-12 | 47 |
| rs165849 | 22 | 19958669 | A | G | 0.698 | 0.016 | 0.002 | 4.57E-14 | 56 |
| rs10453441 | 22 | 46363739 | G | A | 0.402 | -0.014 | 0.002 | 9.10E-13 | 48 |
| rs28379706 | 22 | 50728062 | C | T | 0.393 | 0.011 | 0.002 | 4.49E-09 | 32 |

Abbreviations: SNPs, single-nucleotide polymorphisms; EAF, effect allele frequency; Se, standard error.

**Supplementary Figure S1.** Scatter plot (A), funnel plot (B), and leave-one-out analysis (C) of the causal effect of right grip on KOA risk.

A
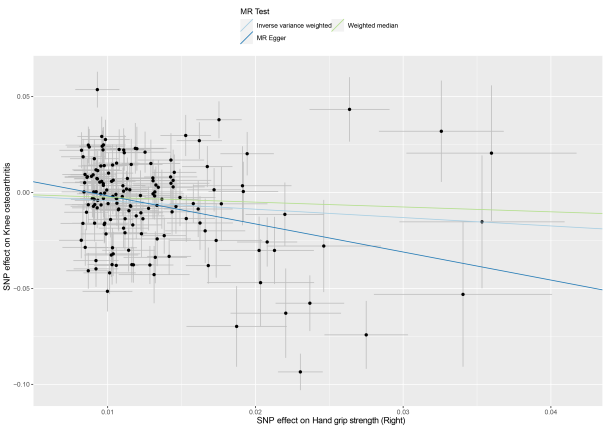
B
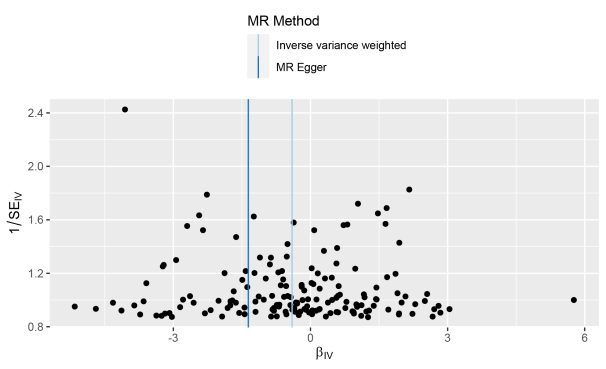


C
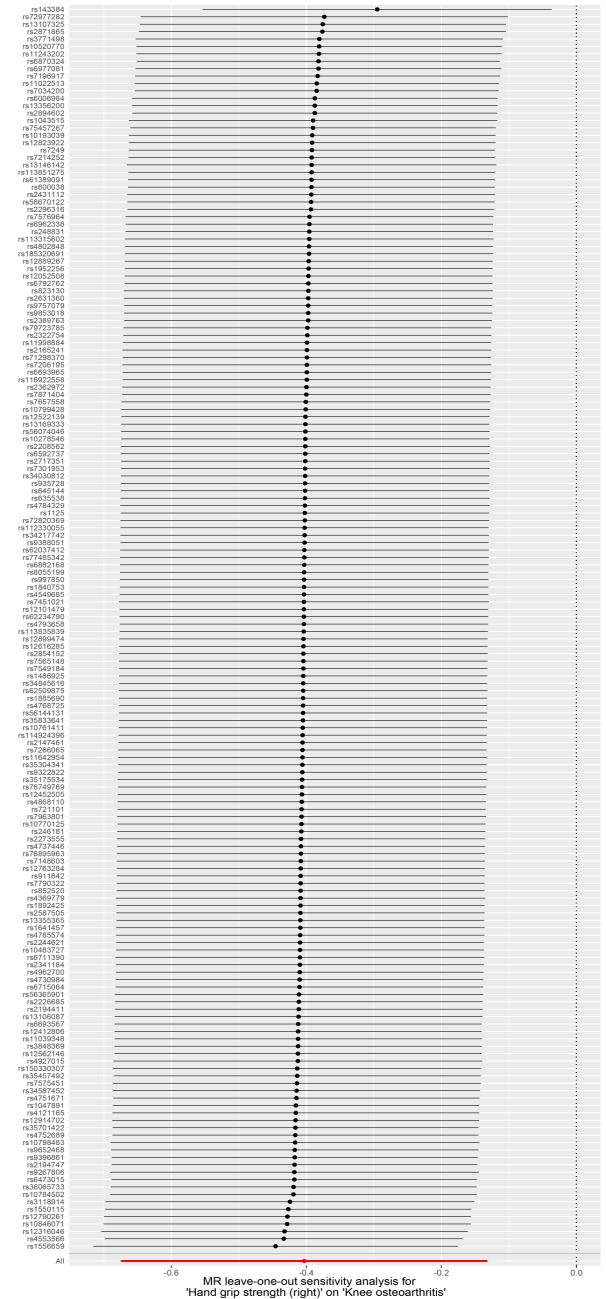


**Supplementary Figure S2.** Scatter plot (A), funnel plot (B), and leave-one-out analysis (C) of the causal effect of right grip on HOA risk.

A
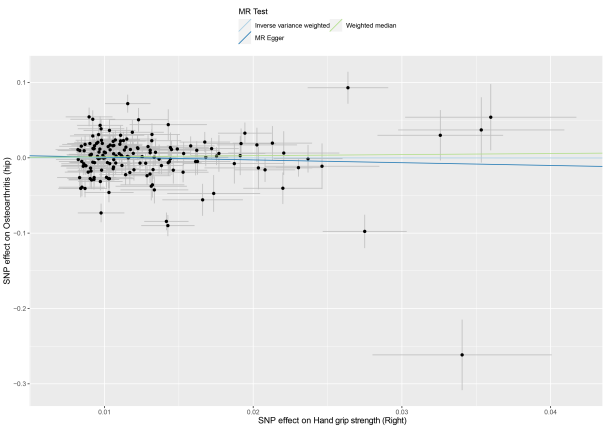
B
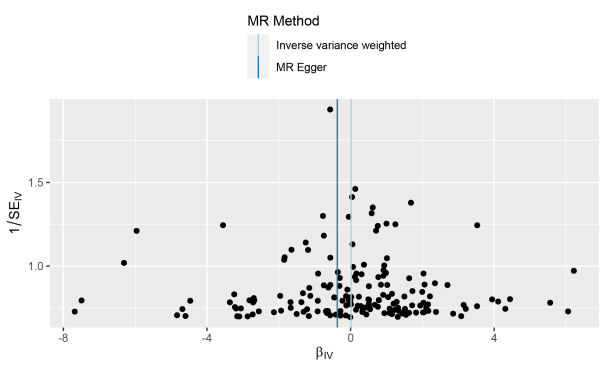


C
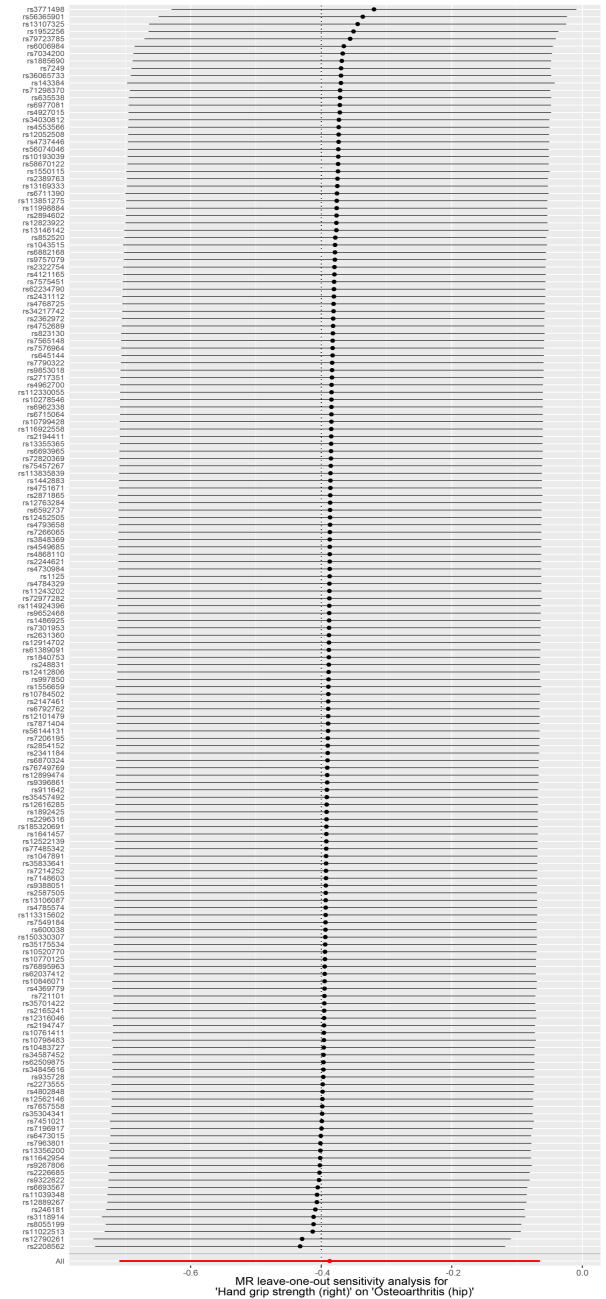


**Supplementary Figure S3.** Scatter plot (A), funnel plot (B), and leave-one-out analysis (C) of the causal effect of right grip on Total OA risk.

A
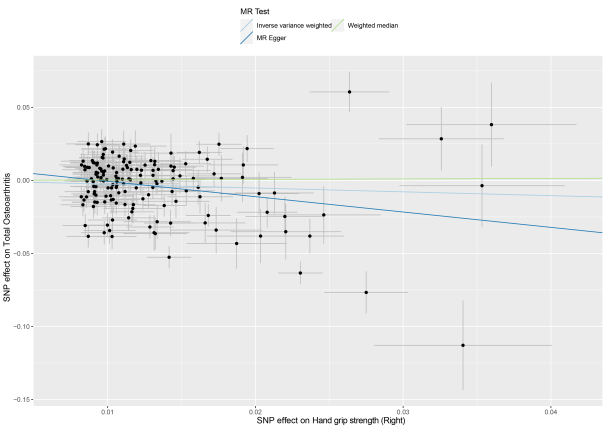
B
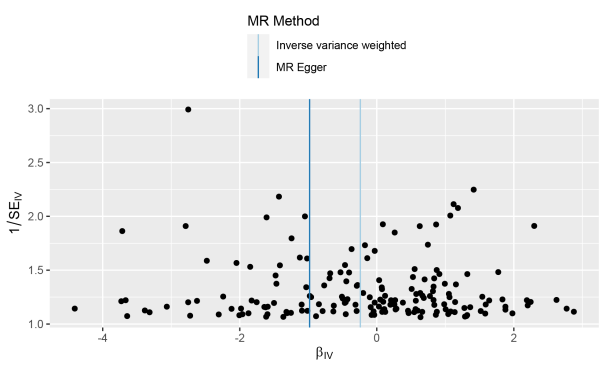


C
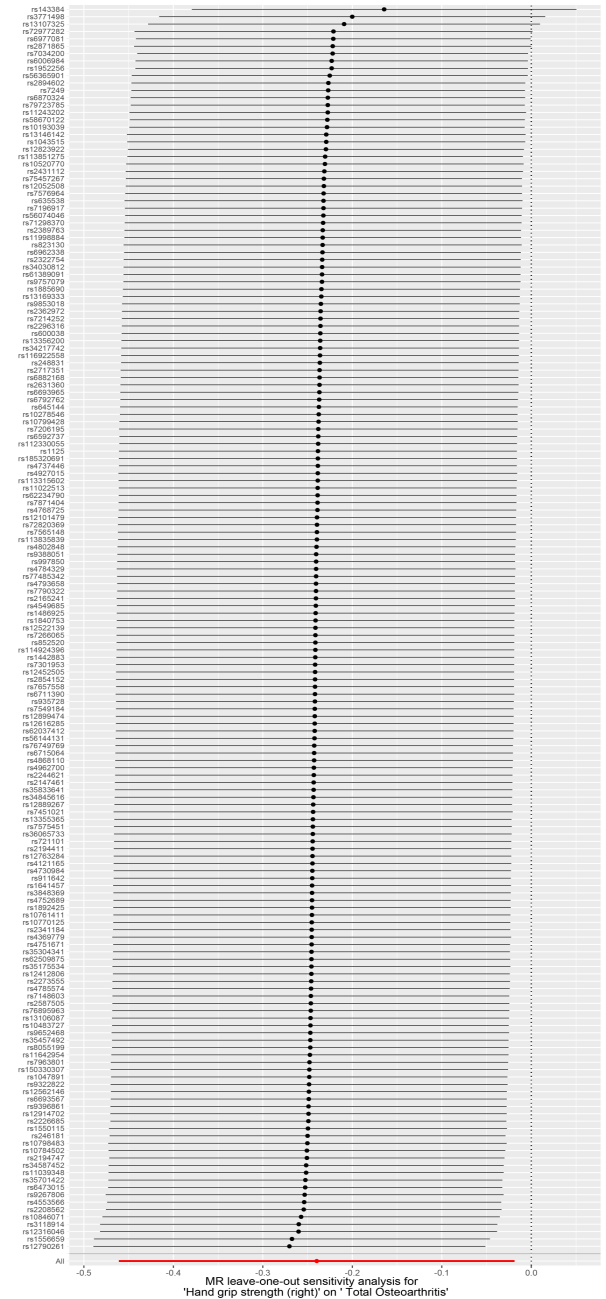


**Supplementary Figure S4.** Scatter plot (A), funnel plot (B), and leave-one-out analysis (C) of the causal effect of left grip on KOA risk.

A
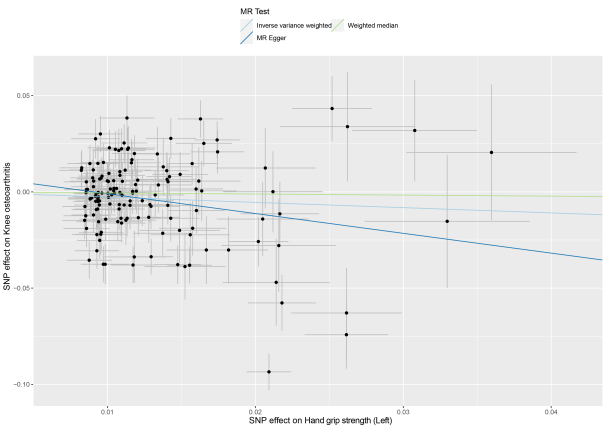
B
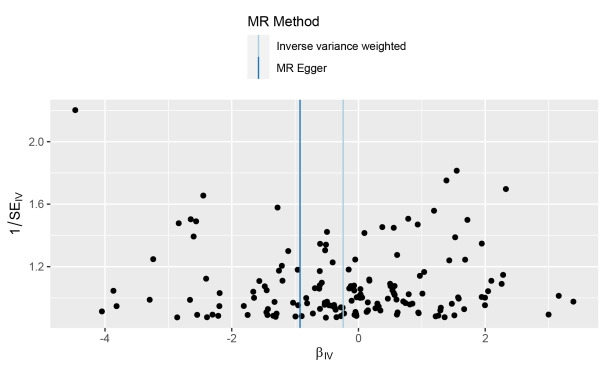


C
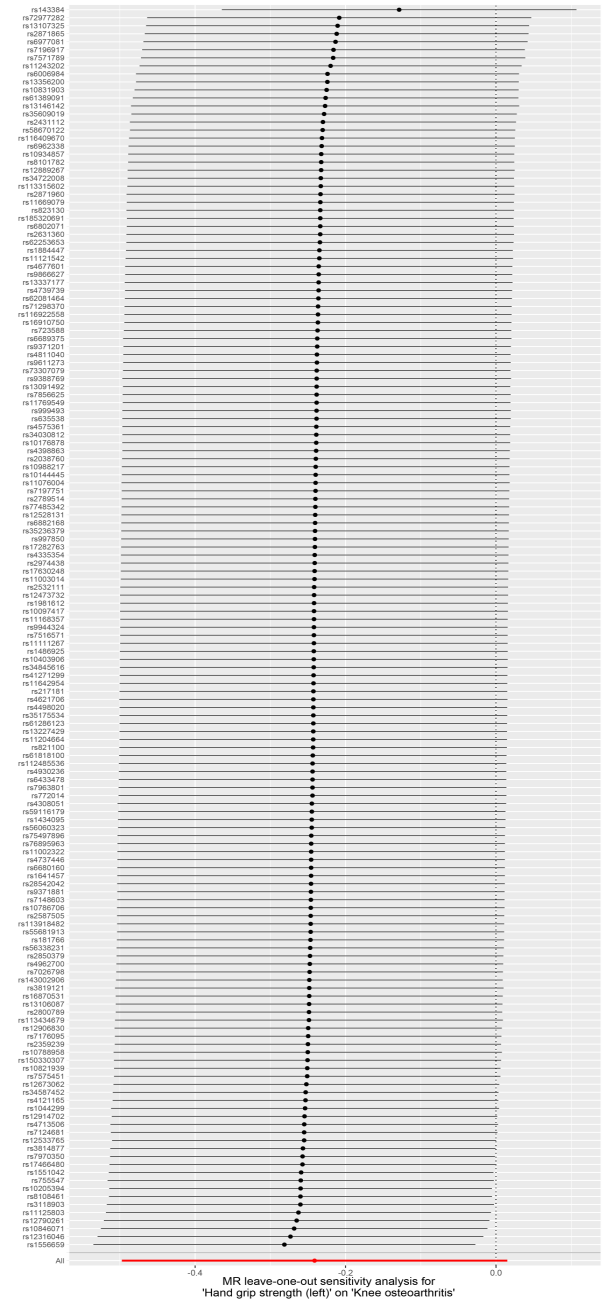


**Supplementary Figure S5.** Scatter plot (A), funnel plot (B), and leave-one-out analysis (C) of the causal effect of left grip on HOA risk.

A
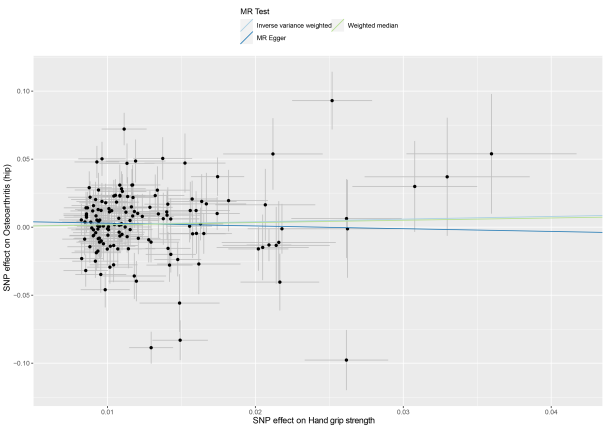
B
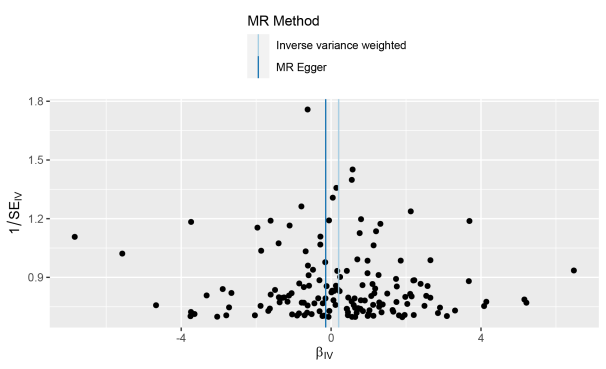


C
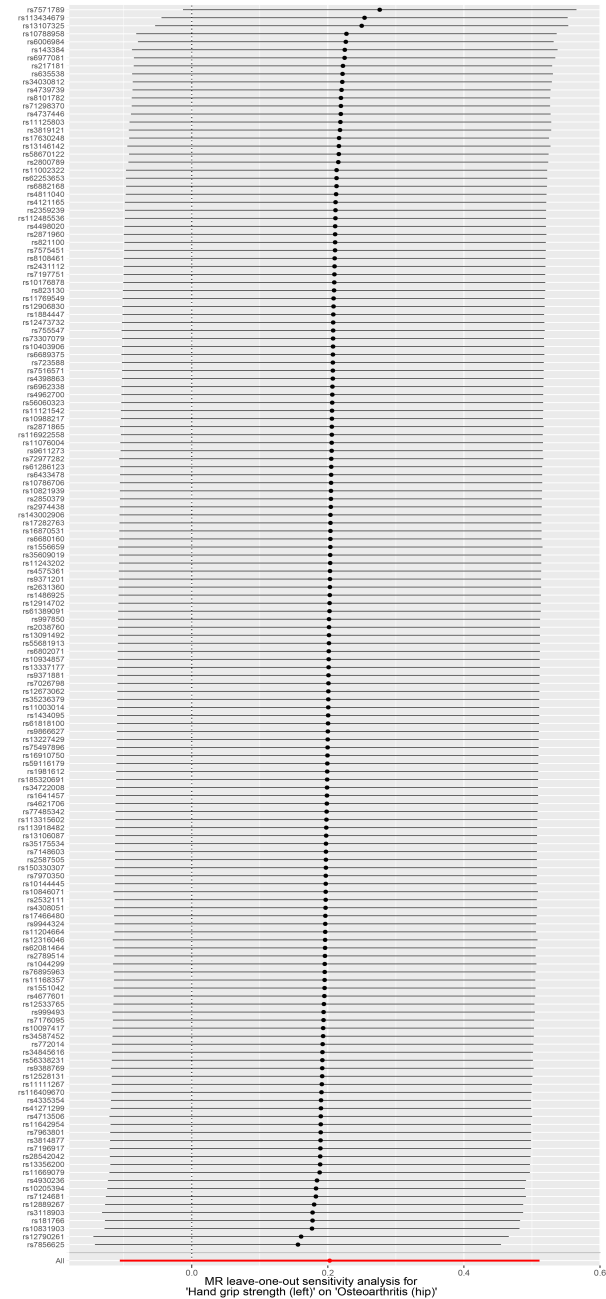


**Supplementary Figure S6.** Scatter plot (A), funnel plot (B), and leave-one-out analysis (C) of the causal effect of left grip on Total OA risk.

A
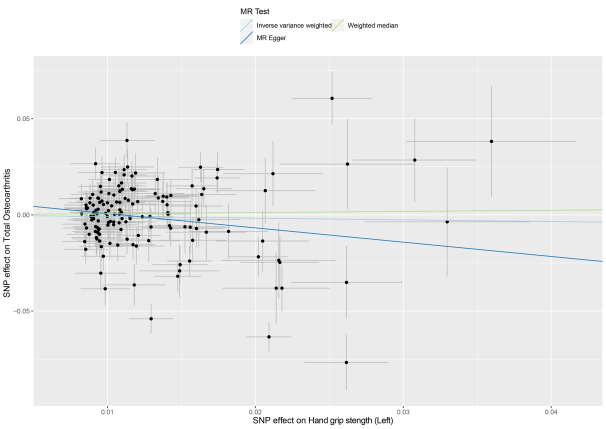
B
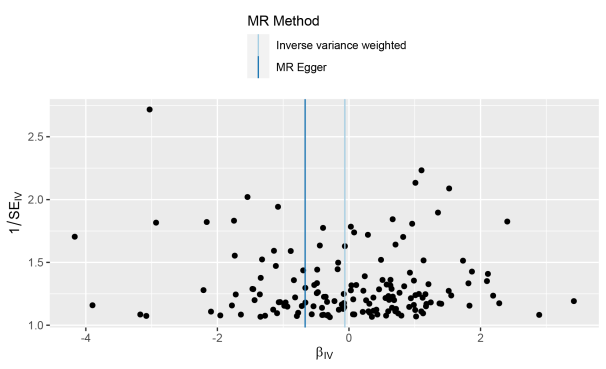


C
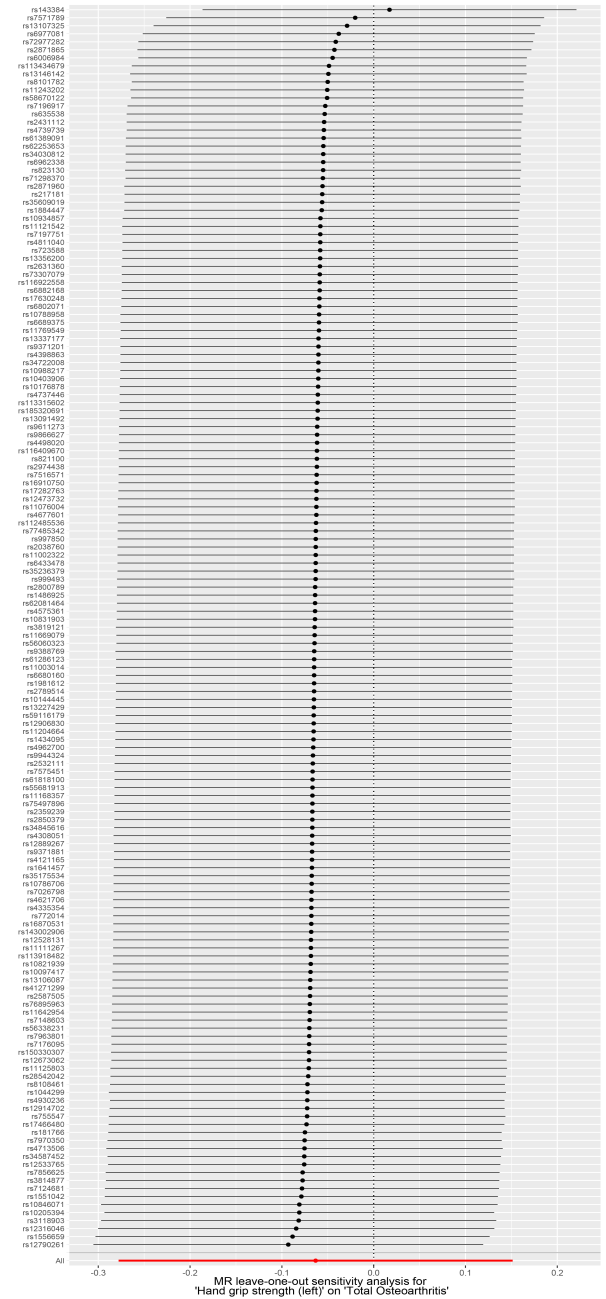


**Supplementary Figure S7.** Scatter plot (A), funnel plot (B), and leave-one-out analysis (C) of the causal effect of ALM on KOA risk.

A
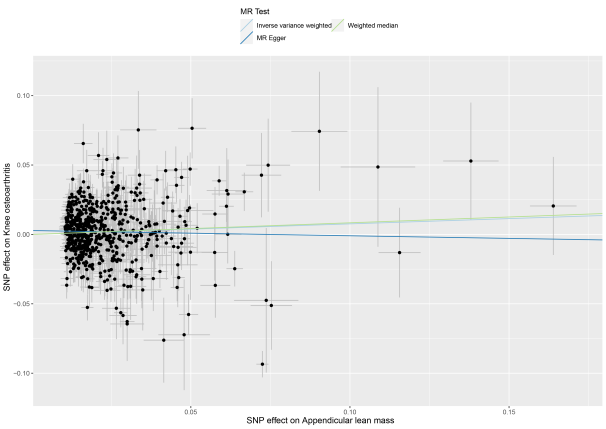
B
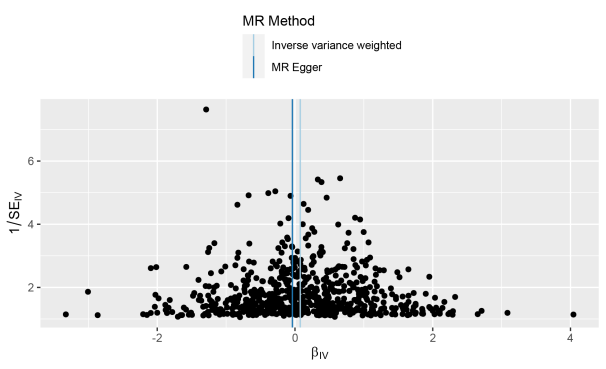


C
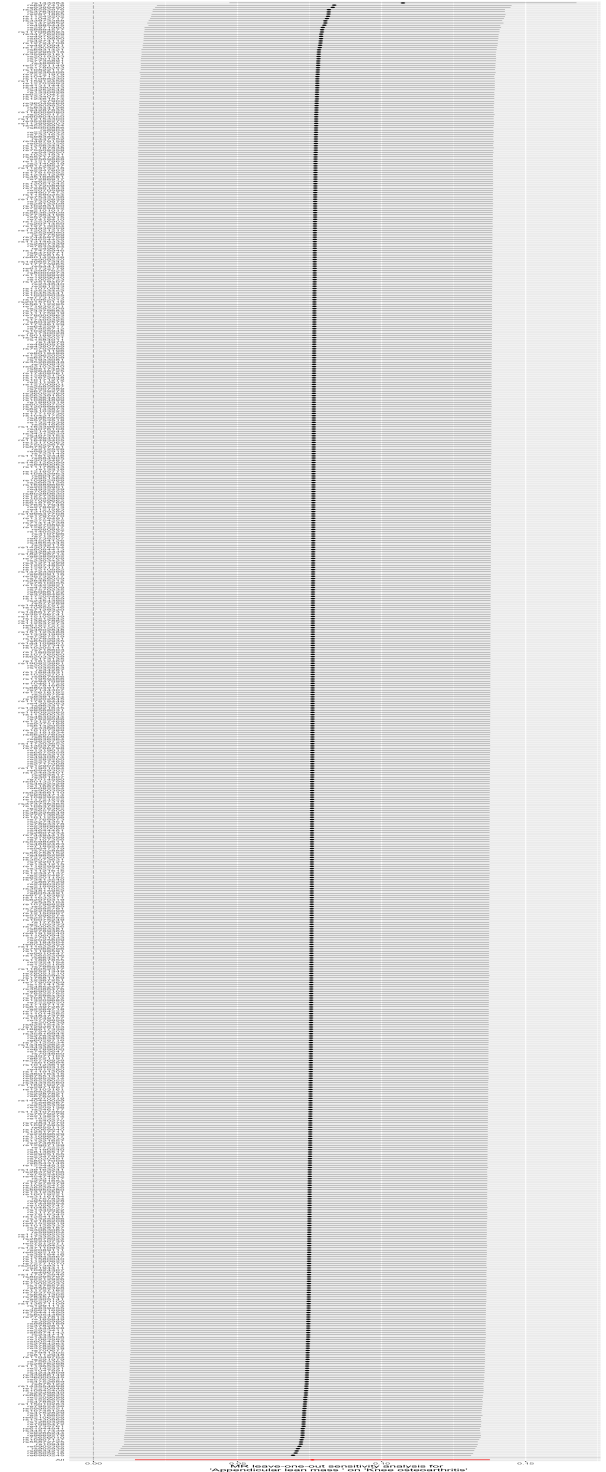


**Supplementary Figure S8.** Scatter plot (A), funnel plot (B), and leave-one-out analysis (C) of the causal effect of ALM on HOA risk.

**A
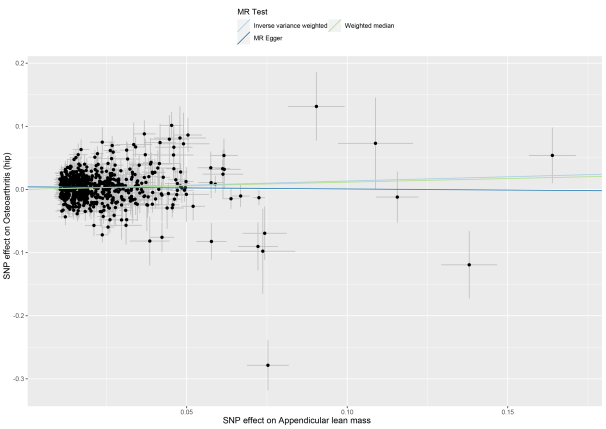
B
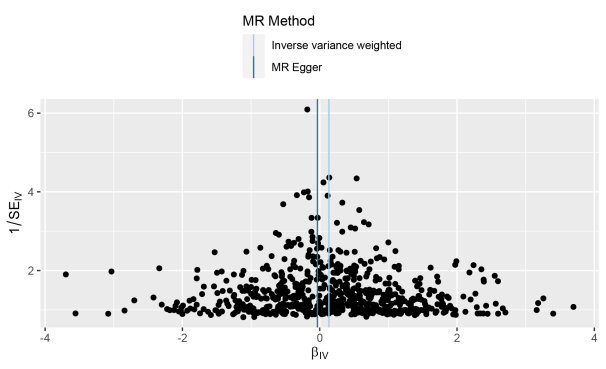
**

**C
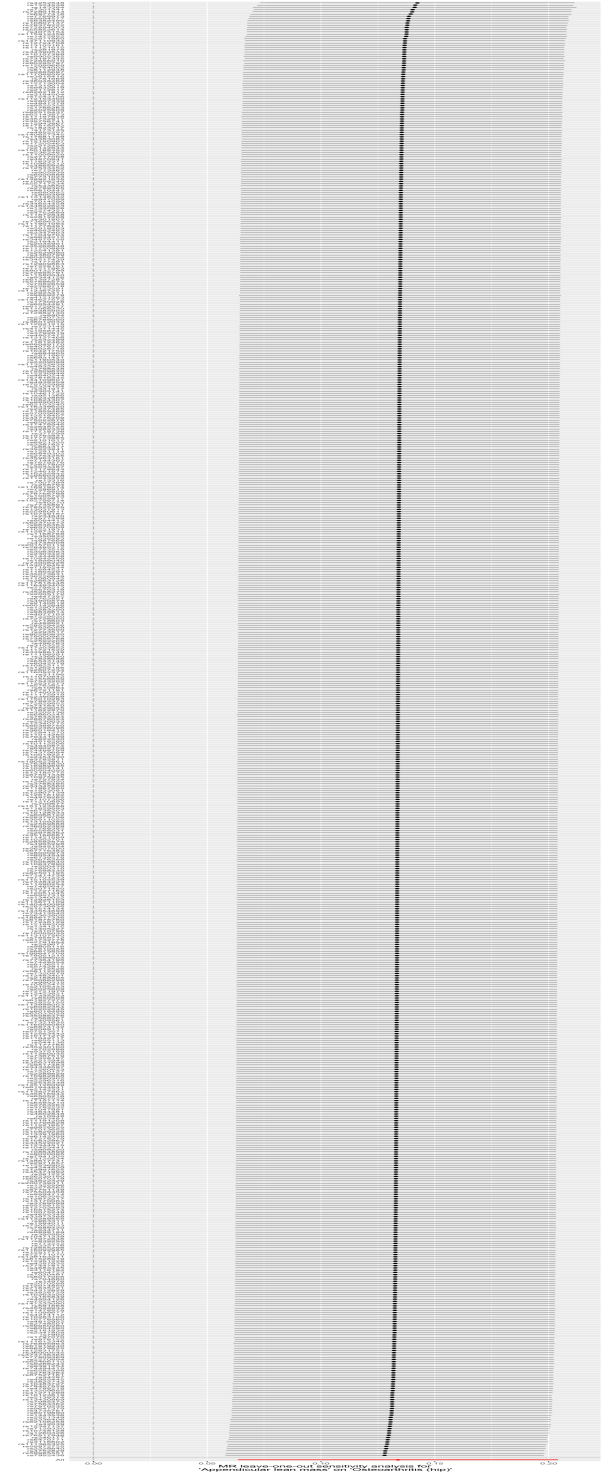
**

**Supplementary Figure S9.** Scatter plot (A), funnel plot (B), and leave-one-out analysis (C) of the causal effect of ALM on Total OA risk.

A
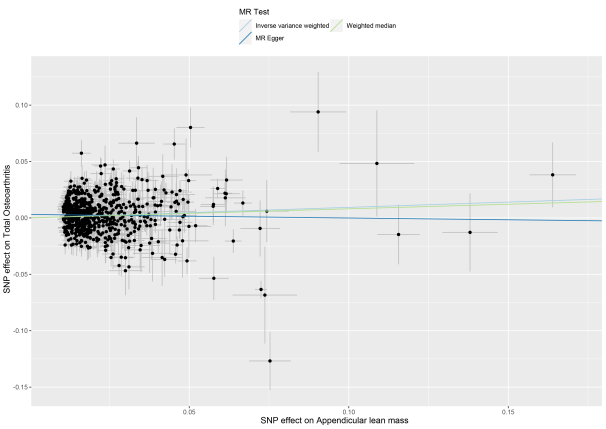
B
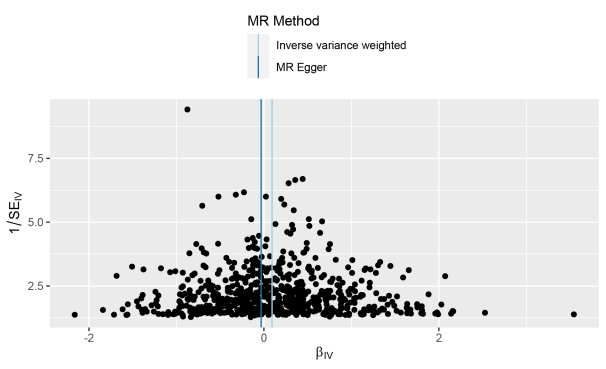


C
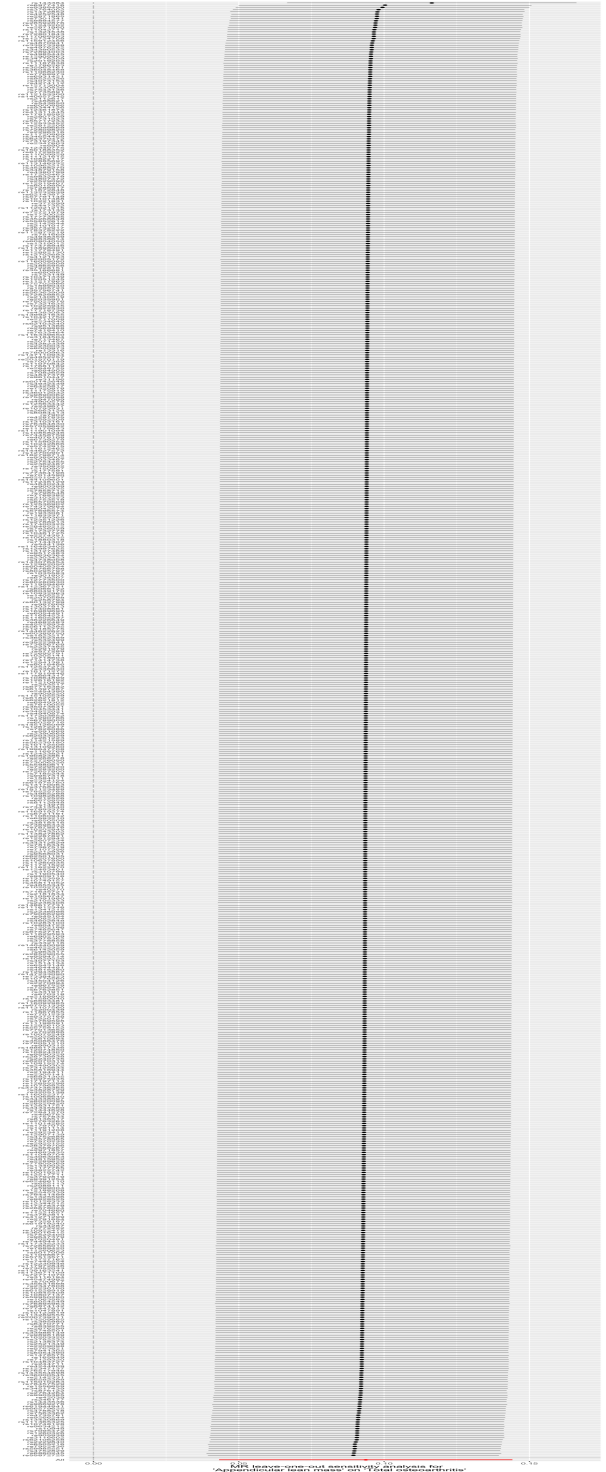

Supplement: Supplementary file 1 — Additional file 1. Detailed characteristics of GWAS associated with exposures and outcomes in the study. Scatter plot, funnel plot, and leave-one-out analysis of the causal effect of sarcopenia on OA risk. [file 13018_2023_3960_MOESM1_ESM.docx]
